# Chemiluminescence Detection of Hydrogen Sulfide Release by β-Lactamase-Catalyzed β-Lactam Biodegradation: Unprecedented Pathway for Monitoring β-Lactam Antibiotic Bacterial Resistance

**DOI:** 10.1021/acs.bioconjchem.1c00149

**Published:** 2021-04-25

**Authors:** Sachin Popat Gholap, Chunyan Yao, Ori Green, Matej Babjak, Pavol Jakubec, Tomáš Malatinský, Julian Ihssen, Lukas Wick, Urs Spitz, Doron Shabat

**Affiliations:** † School of Chemistry, Raymond and Beverly Sackler Faculty of Exact Sciences, Tel Aviv University, Tel Aviv 69978 Israel; ‡ Biosynth Carbosynth, Rietlistrasse 4 Postfach 125 9422 Staad, Switzerland; § Department of Organic Chemistry, Faculty of Chemical and Food Technology, Slovak University of Technology in Bratislava, Radlinského 9, 81237 Bratislava, Slovakia; ∥ Auchem s.r.o., A. Hlinku 1452/3, 022 01 Čadca, Slovakia

## Abstract

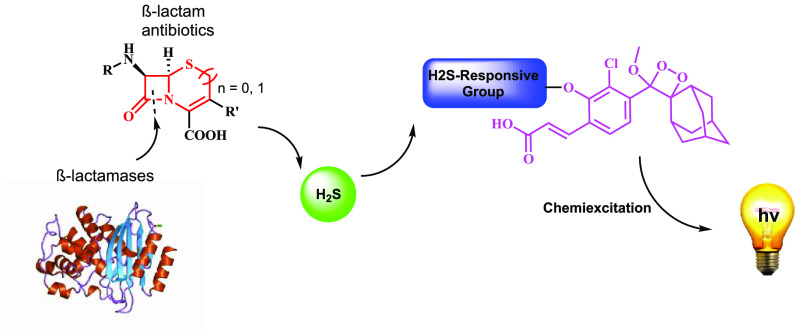

β-Lactamase positive bacteria represent a growing threat to human health because of their resistance to commonly used antibiotics. Therefore, development of new diagnostic methods for identification of β-lactamase positive bacteria is of high importance for monitoring the spread of antibiotic-resistant bacteria. Here, we report the discovery of a new biodegradation metabolite (H_2_S), generated through β-lactamase-catalyzed hydrolysis of β-lactam antibiotics. This discovery directed us to develop a distinct molecular technique for monitoring bacterial antibiotic resistance. The technique is based on a highly efficient chemiluminescence probe, designed for detection of the metabolite, hydrogen sulfide, that is released upon biodegradation of β-lactam by β-lactamases. Such an assay can directly indicate if antibiotic bacterial resistance exists for a certain examined β-lactam. The assay was successfully demonstrated for five different β-lactam antibiotics and eight β-lactam resistant bacterial strains. Importantly, in a functional bacterial assay, our chemiluminescence probe was able to clearly distinguish between a β-lactam resistant bacterial strain and a sensitive one. As far as we know, there is no previous documentation for such a biodegradation pathway of β-lactam antibiotics. Bearing in mind the data obtained in this study, we propose that hydrogen sulfide should be considered as an emerging β-lactam metabolite for detection of bacterial resistance.

## Introduction

To date, β-lactams remain the most widely utilized antibiotics because of their relatively high efficiency, low cost, ease of delivery and minimal side effects.^1^ β-Lactamase positive bacteria represent a growing threat to human health because of their resistance to commonly used antibiotics.^2^ Of particular concern are bacteria expressing extended spectrum β-lactamases such as cephalosporinases or carbapenemases; some of these strains are capable of inactivating almost all known β-lactam antibiotics.^3−5^ Therefore, development of diagnostic tools for identification of β-lactamase positive bacteria is of high importance for monitoring the spread of antibiotic-resistant bacteria in regard to public health. Such diagnostic tools are also imperative to guide the choice of potentially active antibiotics for effective treatment.

Most assays for detection of antibiotic-resistant bacteria still rely on measurement of growth in the presence of antibiotics.^6^ Such assays typically require incubation times of 10 to 48 h even after initial isolation of the infecting strain. For direct detection of β-lactamase activity, artificial substrates or reagents leading to either color, fluorescence, or chemiluminescence signal formation are required.^7−11^ During the last four years, we have developed new chemiluminescence luminophores, which are highly emissive under aqueous conditions.^12−19^ Very recently, we reported a new sensitive chemiluminescent probe for detection of carbapenemase activity, which incorporates such a highly emissive luminophore.^20^ We demonstrated the ability of the probe to detect a number of clinically relevant carbapenemases in bacterial cultures, such as those used for clinical diagnosis.

About 40 years ago, while exploring the degradation of antibiotics, it has been reported that certain β-lactam cephalosporin antibiotics decompose to release hydrogen sulfide under strong alkaline hydrolysis conditions.^21,22^ This observation has encouraged us to investigate whether antibiotics containing sulfur could release hydrogen sulfide upon their biodegradation by β-lactamases. Detection of such released hydrogen sulfide can be harnessed to develop a new diagnostic method for detection of β-lactam antibiotic resistance in bacteria. Here, we report a distinctive approach to detect bacterial antibiotic resistance by using chemiluminescence probes designed for detection of hydrogen sulfide, generated upon biodegradation of β-lactam antibiotics by β-lactamases.

## Results and Discussion

**Figure 1:**
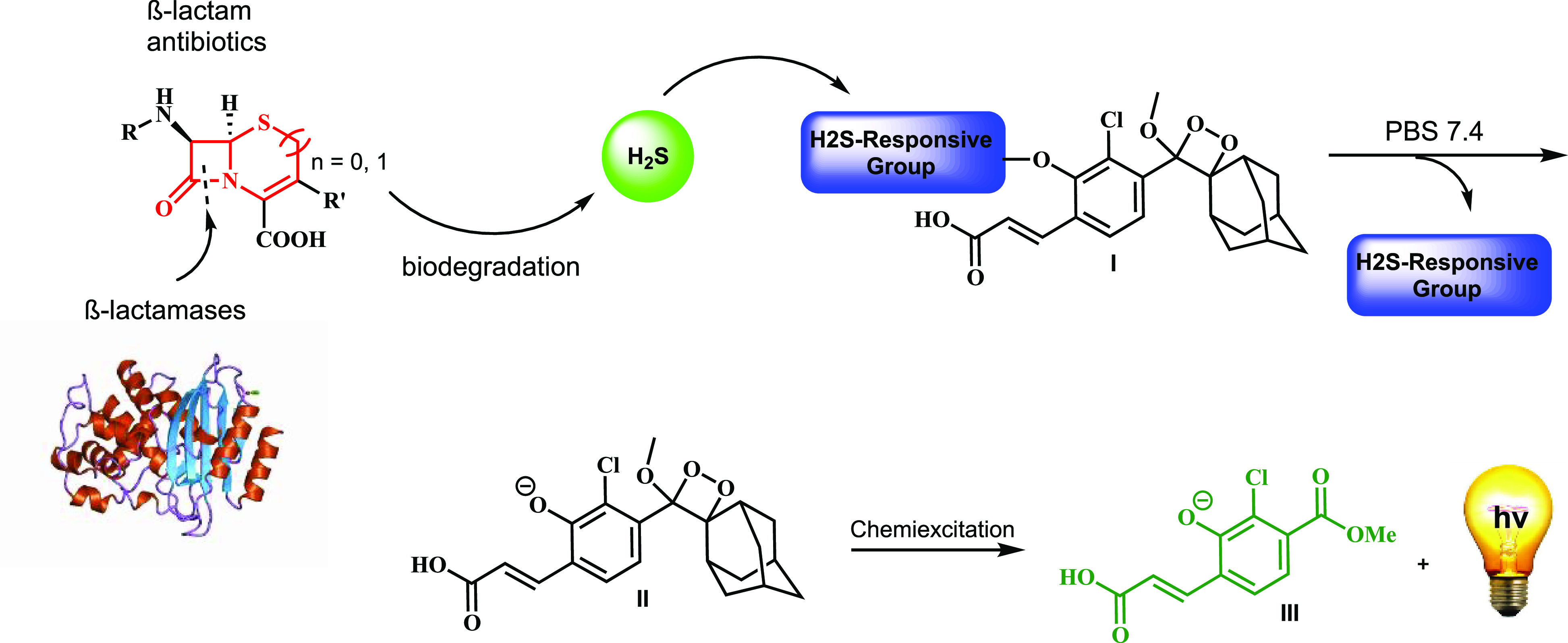
β-Lactamase catalyzed biodegradation of β-lactam antibiotics to release hydrogen sulfide, which subsequently activates a chemiluminescent probe.

The general description of our approach to detect bacterial antibiotic resistance, based on hydrogen sulfide release, is presented in Figure 1. Hydrogen sulfide is generated by β-lactamase catalyzed biodegradation of β-lactam antibiotics. The released H_2_S is then reacting with a specific responsive substrate of chemiluminescent probe **I**, to generate phenoxy-dioxetane **II**. The latter undergoes rapid chemiexcitation to produce benzoate **III** and emission of a green photon. Consequently, only bacteria with antibiotic resistance through the β-lactamase pathway are expected to produce a light emission signal upon incubation with chemiluminescent probe **I**.

**Figure 2:**
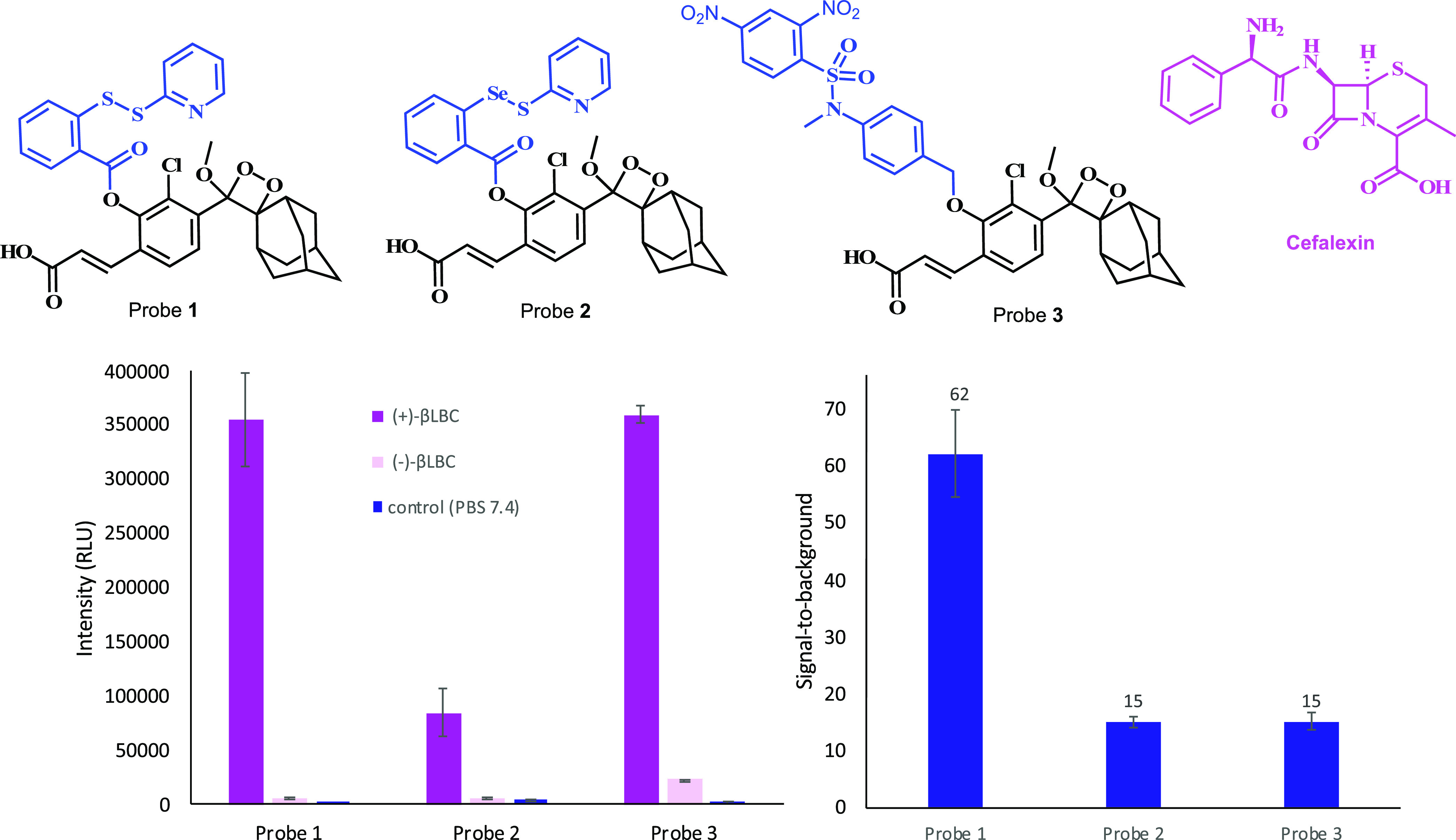
Chemiluminescence light emission [left] and signal-to-background [right] (after 2.5 h) of probes **1**, **2**, and **3** [25 μM] in PBS (pH 7.4) at 27 °C, upon biodegradation of cefalexin [1 mM] in the presence and absence of β-lactamase from *Bacillus cereus* [10 U/mL]. Probes were preincubated for 45 min in PBS (pH 7.4) prior to use; cefalexin with β-lactamase **βLBC** was incubated for 30 min prior to measurement.

In order to test whether hydrogen sulfide can indeed be released upon biodegradation of β-lactam antibiotics by β-lactamases, we synthesized three analogous chemiluminescent probes, each equipped with a different H_2_S responsive substrate (Figure 2). Probe **1** and probe **2** are composed of an adamantyl-phenoxy-dioxetane luminophore masked by a disulfide and seleno-sulfide^23^ H_2_S-responsive groups.^24^ Probe **3**, also composed of adamantyl-phenoxy-dioxetane luminophore, was masked with a dinitro-sulfonyl-amide group, which is a ubiquitous sulfhydryl responsive substrate.^12^

The probes were incubated with the β-lactam antibiotic cefalexin, in the presence and in the absence of β-lactamase from *Bacillus cereus* (**βLBC**) in PBS 7.4. Clearly, all three probes generated a significant turn-ON light emission signal in the presence the β-lactamase **βLBC**. However, probe **1** produced 62-fold signal-to-noise (S/N) value, while probe **2** and probe **3** produced S/N of only 15-fold. These data provided the first indication that biodegradation of the evaluated β-lactam cefalexin by β-lactamase can indeed result in release of hydrogen sulfide.

**Figure 3:**
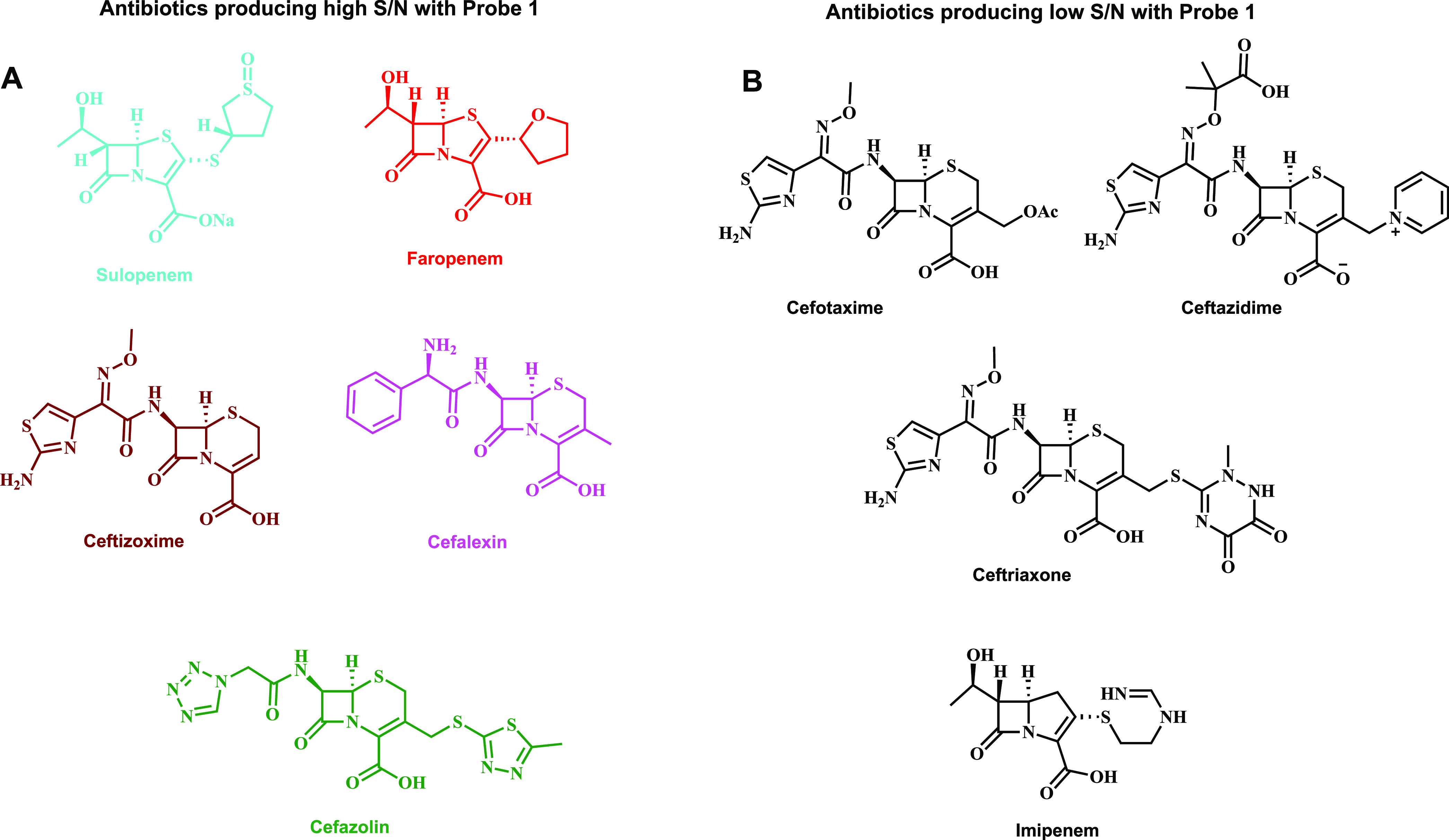
Chemical structure of β-lactam antibiotics producing high S/N values with probe **1** (**A**) vs β-lactam antibiotics producing low S/N values (**B**).

Given the obtained findings, probe **1** was selected for further evaluation studies. Nine different β-lactam antibiotics that incorporate sulfur in their molecular structure (Figure 3) were initially tested for their ability to produce a substantial S/N value in the presence of probe **1** and **βLBC**. Five out of the nine evaluated β-lactam antibiotics (Figure 3A) were able to present substantially high S/N values, while the other four antibiotics (Figure 3B) showed almost no difference (see Supporting Information, Figure S3). Therefore, further measurements were performed only with the five β-lactam antibiotics: sulopenem, faropenem, cefalexin, ceftizoxime, and cefazolin.

**Figure 4:**
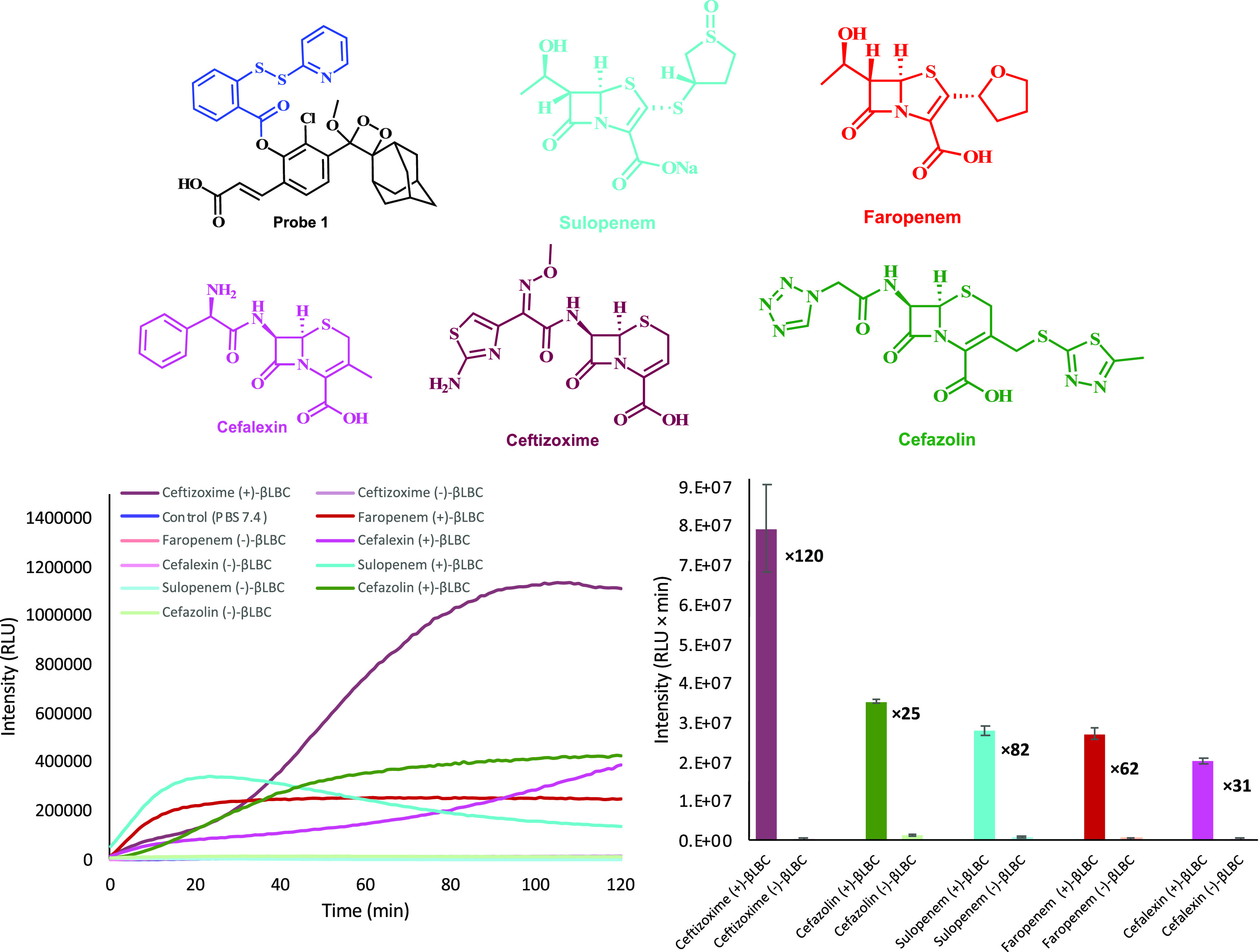
Chemiluminescence kinetic profiles [left] and total light emission [right] (over 2 h) produced by probe **1** [25 μM] in PBS (pH 7.4) at 27 °C and five different antibiotics [1 mM] in the presence and absence of **βLBC** [10 U/mL]. Probe **1** was preincubated for 45 min in PBS (pH 7.4) prior to use; antibiotics with **βLBC** were incubated for 30 min prior to measurement.

The selected five β-lactam antibiotics were incubated in the presence of **βLBC** with chemiluminescent probe **1** in PBS 7.4 and the light emission kinetic profile produced by the probe was measured for 2 h (Figure 4). Indeed, all five β-lactam antibiotics, upon incubation with **βLBC**, exhibited a significant light emission signal with S/N value of 25–120-fold (various kinetic profile behavior). The light emission signal produced by probe **1** and the β-lactam antibiotics, in the absence of **βLBC**, was significantly lower.

**Figure 5:**
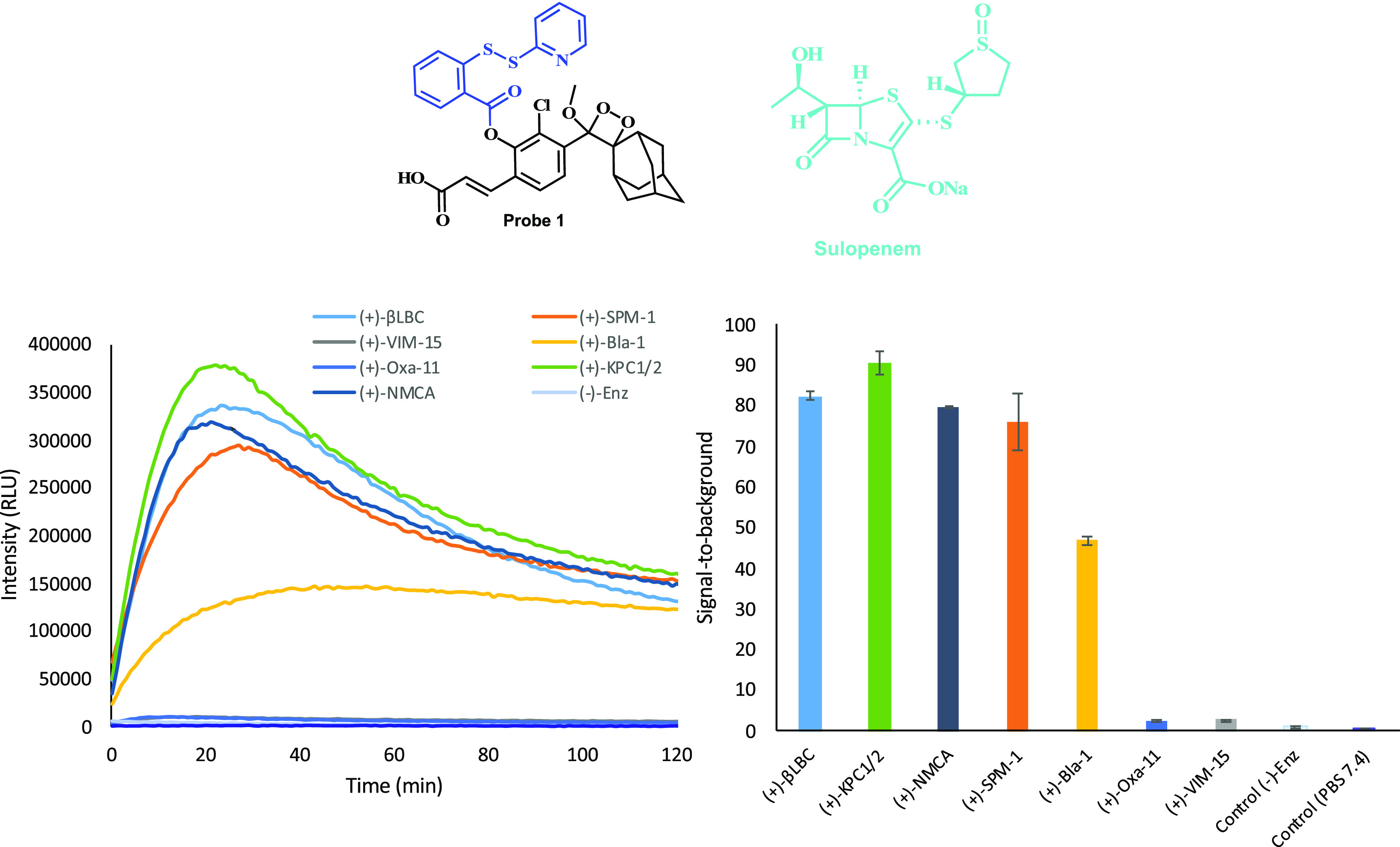
Chemiluminescence kinetic profiles [left] and total light emission [right] (over 2 h) produced by probe **1** [25 μM] in PBS (pH 7.4) at 27 °C and seven different β-lactamases [**βLBC** = 10 U/mL, other enzymes 2 U/mL] in the presence of sulopenem [1 mM]. Probe **1** was preincubated for 45 min in PBS (pH 7.4) prior to use; sulopenem with enzymes were incubated for 30 min prior to measurement.

We next sought to compare the ability of probe **1** to detect hydrogen sulfide release from a β-lactam using several different β-lactamases. The β-lactam antibiotic sulopenem was selected for this comparison experiment, as its molecular structure contains three atoms of sulfur, and since it produces a rapid response rate in the initial time slot of the measurement. Seven different β-lactamases were incubated with sulopenem in the presence of probe **1** in PBS 7.4, and the light emission kinetic profile produced by the probe was measured for 2 h (Figure 5). Five out of the seven β-lactamases tested in this assay (**βLBC**, **KPC1/2**, **NMCA**, **SPM-1**, **Bla-1**) presented a significant turn-on response of probe **1** in the presence of sulopenem. The β-lactamases, **Oxa-11** and **VIM-15**, failed to show substantial light emission signal. Importantly, control measurement of probe **1** with sulopenem in the absence of any β-lactamase has resulted in only negligible light emission signal.

**Figure 6:**
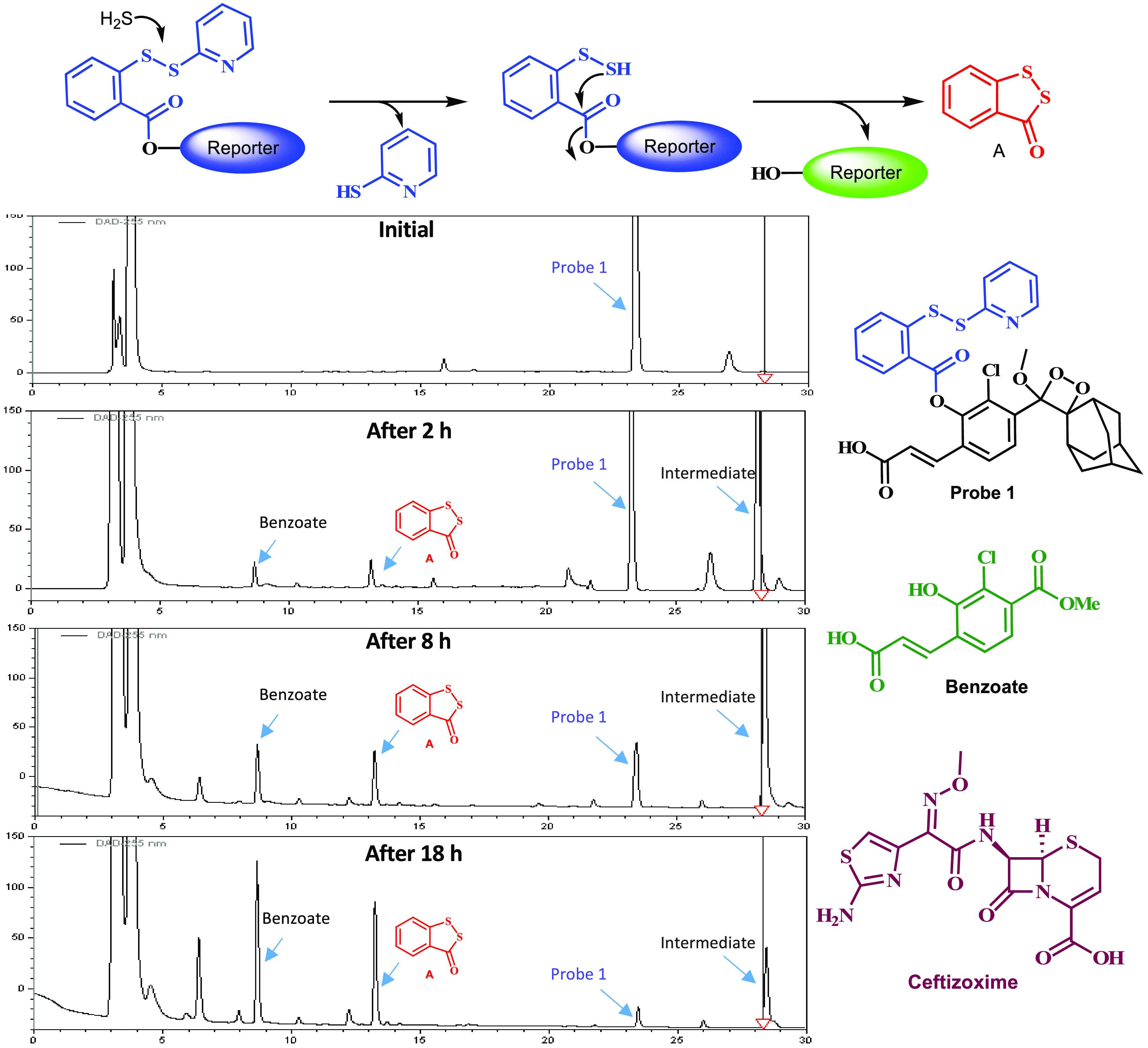
RP-HPLC chromatograms showing gradual formation of byproduct **A** upon decomposition of probe **1** [100 μM] in the presence of ceftizoxime [2 mM] and **βLBC** [20 U/mL] in PBS (pH 7.4) at RT. HPLC elution gradient ACN and water with 0.1 TFA (30–100%).

Examination of the mechanism in which hydrogen sulfide reacts with the S–S responsive group of probe **1** reveals that byproduct **A** should be formed (see Figure 6). To provide additional support for the release of hydrogen sulfide from β-lactam antibiotics upon their biodegradation by β-lactamase, we monitored the progress of the ceftizoxime biodegradation in the presence of probe **1** by RP-HPLC and checked whether a formation of byproduct **A** could be detected. Figure 6 clearly shows that probe **1** is decomposed to gradually generate byproduct **A** and a benzoate over time, upon **βLBA**-catalyzed hydrolysis of ceftizoxime. Control measurement in the absence of the β-lactamase has revealed no formation of byproduct **A** at all. A similar biodegradation pattern was observed for the β-lactam cefalexin, upon incubation with **βLBA** and probe **1** (see Supporting Information, Figure S4). This observation provides direct evidence for the release of hydrogen sulfide from cefalexin biodegradation.

**Figure 7:**
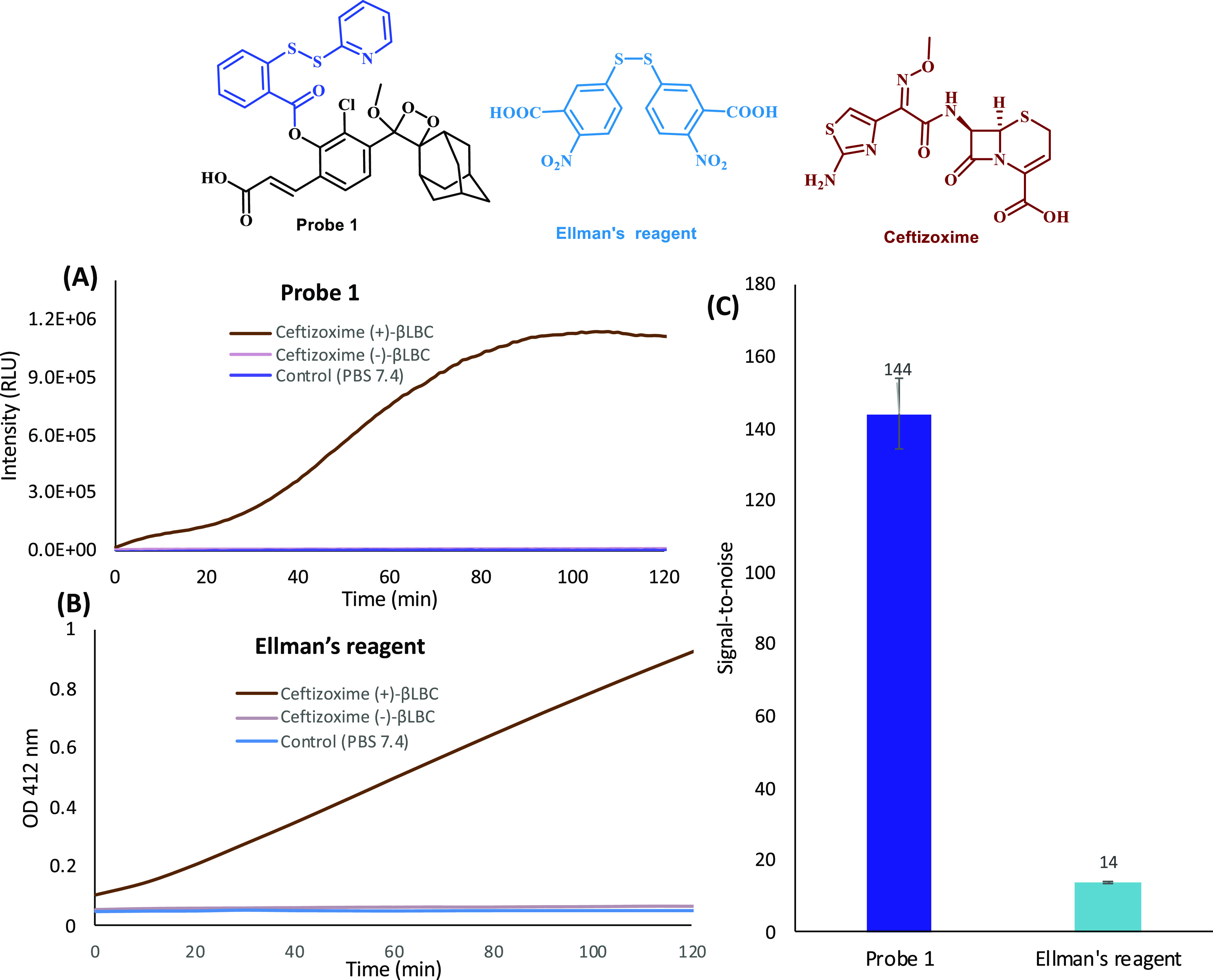
Comparison of kinetic profiles (part A for probe **1** and part B for Ellman’s reagent) and signal-to-background (part C, after 2 h) of chemiluminescence probe 1 [25 μM] and Ellman’s reagent [125 μM] in PBS (pH 7.4) at 27 °C for detection of H_2_S released by ceftizoxime [1 mM ] in the presence of **βLBC** (10 U/mL). Probe **1** and Ellman’s reagent were preincubated for 45 min in PBS (pH 7.4) prior to use; ceftizoxime with enzymes was incubated for 30 min prior to measurement.

A common reagent for detection of organic molecules with a general thiol functional group (RSH), including hydrogen sulfide, is the Ellman’s reagent, which forms a yellow color upon reaction with such compound in aqueous solution. To demonstrate the high detection sensitivity of our chemiluminescence probe **1** for hydrogen sulfide, we performed a simple comparison measurement with the commercially available reagent. Probe **1** and the Ellman’s reagent were incubated with ceftizoxime and **βLBC**, and the light emission signal (for probe **1**) or the absorbance signal (for Ellman’s reagent) were monitored over 2 h (Figure 7). Under these conditions, chemiluminescence probe **1** was able to provide a 10-fold greater detection sensitivity than the Ellman’s reagent with a S/N value of 144.

**Figure 8:**
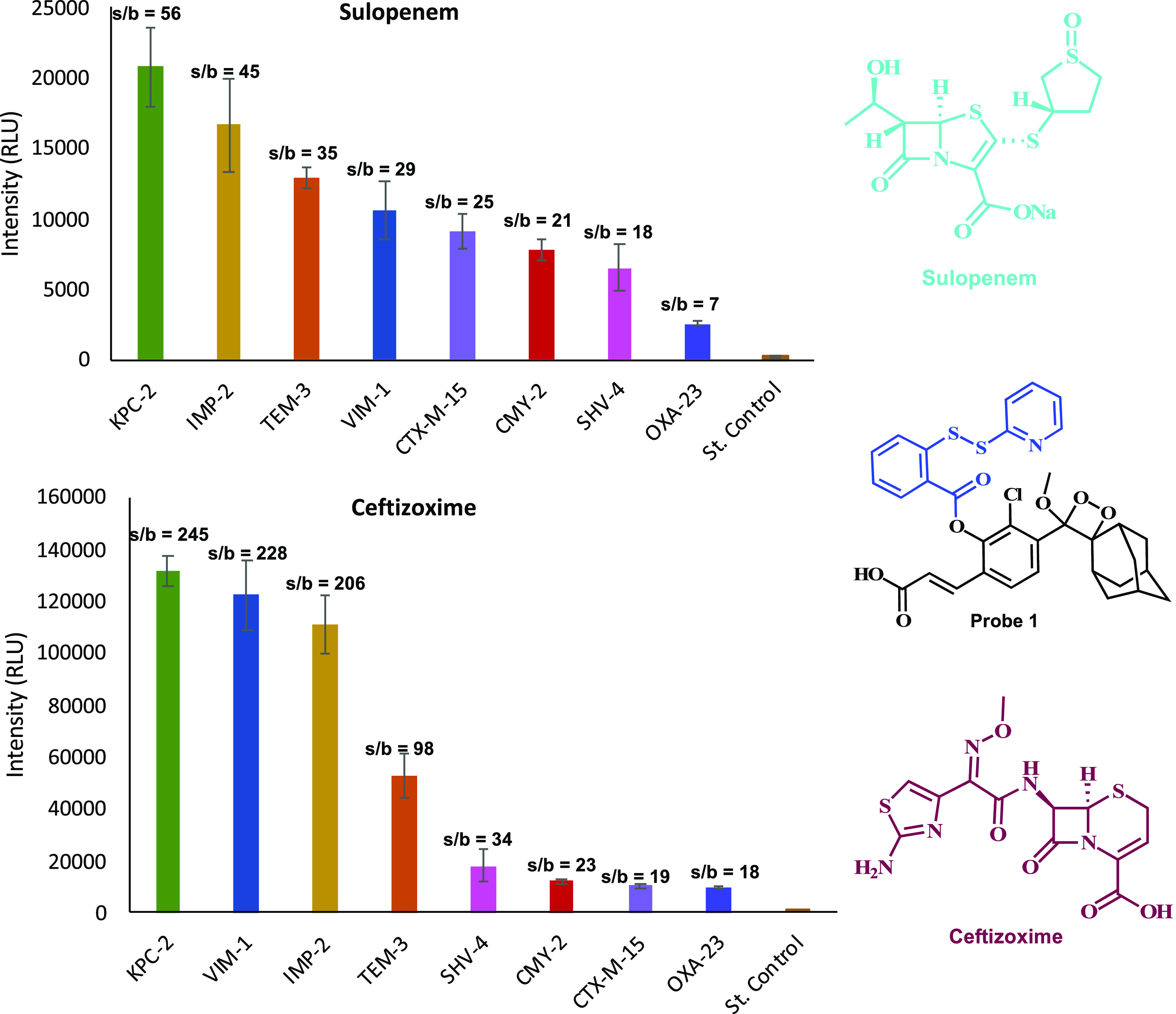
Detection of antibiotic-resistant bacteria [100 μL of cell suspension OD_600_ 1.0] after 60 min with sulopenem (up) [1 mM] or ceftizoxime (down) [1 mM] in the presence of probe **1** [25 μM]; s/b = signal-to-background ratio where the background is the signal value obtained for the sterile control.

Encouraged by the data obtained so far, we sought to assess whether probe **1** can detect hydrogen sulfide in a functional bacterial assay using antibiotic-resistant bacterial strains and β-lactam antibiotics. Briefly, the β-lactams ceftizoxime or sulopenem were preincubated for 30 min at room temperature with eight different antibiotic-resistant bacterial strains. Then, probe **1** was added and its light emission signal was measured for 1 h at room temperature using a plate reader. The obtained results are presented in Figure 8. Successfully, all eight β-lactam antibiotic-resistant bacteria strains exhibited substantial signal-to-background value, varied between 7-fold and 14-fold for sulopenem and between 18-fold and 245-fold for ceftizoxime. Only a slight amount of light emission signal was observed for the sterile control.

**Figure 9:**
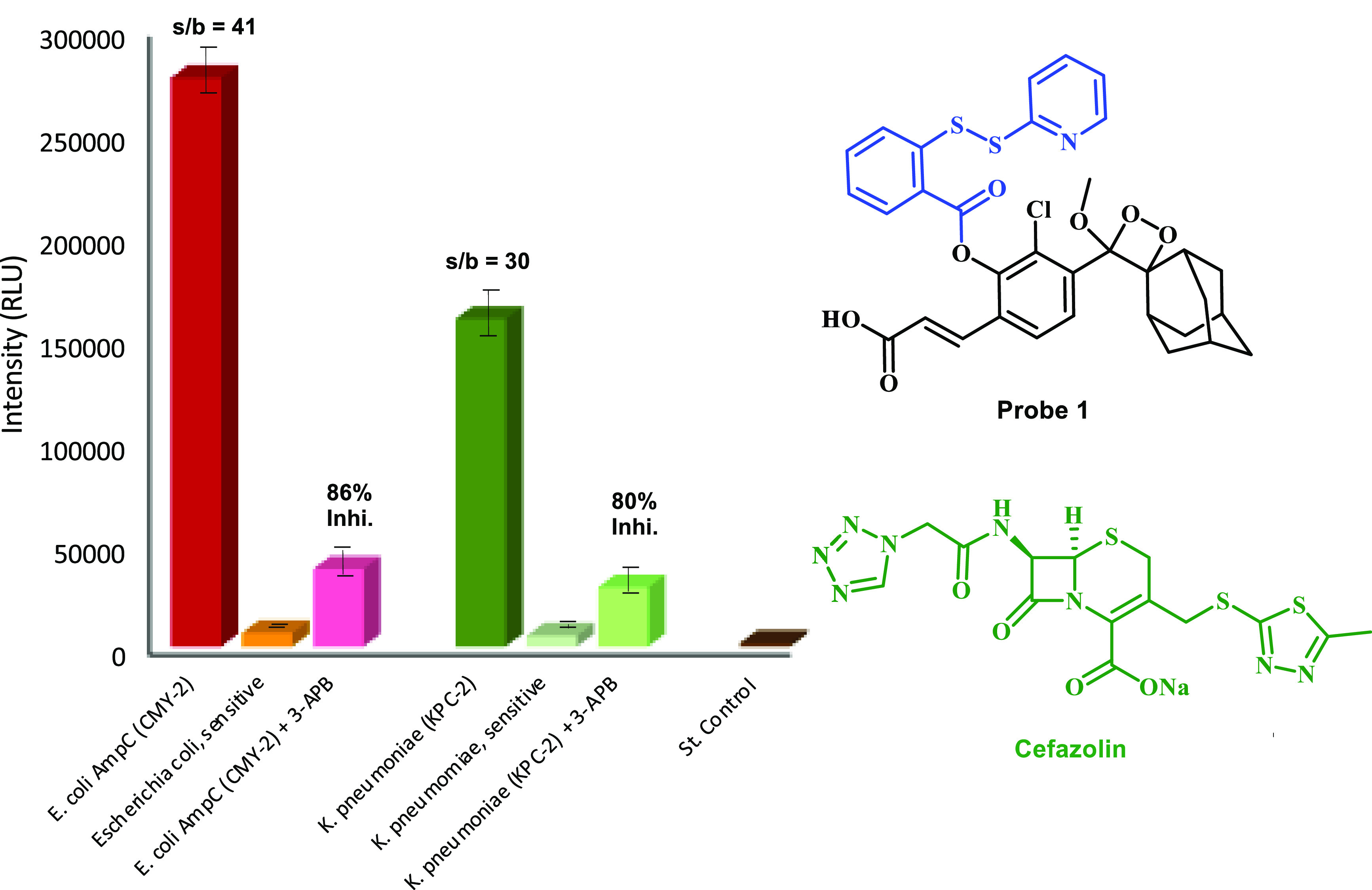
Detection of cephalosporinase-positive bacteria [100 μL of cell suspension OD_600_ 1.0] after 15 min in one-step assay with cefazolin [1 mM] and probe **1** [25 μM] and the effect of 3-aminophenyl boronic acid [7 mM] on β-lactamase activity; s/b = signal-to-background ratio, where the background is the signal value obtained by the sensitive strain.

We next sought to further evaluate the feasibility of probe **1** to differentiate between a bacterial β-lactam resistant strain and β-lactam sensitive strain that had initially been isolated from patients. Briefly, the β-lactam cefazolin was incubated with two strains expressing serine-type β-lactamases, *Klebsiella pneumonia* RKI 92/08 (KPC-2) and *Escherichia coli* RKI 66/09 (CMY-2), and two antibiotic-sensitive reference strains, *Klebsiella pneumonia* RKI 2867/81 and *Escherichia coli* ATCC 25922. In addition, the serine β-lactamase inhibitor 3-aminophenylboronic acid was added to a subset of wells. Immediately after probe **1** was added, the light emission signal was monitored at room temperature for 15 min, using a plate reader. The obtained results are presented in Figure 9.

To our delight, the two strains expressing β-lactamases, *Klebsiella pneumonia* RKI 92/08 (KPC-2) and *Escherichia coli* RKI 66/09 (CMY-2), exhibited strong light emission signal, in comparison to the signal produced by their counterpart sensitive strains, *Klebsiella pneumonia* RKI 2867/81 and *Escherichia coli* ATCC 25922, with a signal-to-background ratio value of 30 and 41, respectively. Addition of the the β-lactamase inhibitor 3-aminophenylboronic acid to the bacterial strains expressing β-lactamases resulted in significant (80% and 86%) reduction of the emitted light signal. These data suggest that probe **1** can be effectively applied to identify if a certain bacterial strain possesses resistance to a specific β-lactam antibiotic.

Hydrogen sulfide is an important biological signaling molecule that recently is gaining considerable scientific attention.^25−27^ Therefore, numerous probes, including a chemiluminescent one,^28^ for detection of hydrogen sulfide were developed in order to better understand the chemistry and properties of this signaling molecule in biological systems.^23,24,29,30^ However, hydrogen sulfide was never linked to β-lactamase activity and never considered as a biodegradation marker for antibiotic bacterial resistance.

It should be noted that in addition to hydrogen sulfide, some alkyl-thiols (RSH) can also be released during β-lactam antibiotic biodegradation. Thus, a probe designed to detect general alkyl-thiols, may be able to act more efficiently for such assay. Yet, in the preliminary screening studies described in Figure 2, probe **3**, which can detect ubiquitous thiols, including general alkyl-thiols and hydrogen sulfide, produces significantly lower detection sensitivity than that observed by probe **1** (a selective probe for detection of hydrogen sulfide). Unexpectedly, probe **2**, which is known to be more selective toward hydrogen sulfide^31^ than probe **1**, did not exhibit better detection efficiency in our assay. We speculate that a possible dual activation mechanism by both hydrogen sulfide and general alkyl-thiols contributes to the superior detection sensitivity obtained with probe **1**.

Direct detection of β-lactamase catalytic activity by colorimetric turn-on probes is a common technique for determination of bacterial resistance. Only a few modified β-lactam compounds are commercially available for such a detection mode; one example is nitrocefin, that changes its color from yellow to red upon hydrolysis.^10^ Unlike a color change obtained for nitrocefin, probe **1** produces a chemiluminescent diagnostic signal, which results in significantly higher detection sensitivity.^32−34^ Our approach for monitoring β-lactam antibiotic resistance is singular, since it is the only method for which its mode-of-action is based on detection of a degradation product commonly released from a broad spectrum of β-lactam antibiotic molecules. This β-lactam degradation product is generated as a result of the β-lactamase catalytic activity, which is the cause for the bacterial resistance. Therefore, detection of hydrogen sulfide release from β-lactam-containing sulfur by β-lactamase catalytic activity can directly indicate bacterial resistance for the specific examined antibiotic. The relatively high antibiotic concentration (1 mM) used in our assay to monitor hydrogen sulfide release is not producing any limitation, since the assay is performed for a short time in a test tube setup. It should be noted that bacteria often have pathways that generate intrinsic hydrogen sulfide, so it is important to include the appropriate negative control experiments to account for background bacterial hydrogen sulfide production.

The different kinetic profile behaviors of the five β-lactam antibiotics, which were successfully tested with probe **1** in our assay (Figure 4), indicate various degradation pathways for release of hydrogen sulfide. However, some β-lactam antibiotics (Figure 3B) failed to produce a release of hydrogen sulfide, most likely due to different subsequent chemical degradation pathways in aqueous solution, which lead to the formation of RSH groups and eventually the formation of H_2_S. To provide a plausible explanation for the observed biodegradation behavior for the various β-lactam antibiotics, we performed a comprehensive HPLC and MS analysis study (see Supporting Information). The data obtained in this study provide some experimental support for the proposed different degradation pathways observed for the β-lactam antibiotics of group A and group B, shown in Figure 3. For example, degradation of β-lactams like ceftizoxime, faropenem, and sulopenem results in formation of unstable RSH functional groups that eventually lead to generation H_2_S. On the other hand, degradation of the β-lactam structure existing for cefotaxime, ceftazidime, ceftriaxone, or imipenem, results in no formation of RSH functional groups and thus also no H_2_S release. While the obtained data rationalized some justification for different degradation patterns of β-lactams, further experimental work will be needed to fully elucidate the difference in the degradation behavior.^35,36^ Overall, the assay developed in this work for detection of hydrogen sulfide release for β-lactam biodegradation by β-lactamase was successfully implemented for the five β-lactams: sulopenem, faropenem, cefalexin, ceftizoxime, and cefazolin.

## Conclusions

In summary, we have evaluated a new, distinct diagnostic technique for detection of β-lactam antibiotic resistance in bacteria. The technique employed a highly efficient chemiluminescence probe designed for detection of hydrogen sulfide in aqueous environment. Evidentially, hydrogen sulfide is a metabolite, which is generated upon biodegradation of β-lactam antibiotics by β-lactamases. As far as we know, this is the first diagnostic assay for detection of antibiotic resistance, based on a metabolite, common to several β-lactams, that is generated as a result of the bacterial resistance mechanism. Such assay can directly indicate if antibiotic bacterial resistance exists for a certain examined β-lactam. The assay was successfully demonstrated with five different β-lactam antibiotics and eight bacterial resistant strains. Most importantly, while testing this technique in a functional bacterial assay, the chemiluminescence probe was able to clearly distinguish between a β-lactam resistant bacterial strain and a sensitive one. Bearing in mind the data obtained in this study, we propose that hydrogen sulfide may be considered as an emerging β-lactam metabolite for detection of bacterial resistance.

## Supporting Information Available

Synthetic procedures, characterization data (NMR and MS) for all new compounds, and chemiluminescence control experiments (PDF)
