# Supplementary material for: Chemiluminescence Detection of Hydrogen Sulfide Release by β-Lactamase-Catalyzed β-Lactam Biodegradation: Unprecedented Pathway for Monitoring β-Lactam Antibiotic Bacterial Resistance
Source: Bioconjug Chem. 2021 Apr 25;32(5):991–1000. doi: 10.1021/acs.bioconjchem.1c00149 (PMC8382227; doi:10.1021/acs.bioconjchem.1c00149)
Supplement: Supplementary file 1 — bc1c00149_si_001.pdf [file bc1c00149_si_001.pdf]

Chemiluminescence Detection of Hydrogen Sulfide Release by *β*-Lactamase catalyzed *β*-Lactam Biodegradation: Unprecedented Pathway for Monitoring *β*-Lactam Antibiotic Bacterial Resistance

Sachin Popat Gholap[a], Chunyan Yao[b], Ori Green[a], Matej Babjak[c], Pavol Jakubec[d], Tomáš Malatinský[d] Julian Ihssen[b], Lukas Wick[b], Urs Spitz[b*], and Doron Shabat[a*]

[a]School of Chemistry, Raymond and Beverly Sackler Faculty of Exact Sciences, Tel Aviv University, Tel Aviv 69978 Israel. [b]BIOSYNTH CARBOSYNTH, Rietlistr. 4 Postfach 125 9422 Staad, Switzerland. [c]Department of Organic Chemistry, Faculty of Chemical and Food Technology, Slovak University of Technology in Bratislava, Radlinského 9, 81237 Bratislava, Slovakia. [d]Auchem s.r.o., A. Hlinku 1452/3, 022 01 Čadca, Slovakia.

Supporting Information

Sr. No.	Topic	Page no.
1	General information	02
2	Synthetic schemes and experimental procedures	03
3	Spectroscopic data	11
3a	Evaluation of the selectivity of probes 1-3 towards H_2S and RSH	11
3b	Determination of the Limit of Detection (LOD) of probe **1** for detection of H_2S	12
3c	Detection of H_2S-released from antibiotics degradation by βLBC	13
3d	Formation of byproduct A upon biodegradation of probe **1** in the presence of ceftizoxime or cefalexin with βLBC	15
3e	General procedure for the data given in **Figure 8** (Detection of antibiotic resistant bacteria using probe **1**)	16
3f	Detection of carbapenem-resistant bacteria with Faropenem and probe **3**	17
3g	General procedure for the data given in **Figure 9**	18
3h	Determination of the Limit of Detection (LOD) of probe **1** for the detection of antibiotic resistant bacteria using Ceftizoxime	19
3i	Determination of the relative rate constant of the reaction of hydrogen sulfide release by β-lactamase-catalyzed β -lactam biodegradation of various antibiotics	20
4	NMR and MS Spectra	22
5	References	42
6	Appendix	43

1. General Information

All reactions requiring anhydrous conditions were performed under an Argon atmosphere. All reactions were carried out at room temperature unless stated otherwise. Chemicals and solvents were either A.R. grade or purified by standard techniques. Thin-layer chromatography (TLC): silica gel plates Merck 60 F254: compounds were visualized by irradiation with UV light. Column chromatography (FC): silica gel Merck 60 (particle size 0.040-0.063 mm), eluent given in parentheses. Reverse-phase high-pressure liquid chromatography (RP-HPLC): C18 5u, 250x4.6mm, eluent given in parentheses. Preparative RP-HPLC: C18 5u, 250x21mm, eluent given in parentheses. ^{1}H-NMR spectra were measured using Bruker Avance operated at 300, 400 and 600 MHz. ^{13}C-NMR spectra were measured using Bruker Avance operated at 101 and 151 MHz. Chemical shifts were reported in ppm on the δ scale relative to a residual solvent ($CDCl_3$: δ = 7.26 for ^{1}H-NMR and 77.16 for ^{13}C-NMR, DMSO-d_6: δ = 2.50 for ^{1}H-NMR and 39.52 for ^{13}C-NMR). Mass spectra were measured on Waters Xevo TQD. Chemiluminescence was recorded on Molecular Devices Spectramax i3x and SpectraMax M5 plate reader. Fluorescence was recorded on Tecan infinite 200 Pro. All general reagents, including salts and solvents, were purchased from Sigma-Aldrich. Light irradiation for photochemical reactions: LED PAR38 lamp (19W, 3000K). *β*-Lactamase, *Bacillus cereus* 569/H9 (***βLBC***) was purchased from Merck Millipore; all other *β*-lactamases were obtained from Biosynth-Carbosynth.

Abbreviations

ACN - Acetonitrile, **CDI** - 1,1'-Carbonyldiimidazole, **DCC** - *N*, *N'*-Dicyclohexylcarbodiimide, **DCM** - dichloromethane, **DIPEA** - *N*,*N*-Diisopropylethylamine, **DMF** - *N*,*N'*-Dimethylformamide, **DMBA** - Dimethylbarbituric acid, **DMAP** - 4-(Dimethylamino)-pyridine, **DMSO** - Dimethyl sulfoxide, **EDC** - 1-Ethyl-3-(3-dimethylaminopropyl)-carbodiimide, **EEDQ** - N-Ethoxycarbonyl-2-ethoxy-1,2-dihydroquinoline, **EtOAc** - Ethylacetate, **HBTU** - 2-(1H-Benzotriazol-1-yl)-1,1,3,3-tetramethyluronium hexafluorophosphate, **Hex**- Hexane, **TFA** - Trifluoroacetic acid, **TEA** - Triethylamine, **THF** - Tetrahydrofuran, **TMSCl** - Trimethylsilyl chloride, **PBS** - Phosphate-buffered saline.

2. Synthetic Schemes and Experimental Procedures

Scheme 1. Synthesis of probe 1.

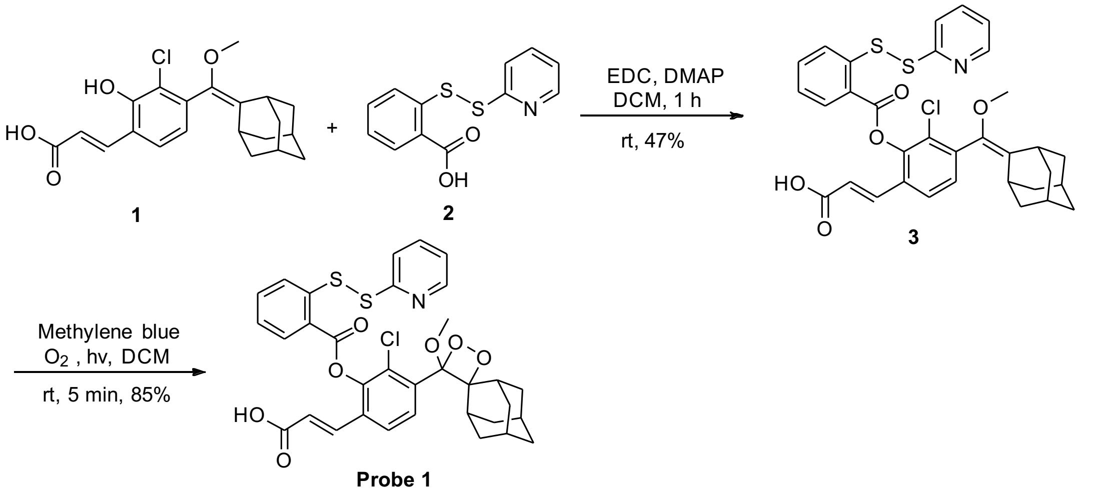

Compound 3:

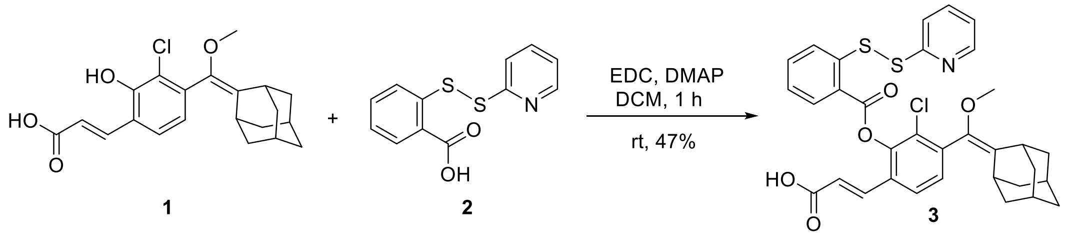

Compound **1**[1] (50 mg, 0.13 mmol, 1 eq), compound **2**[2] (70 mg, 0.26 mmol, 2 eq) and 4-DMAP (33 mg. 0.26 mmol, 2 eq) were dissolved in DCM (4 mL). Then EDC (21 mg, 0.13 mmol, 1 eq) was added and the reaction mixture was stirred for 30 minutes at room temperature. After consumption of compound **1** (as indicated by TLC) the reaction was diluted with EtOAc (30 mL), washed with 1M HCl (10 mL), dried over Na_2SO_4 and concentrated under reduced pressure. The crude mixture was purified using silica column chromatography to elute pure **3** as a white solid (39 mg, 47%). ^{1}H NMR (400 MHz, $CDCl_3$) δ 8.50 (d, *J* = 4.2 Hz, 1H), 8.42 (dd, *J* = 7.8, 1.3 Hz, 1H), 7.99 (d, *J* = 8.1 Hz, 1H), 7.82 (d, *J* = 16.1 Hz, 1H), 7.57-7.67 (m, 4H), 7.38 (t, *J* = 7.3 Hz, 1H), 7.29 (t, *J* = 8.1 Hz, 1H), 7.14-7.11 (m, 1H), 6.52 (d, *J* = 15.9 Hz, 1H), 3.36 (s, 3H), 3.29 (s, 1H), 2.16 (s, 1H), 2.00-1.72 (m, 12H). ^{13}C NMR (101 MHz, $CDCl_3$) δ 170.4, 163.6, 158.9, 149.0, 146.4, 141.8, 139.3, 139.1, 138.6, 138.4, 134.5, 132.6, 130.0, 129.3, 129.0, 126.5, 126.4, 125.5, 125.3, 121.5, 121.0, 120.5, 57.6, 39.2, 38.8, 37.2, 33.1, 29.9, 28.3. MS (ES+): m/z calc. for $C_{33}H_{30}ClNO_5S_2$: 619.1; found: 620.5 $[M + 1]^+$.

Probe 1:

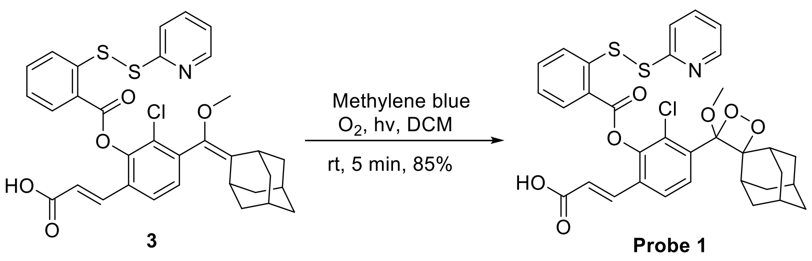

Enol-ether **3** (30 mg, 0.048 mmol, 1 eq) and catalytic amount (~2 mg) of methylene blue were dissolved in 10 mL of DCM. Oxygen was bubbled through the solution while irradiating with yellow light for 5 minutes. The reaction progress was monitored by RP-HPLC. Upon completion, solvent was removed and the crude mixture was then purified by preparative RP-HPLC [70-100% ACN in water (0.1 % TFA), 20 min] to afford **probe 1** as white solid (26.8 mg, 85%). ^{1}H NMR (400 MHz, DMSO-d_6) δ 8.44-8.50 (m, 2H), 8.14 (d, J = 8.6 Hz, 1H), 8.00 (dd, J = 8.3, 1 Hz, 1H), 7.74-7.83 (m, 2H), 7.50-7.56 (m, 3H), 7.25-7.30 (m, 1H), 6.75 (d, J = 16 Hz, 1H), 3.15 (s, 3H), 2.88 (s, 1H), 2.16 (m, 1H), 1.95 (s, 1H), 1.30-1.78 (m, 12H). ^{13}C NMR (101 MHz, DMSO-d_6) δ 167.3, 163.7, 157.4, 150.5, 146.5, 141.4, 138.8, 135.9, 135.5, 134.7, 133.0, 131.5, 131.20, 130.23, 127.6, 126.9, 126.7, 126.4, 125.4, 124.9, 122.5, 120.4, 111.6, 96.1, 50.1, 36.4, 33.9, 33.7, 32.3, 31.7, 31.5, 26.1, 25.8. MS (ES+): m/z calc. for $C_{33}H_{30}ClNO_7S_2$: 651.1; found: 652.5 $[M + 1]^+$.

Scheme 2. Synthesis of probe 2.

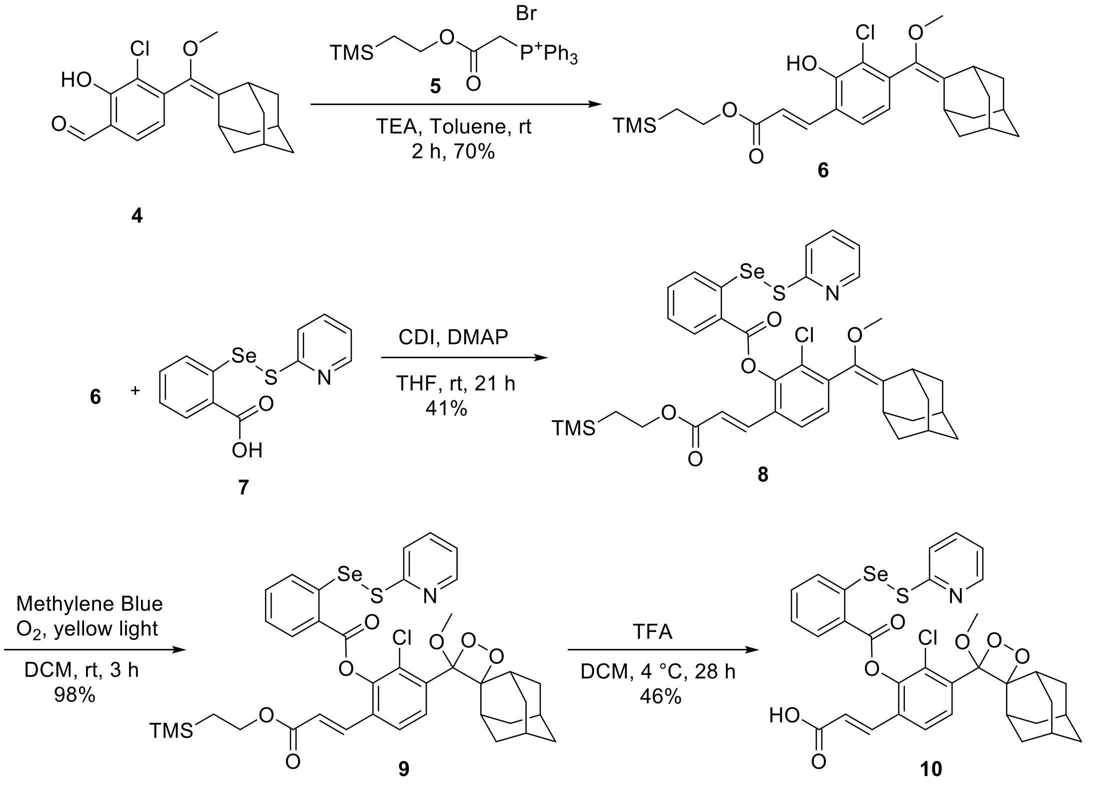

Compound 6:

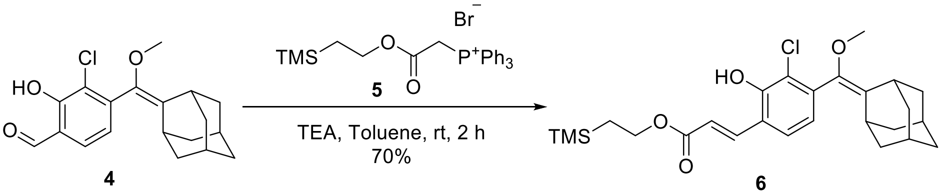

Aldehyde **4**[3] (0.700 g, 2.1 mmol, 1 eq) and phosphonium salt **5**[4] (1.160 g, 2.3 mmol, 1.1 eq) were dissolved in dry toluene (14 mL) under argon atmosphere. TEA (0.35 mL, 2.5 mmol, 1.2 eq) was added and the reaction mixture was stirred for 2 h at room temperature. Upon completion, the reaction mixture was diluted with Et_2O (25 mL) and the organic layer was washed with sat. NH_4Cl (20 mL). The separated aqueous layer was extracted one more time with Et_2O (25 mL). The combined organic layers were dried with $MgSO_4$, filtered and concentrated *in vacuo*. The crude product was purified using silica column chromatography to elute compound **6** as a white solid (0.700 g, 70%). ^{1}H NMR (300 MHz, $CDCl_3$) δ 7.93 (d, *J* = 16.2 Hz, 1H), 7.38 (d, *J* = 8.0 Hz, 1H), 6.86 (d, *J* = 8.0 Hz, 1H), 6.59 (d, *J* = 16.2 Hz, 1H), 6.27 (s, 1H), 4.32 (t, *J* = 8.4 Hz, 2H), 3.31 (s, *J* = 7.2 Hz, 3H), 3.27 (s, 1H), 2.12 (s, 1H), 1.96-1.58 (m, 12H), 1.08 (t, *J* = 8.4 Hz, 2H), 0.08 (s, 9H). ^{13}C NMR (151 MHz, $CDCl_3$) δ 167.4, 150.7, 139.6, 138.8, 136.5, 132.9, 126.8, 123.7, 122.2, 121.6, 120.5, 62.9, 57.4, 39.2, 38.9, 37.2, 33.0, 29.9, 28.5, 17.5, -1.3. MS (ES+): m/z calc. for $C_{26}H_{35}ClNO_4Si$: 474.2; found: 475.2 $[M + 1]^{+\cdot}$

Compound 8:

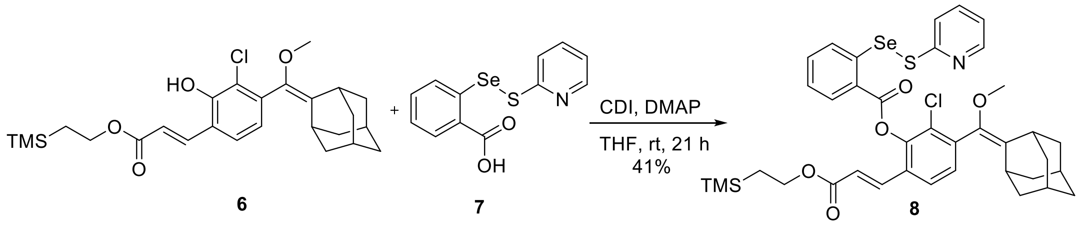

Compound **7**[5] (340 mg, 1.1 mmol, 1.3 eq) and CDI (191 mg, 1.18 mmol, 1.4 eq) were dissolved in dry THF (8.4 mL) under argon atmosphere and stirred for 1 h at room temperature. Then, compound **6** (400 mg, 0.84 mmol, 1 eq) followed by DMAP (10 mg, 0.08 mmol, 0.1 eq) were added and the stirring was continued for an additional 21 h at room temperature. After completion, the solid was filtered off and washed with Et_2O (20 mL) and filtrate was concentrated under reduced pressure. The crude product was purified using silica column chromatography to elute compound **8** as white solid (0.264 g, 41%). ^{1}H NMR (300 MHz, $CDCl_3$) δ 8.50-8.40 (m, 2H), 8.12 (dd, *J* = 8.1, 0.7 Hz, 1H), 7.72 (d, *J* = 16.1 Hz, 1H), 7.63-7.53 (m, 4H), 7.43 (td, *J* = 7.8, 1.0 Hz, 1H), 7.28 (d, *J* = 8.3 Hz, 1H), 7.05 (ddd, *J* = 6.7, 4.9, 1.7 Hz, 1H), 6.50 (d, *J* = 16.0 Hz, 1H), 4.26 (t, *J* = 8.4 Hz, 2H), 3.36 (s, 3H), 3.29 (s, 1H), 2.17 (s, 1H), 1.97-1.58 (m, 12H), 1.00 (t, *J* = 8.4 Hz, 2H), 0.02 (s, *J* = 3.2 Hz, 9H). ^{13}C NMR (151 MHz, $CDCl_3$) δ 166.5,

165.0, 158.6, 149.7, 146.1, 139.5, 139.1, 138.1, 137.5, 136.8, 134.7, 132.5, 130.0, 129.3, 129.2, 128.2, 126.7, 125.8, 125.1, 122.3, 121.8, 120.8, 63.2, 57.6, 39.4, 39.2, 38.7, 37.2, 33.1, 29.9, 29.8, 28.5, 28.3, 17.4, -1.3. MS (ES+): m/z calc. for $C_{38}H_{42}ClNO_5SSeS$: 767.1; found: 768.1 $[M + 1]^+$.

Compound 9:

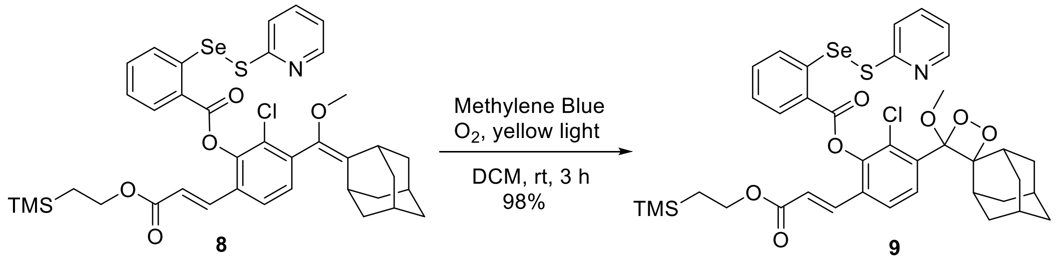

Compound **8** (80 mg, 0.1 mmol, 1 eq) was dissolved in DCM (10 mL). Methylene blue (3.3 mg, 0.01 mmol, 0.1 eq) was added to reaction mixture and the final blue solution was stirred under O_2 atmosphere (balloon) while irradiating with yellow light for 3 h. Upon completion, the crude mixture was eluted with Et_2O on a short silica gel column and the desired product **9** (82 mg, 98%) was obtained as a pale pink foam. ^{1}H NMR (600 MHz, $CDCl_3$) δ 8.49-8.41 (m, 2H), 8.14-8.09 (m, 2H), 7.77-7.69 (m, 2H), 7.61-7.55 (m, 3H), 7.43 (t, *J* = 7.3 Hz, 1H), 7.06 (ddd, *J* = 6.6, 4.9, 1.4 Hz, 1H), 6.56 (d, *J* = 16.0 Hz, 1H), 4.27 (t, *J* = 8.4 Hz, 2H), 3.26 (s, 3H), 3.02 (s, 1H), 2.24 (d, *J* = 2.2 Hz, 1H), 2.0. (s, 1H), 1.89-1.32 (m, 11H), 1.00 (t, *J* = 8.4 Hz, 2H), 0.01 (s, 9H). ^{13}C NMR (151 MHz, $CDCl_3$) δ 166.3, 165.0, 158.4, 149.7, 139.6, 137.4, 136.3, 135.3, 134.8, 132.4, 131.2, 128.3, 126.7, 125.6, 125.3, 123.5, 121.9, 120.9, 111.7, 63.3, 50.0, 39.4, 36.7, 34.1, 33.8, 32.4, 31.8, 31.7, 29.8, 26.3, 25.9, 17.4, -1.3. MS (ES+): m/z calc. for $C_{38}H_{42}ClNO_7SSeSi$: 799.1; found: 800.1 $[M + 1]^+$.

Probe 2:

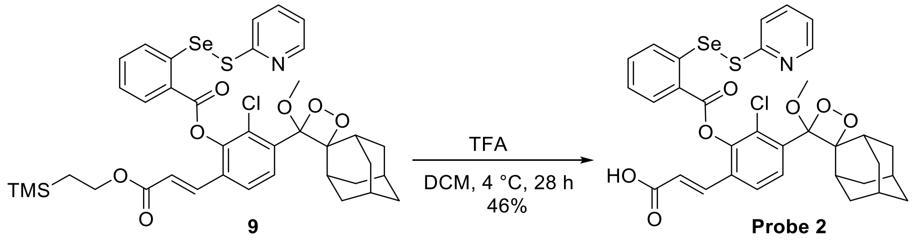

Compound **9** (70 mg, 0.09 mmol, 1 eq) was dissolved in dry DCM (4 mL) and cooled to 0 °C. TFA (0.5 mL, 6.53 mmol) was added and the final solution was allowed to stay in fridge (4 °C) for 28 h (HPLC conversion-**probe 2:9** = 70:30). The crude reaction mixture was concentrated and purified by preparative RP-HPLC (isocrat CH_3CN/30 mM NH_4HCO_3 = 6:4) to afford **probe 2** as an off-white solid (28 mg, 46%). ^{1}H NMR (600 MHz, CD_3OD) δ 8.50 (d, *J* = 7.6 Hz, 1H), 8.36 (d, *J* = 4.0 Hz, 1H), 8.07 (t, *J* = 8.9

Hz, 2H), 7.91 (d, J = 8.4 Hz, 1H), 7.71-7.60 (m, 3H), 7.55-7.47 (m, 2H), 7.16 (dd, J = 6.7, 5.1 Hz, 1H), 6.68 (d, J = 16.0 Hz, 1H), 3.22 (s, 3H), 2.95 (s, 1H), 2.29 (d, J = 12.1 Hz, 1H), 2.03 (s, 1H), 1.89-1.37 (m, 12H). ^{13}C NMR (151 MHz, CD_3OD) δ 171.5, 165.1, 157.7, 148.9, 138.3, 138.0, 134.5, 133.8, 132.4, 132.3, 131.9, 130.6, 127.3, 126.6, 126.2, 125.0, 122.0, 121.1, 111.3, 48.6, 33.6, 33.6, 31.8, 31.5, 31.3, 26.3, 25.9. MS (ES+): m/z calc. for $C_{33}H_{30}ClNO_7SSe$: 699.1; found: 700.1 $[M + 1]^+$.

Scheme 3. Synthesis of probe 3.

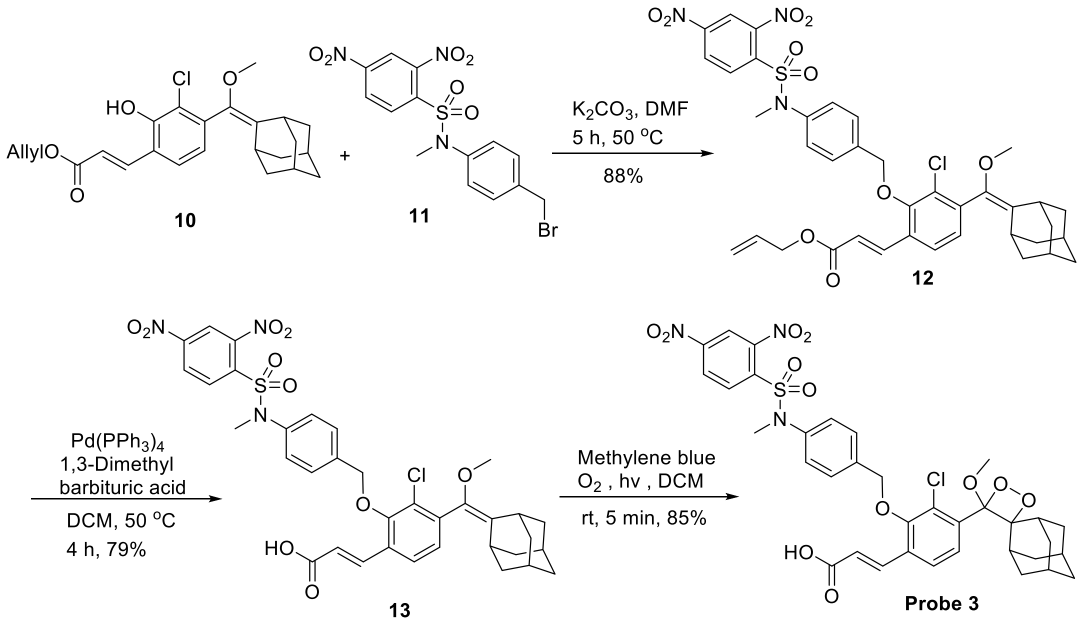

Compound 12:

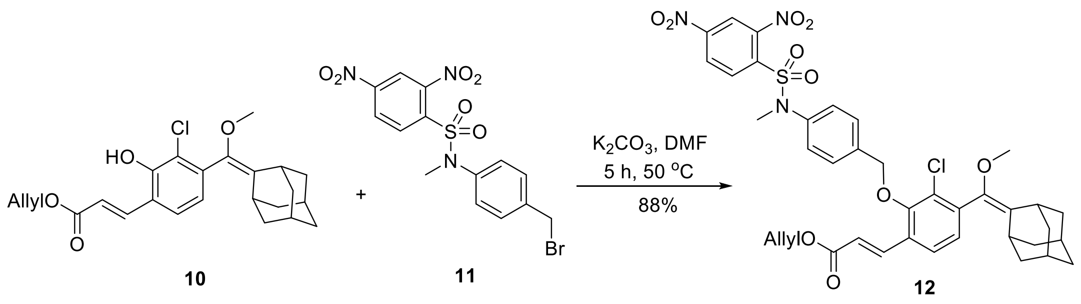

Compound **10**[3] (200 mg, 0.48 mmol, 1 eq) was dissolved in DMF (2 mL) under argon. K_2CO_3 (0.100 mg, 0.724 mmol, 1.5) was added followed by compound **11**[6] (228 mg, 0.531 mmol, 1.1 eq) and the final solution was heated to 50 °C for 5 h. After starting material (compound **10**) was consumed as indicated by TLC, the reaction was diluted with EtOAc (30 mL), washed with water (20 mL), dried over Na_2SO_4 and concentrated under reduced pressure. The crude product was then purified using silica column chromatography to elute **12** as a white solid (0.324 g, 88%). ^{1}H NMR (400 MHz, $CDCl_3$) δ 8.45 (d, J = 2.2 Hz, 1H), 8.36 (dd, J = 8.7, 2.2 Hz, 1H), 7.94 (d, J = 16.2 Hz,

1H), 7.74 (d, J = 8.7 Hz, 1H), 7.50 (dd, J = 14.3, 8.2 Hz, 3H), 7.31-7.23 (m, 2H), 7.11 (d, J = 8.0 Hz, 1H), 6.50 (d, J = 16.1 Hz, 1H), 6.07-5.89 (m, 1H), 5.46-5.23 (m, 2H), 5.00 (s, 2H), 4.71 (dt, J = 5.7, 1.4 Hz, 2H), 3.45 (s, 3H), 3.33 (s, 3H), 3.28 (s, 1H), 2.07 (d, J = 17.6 Hz, 1H), 2.05-1.57 (m, 12H). ^{13}C NMR (101 MHz, $CDCl_3$) δ 166.4, 153.8, 149.9, 148.5, 139.8, 139.5, 138.9, 138.6, 137.0, 136.8, 133.8, 133.0, 132.3, 129.8, 129.7, 129.5, 128.2, 127.9, 125.8, 125.3, 120.3, 119.5, 118.5, 75.2, 65.5, 57.5, 40.0, 39.3, 39.2, 38.8, 37.2, 33.1, 29.9, 28.5, 28.3. MS (ES+): m/z calc. for $C_{38}H_{38}ClN_3O_{10}S$: 763.2; found: 764.6 $[M + 1]^+$.

Compound 13:

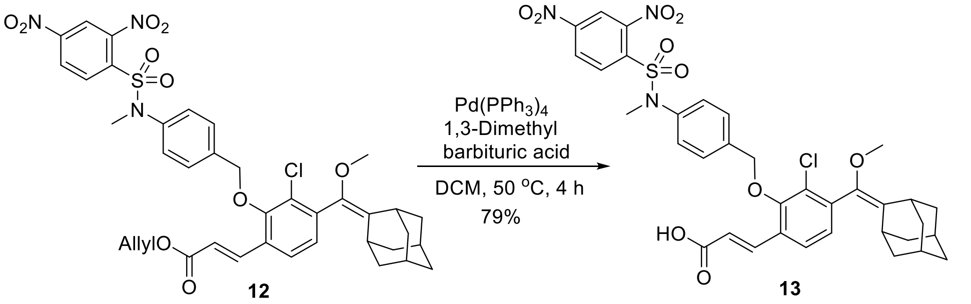

A mixture of compound **12** (50 mg, 0.066 mmol, 1 eq), DMBA (31 mg, 0.196 mmol, 3 eq) and $Pd(PPh_3)_4$ (8 mg, 0.006 mmol, 0.1 eq) in DCM (2 mL) was stirred at 50 °C for 3 hours. Upon completion, the reaction mixture was concentrated under reduced pressure and crude mixture was purified by preparative RP-HPLC (50-100% ACN in water, 20 min) to afford compound **13** (38 mg, 79%) as a white solid. ^{1}H NMR (400 MHz, $CDCl_3$) δ 8.44 (d, J = 2.2 Hz, 1H), 8.33 (dd, J = 8.7, 2.2 Hz, 1H), 7.98 (d, J = 16.1 Hz, 1H), 7.74 (d, J = 8.7 Hz, 1H), 7.51 (dd, J = 8.2, 3.7 Hz, 3H), 7.27 (d, J = 8.4 Hz, 2H), 7.14 (d, J = 8.0 Hz, 1H), 6.47 (d, J = 16.1 Hz, 1H), 5.43 (s, 1H), 5.02 (s, 2H), 3.44 (s, 3H), 3.35 (s, 3H), 3.29 (s, 1H), 2.09 (s, 1H), 2.01-1.57 (m, 12H). ^{13}C NMR (101 MHz, $CDCl_3$) δ 171.6, 154.0, 149.9, 148.5, 141.1, 139.8, 139.4, 139.1, 136.9, 136.8, 133.7, 133.3, 129.8, 129.2, 128.3, 127.8, 125.7, 125.6, 119.5, 75.3, 57.5, 40.0, 39.2, 38.8, 37.1, 33.1, 29.9, 28.3. MS (ES+): m/z calc. for $C_{35}H_{34}ClN_3O_{10}S$: 723.2; found: 724.5 $[M+H]^+$.

Probe 3:

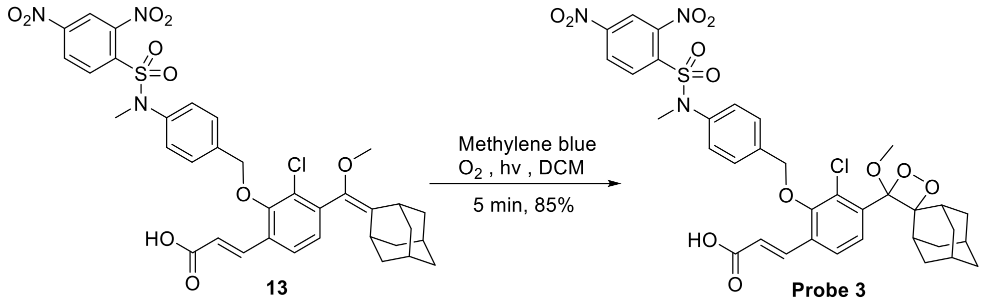

Enol-ether **13** (30 mg, 0.041 mmol, 1 eq) and catalytic amount of methylene blue (~3 mg) were dissolved in 8 mL of DCM. Oxygen was bubbled through the solution while irradiating with yellow light for 5 minutes. The reaction was monitored by RP-HPLC. After completion, the reaction mixture was concentrated by evaporation under reduced pressure. The crude product was purified by preparative RP-HPLC [70-100% ACN in water (0.1 % TFA), 20 min] to afford **probe 3** as a white solid (26.3 mg, 85%). ^{1}H NMR (400 MHz, DMSO-d$_6$) δ 8.98 (d, *J* = 2.3 Hz, 1H), 8.51 (dd, *J* = 8.8, 2.3 Hz, 1H), 7.95 (d, *J* = 8.4 Hz, 1H), 7.89 (d, *J* = 8.8 Hz, 1H), 7.80 (d, *J* = 8.4 Hz, 1H), 7.67 (d, *J* = 16.1 Hz, 1H), 7.49 (d, *J* = 8.5 Hz, 2H), 7.31-7.27 (m, 2H), 6.61 (d, *J* = 16.1 Hz, 1H), 4.94 (d, *J* = 2.5 Hz, 2H), 3.95 (s, 1H), 3.32 (s, 3H), 3.12 (s, 3H), 2.88 (s, 1H), 2.22 (d, *J* = 12.1 Hz, 1H), 1.90 (s, 1H), 1.90-1.16 (m, 11H). ^{13}C NMR (101 MHz, DMSO-d$_6$) δ 167.7, 154.1, 150.9, 148.5, 140.4, 137.1, 136.4, 134.8, 132.8, 132.00, 129.9, 129.2, 127.8, 127.3, 126.7, 123.6, 120.7, 111.8, 96.0, 75.5, 50.0, 36.5, 33.9, 33.7, 32.4, 32.2, 31.7, 26.1, 25.8. MS (ES+): m/z calc. for $C_{35}H_{34}ClN_3O_{12}S$: 755.2; found: 756.5 $[M+H]^+$.

Probe 4:

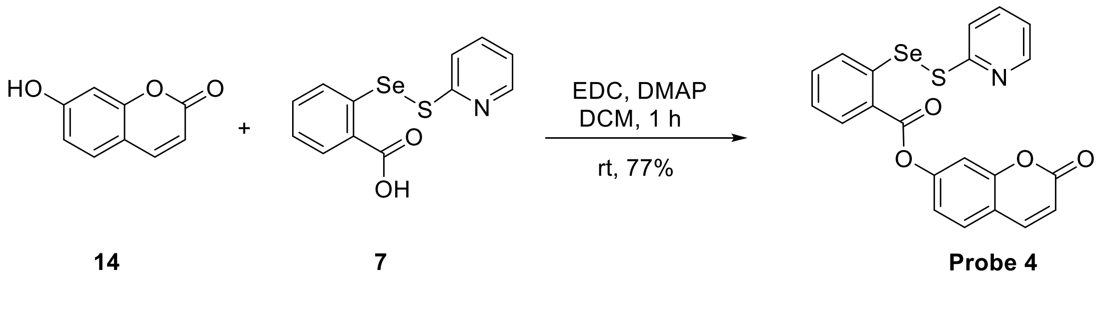

Compound **14** (50 mg, 0.308 mmol, 1 eq), compound **7** (70 mg, 0.617 mmol, 2 eq) and 4-DMAP (75 mg. 0.0.617 mmol, 2 eq) were dissolved in DCM (4 mL). Then EDC (48 mg, 0.308 mmol, 1 eq) was added and the reaction mixture was stirred for 60 minutes at room temperature. After consumption of compound **14** (as indicated by TLC) the reaction was diluted with EtOAc (30 mL), washed with 1M HCl (10 mL), dried over Na_2SO_4 and concentrated under reduced pressure. The crude mixture was purified using silica column chromatography to elute pure **probe 4** as a white solid (108 mg, 77%). ^{1}H NMR (400 MHz, CDCl$_3$) δ 8.48 – 8.43 (m, 1H), 8.34 (dd, *J* = 7.8, 1.4 Hz, 1H), 8.14

(d, J = 8.2 Hz, 1H), 7.74 (d, J = 9.6 Hz, 1H), 7.62 – 7.52 (m, 4H), 7.45 – 7.37 (m, 1H), 7.30 – 7.21 (m, 2H), 7.07 (ddd, J = 6.7, 4.9, 1.8 Hz, 1H), 6.44 (d, J = 9.6 Hz, 1H). ^{13}C NMR (101 MHz, $CDCl_3$) δ 165.7, 160.3, 158.3, 154.9, 153.1, 149.6, 142.9, 139.4, 137.5, 134.7, 132.2, 128.9, 128.2, 126.6, 126.1, 122.1, 121.0, 118.6, 117.2, 116.6, 110.7. MS (ES+): m/z calc. for $C_{21}H_{13}N_3O_4SSe$: 455.0; found: 456.3 $[M+H]^+$.

3. Spectroscopic Data:

3a. Evaluation of the selectivity of probes 1-3 towards H_2S and RSH.

In this assay, we studied the detection selectivity of **probes 1-3** towards H_2S and RSH. Both **probe 1** and **2** presented high selectivity for detection H_2S in comparison to RSH, while **probe 3** presented much lower selectivity (**Figure S1**).

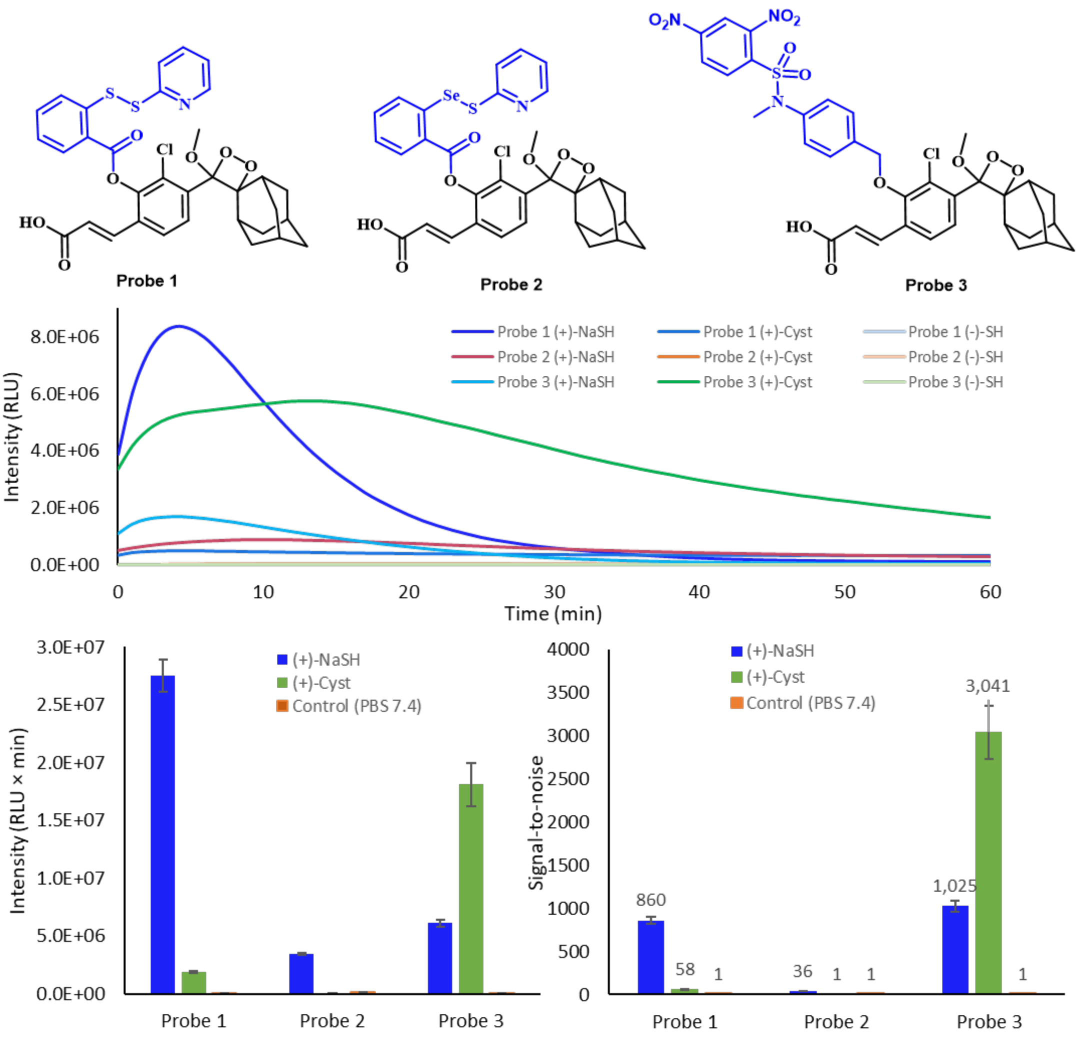

Figure S1: Chemiluminescence kinetic profiles (Up, over 60 min), total light emission (down left, over 4 min) and signal-to-noise (down right, over 4 min) of **probes 1-3** [10 µM] in PBS(pH 7.4), in the presence and absence of L-cysteine-hydrochloride and NaSH [100 µM] (The probes were pre-incubated for 30 minutes at 27 ^{O}C in PBS (pH 7.4) prior to use).

3b. Determination of the Limit of Detection (LOD) of probe 1 for detection of H_2S:

In this measurement, the LOD of **probe 1** was determined using various concentrations of NaSH. (LOD = 2.3×10^{-7} M or 0.0128 µg/mL).

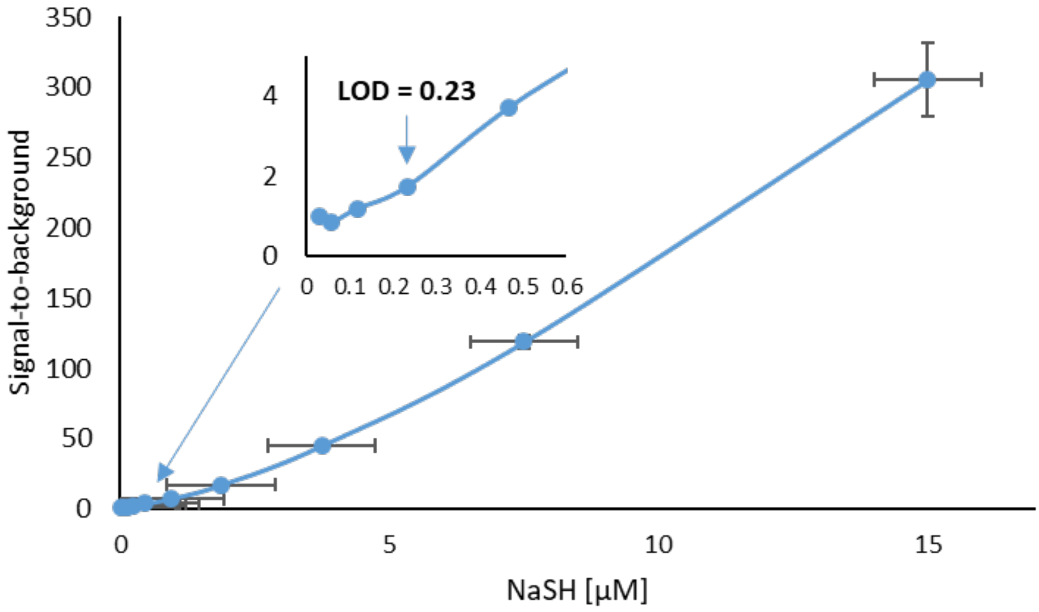

Figure S2: Signal/noise ratio values for **probe 1** [10 µM], 10% DMSO in PBS (pH 7.4) plotted against different NaSH concentrations.

3c. Detection of H_2S-released from antibiotics degradation by βLBC.

General assay protocol:

All antibiotics stocks solutions were prepared using DMSO or water according to their solubility. All enzyme stocks solutions were prepared according to their manufacturer solubility protocol. Two-step protocol was used to detect H_2S released from antibiotics [1 mM] after incubation with enzyme [**βLBC** = 10 U/mL and all others 2 U/mL] using a chemiluminescent **probes 1-3** [25 μM].

Two-step protocol:

For 25 μM assay (final volume of assay 0.250 mL):

1. 0.175 mL PBS (pH 7.4) + 5 μL antibiotic stock 50 mM in DMSO (final conc. 1 mM) + 20 μL **βLBC** [100 U/mL] stock solution in PBS (final conc. 10 U/mL). [**For other enzymes**; 0.193 mL PBS + 5 μL antibiotic stock 50 mM in DMSO (final conc. 1 mM), 2 μL enzyme [200 U/mL] stock solution in PBS (final conc. 2 U/mL)].

Negative controls (background): 0.195 mL PBS + 5 μL antibiotic stock of 50 mM in DMSO (final conc. 1 mM).

Incubation of plate for 30 min at room temperature.

2. Addition of 50 μL dioxetane **probe 1** working solution [Pre-incubated for 45 min, 0.750 mL PBS + 50 μL of 2 mM probe (final conc. 25 μM)]. The luminescence was measured every minute for 2 h with SpectraMax i3x plate reader at 27 ^{o}C.

Results of the antibiotics with **probe 1**, producing a high signal-to-noise ratio shown in the manuscript (**Figure 4**). Whereas antibiotics, which are producing a low signal-to-noise ratio are presented below (**Figure S3**):

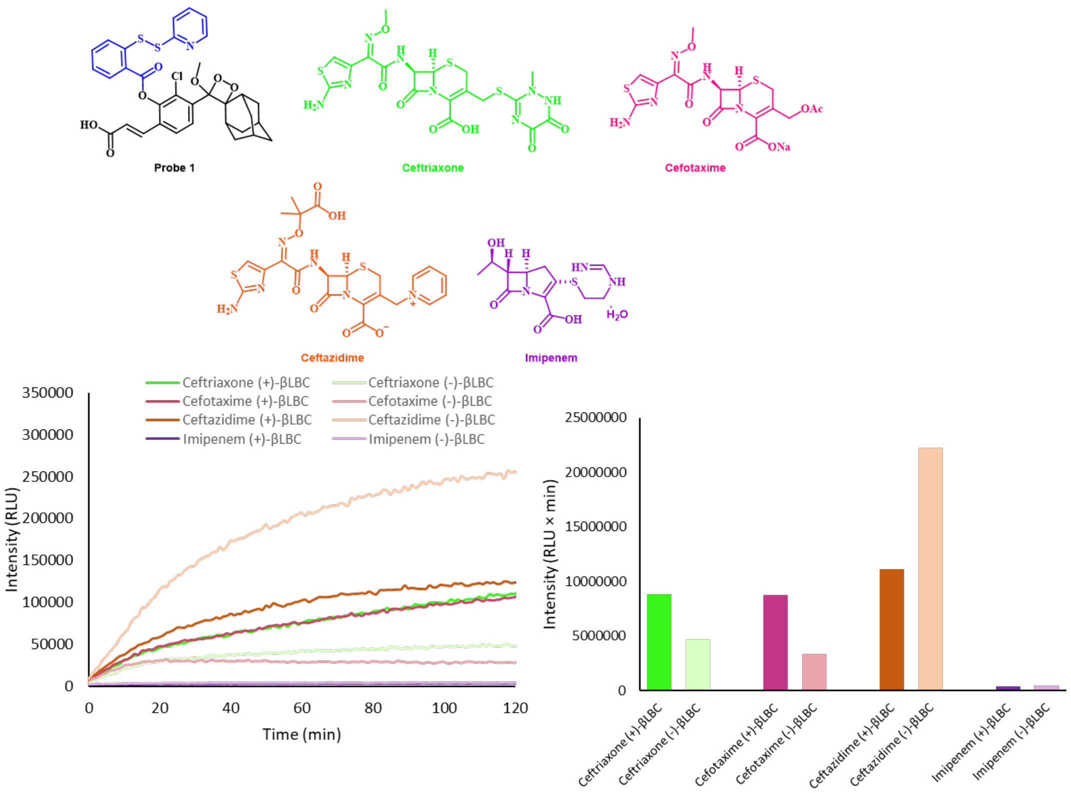

Figure S3: Chemiluminescence kinetic profiles [Left] and total light emission [Right] (over 2 h) produced by **probe 1** [25 μM] in PBS (pH 7.4) with four different antibiotics [1 mM] in the presence and absence of **βLBC** [10 U/mL]. **Probe 1** was pre-incubated for 45 min in PBS (pH 7.4) prior to use; antibiotics and **βLBC** were incubated for 30 min prior to measurement.

3d. Formation of byproduct A upon biodegradation of probe 1 in the presence of ceftizoxime or cefalexin with βLBC

In this experiment, RP-HPLC chromatograms showing gradual formation of byproduct **A** upon decomposing of **probe 1** [100 µM] in the presence of Cefalexin [3 mM] and **βLBC** [20 U/mL] (**Figure S4)**. The formation of byproduct A and Benzoate were confirmed by LCMS and their authentic samples by cross injection in HPLC. The same byproduct **A** detection experiment with ceftizoxime explained in manuscript (**Figure 6**).

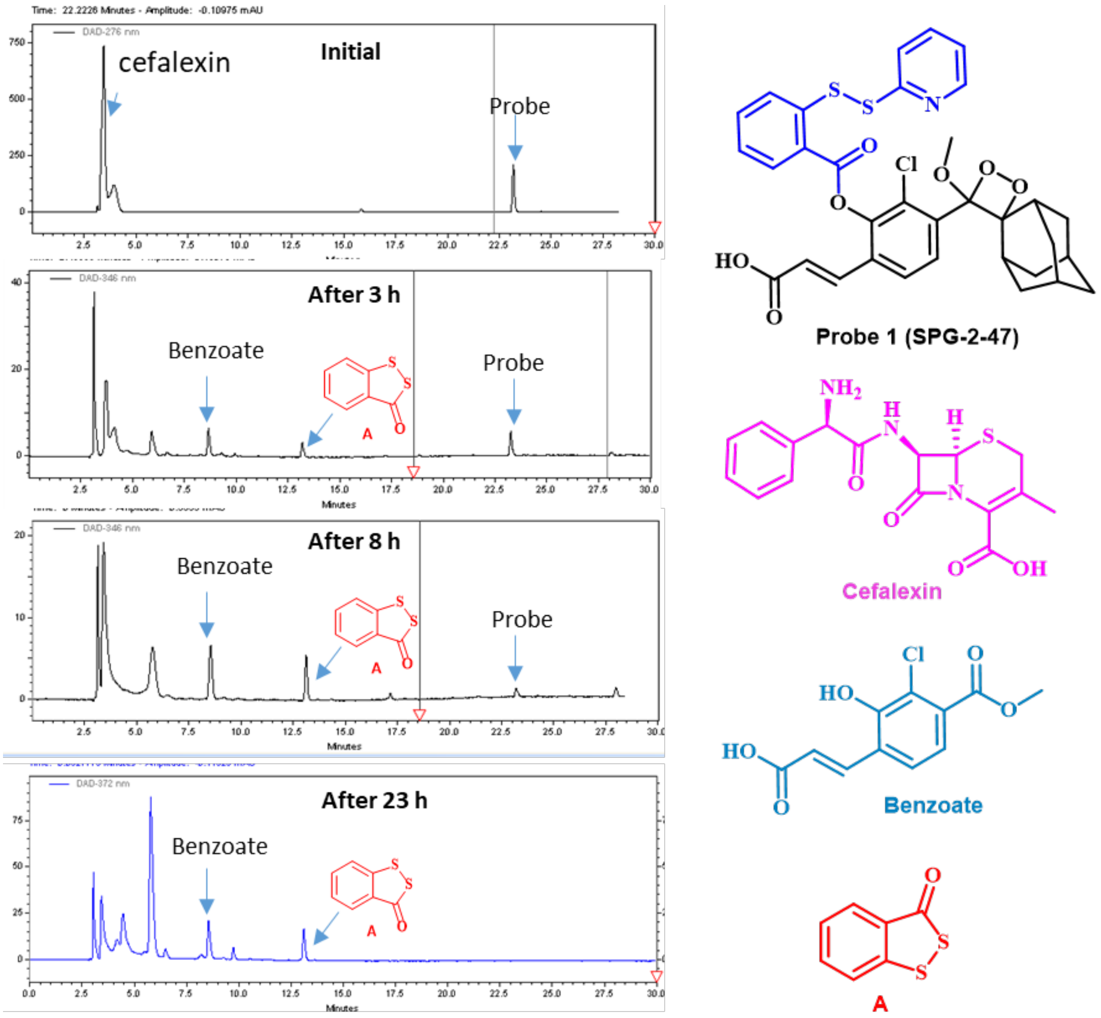

Figure S4: RP-HPLC chromatograms showing gradual formation of byproduct **A** upon decomposing of **probe 1** [100 µM] in the presence of Cefalexin [3 mM] and **βLBC** [20 U/mL]. HPLC elution gradient ACN and water with 0.1 TFA (30-100%).

3e. General procedure for the data given in Figure 8 (Detection of antibiotic resistant bacteria using probe 1).

Eight antibiotic resistant bacterial strains were inoculated from agar plates into culture tubes with nutrient broth (5 g/L peptone, 5 g/L NaCl; 2 g/L yeast extract, 1 g/L meat extract, pH 7.4) and incubated for 19 h at 37 °C and 150 rpm. Broth cultures were diluted to an optical density (600 nm) of 0.1 with sterile saline (0.9% NaCl). In wells of a white 96-well plate, 0.1 mL culture samples were mixed with 90 µL PBS and 10 µL Ceftizoxime or sulopenem stock solution (25 mM, final concentration 1 mM) and incubated for 30 min at room temperature. Then, 50 µL PBS with 0.125 mM of **probe 1** (5x working solution, pre-incubated 1 h at room temperature) was added and luminescence (relative light units, RLU) was measured for 1 h at room temperature with a plate reader (500 ms integration time). Results of assay explained in the manuscript (**Figure 8**).

3f. Detection of carbapenem-resistant bacteria with Faropenem and probe 3.

Two imipenem (a carbapenem) resistant bacterial strains, *Pseudomonas aeruginosa* RKI 48/09 (VIM-15) and *Klebsiella pneumonia* RKI 92/08 (KPC-2), and a cephalosporinase-positive strain, *Escherichia coli* RKI 66/09 (CMY-2, AmpC), were inoculated from agar plates into culture tubes with nutrient broth and incubated for 22 h at 37°C and 150 rpm. In parallel, the two carbapenemase-positive strains were cultivated in nutrient broth supplemented with 8 and 4 mg/L imipenem (Imp), respectively. In addition, antibiotic sensitive strains *Pseudomonas aeruginosa* ATCC10145, *Klebsiella pneumoniae* RKI 2867/81and *Escherichia coli* ATCC 25922 were similarly cultivated in nutrient broth. Broth cultures were diluted to an optical density (600 nm) of 0.5 with sterile saline (0.9% NaCl). In wells of a white 96-well plate, 0.19 ml cell suspension samples were mixed with 10 µL Faropenem stock solution (20 mM in dimethyl sulfoxide, final concentration 0.8 mM) and 50 µL 5x working solution of **probe 3** (0.125 mM in PBS, final concentration 25 µM). Luminescence was measured for 1 h at room temperature in a plate reader (500 ms integration time). Results are shown in **Figure S5**.

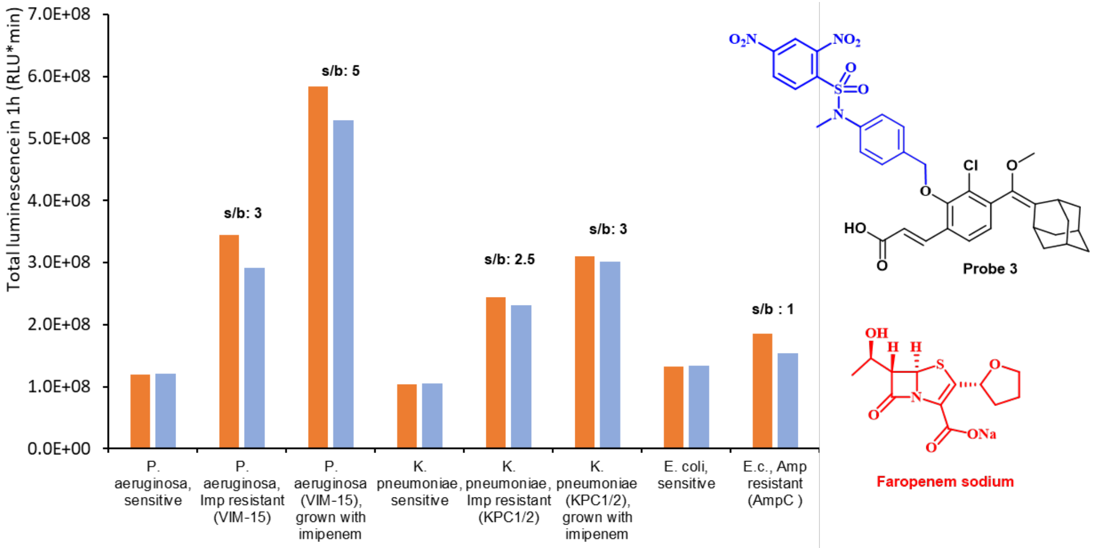

Figure S5: Integrated luminescence signals and signal-to-background (s/b) for antibiotic resistant bacteria and sensitive reference strains after addition of Faropenem [0.8 µM] and **probe 3** [25 µM]. Background: antibiotic sensitive reference strain of the same species. Orange color bars: Assay 1, blue color bars: Assay 2.

3g. General procedure for the data given in Figure 9 (one-step assay for antibiotic resistant bacteria with probe 1 and inhibition effect-obtained by 3-Aminophenylboronic acid).

Two strains expressing serine-type beta-lactamases, *Klebsiella pneumonia* RKI 92/08 (KPC-2) and *Escherichia coli* RKI 66/09 (CMY-2, AmpC) and two antibiotic sensitive reference strains, *Klebsiella pneumonia* RKI 2867/81 and *Escherichia coli* ATCC 25922 were inoculated from agar plates into culture tubes with nutrient broth and incubated for 19 h at 37°C and 150 rpm. Broth cultures were diluted to an optical density (600 nm) of 1.0 with sterile saline (0.9% NaCl). In wells of a white 96-well plate 90 µL PBS with 50 µM of 0.125 mM **probe 1** (pre-incubated in PBS for 1 h at room temperature, final concentration 25 µM) was mixed with 100 µL cell suspension OD600 1.0 (final OD600 0.4) or sterile medium and 10 µL of 25 mM Cefazolin (final concentration 1 mM). In addition, 7 mM (final concentration) of the known serine beta-lactamase inhibitor 3-aminophenylboronic acid was added to a subset of wells. Immediately after all reagents had been added, luminescence (relative light units, RLU) was measured at room temperature for 15 min every min with a plate reader (500 ms integration time). Results are shown in the manuscript (**Figure 9**).

3h. Determination of the Limit of Detection (LOD) of probe 1 for the detection of antibiotic resistant bacteria using Ceftizoxime:

Strains cultivated in nutrient broth overnight at 37°C, cell density set to 10^9 CFU/mL by optical density, dilution series prepared in sterile saline. Assay in white 96-well plate: 0.1 mL cell suspension mixed with 0.1 mL PBS with 2.5 mM Ceftizoxime and 2.5% DMF (Final Ceftizoxime concentration: 1.25 mM) background control wells: 0.1 mL cell supsension mixed with 0.1 mL PBS with 2.5% DMF 96-well plate incubated for 30 min at room temperature. 0.05 mL PBS with 0.125 mM AquaSpark S-S Probe added per well (final probe concentration 25 µM) Luminescence measured for 30 min. Limit of Detection (LOD) = 3.1^6 cells of antibiotic resistant bacteria.

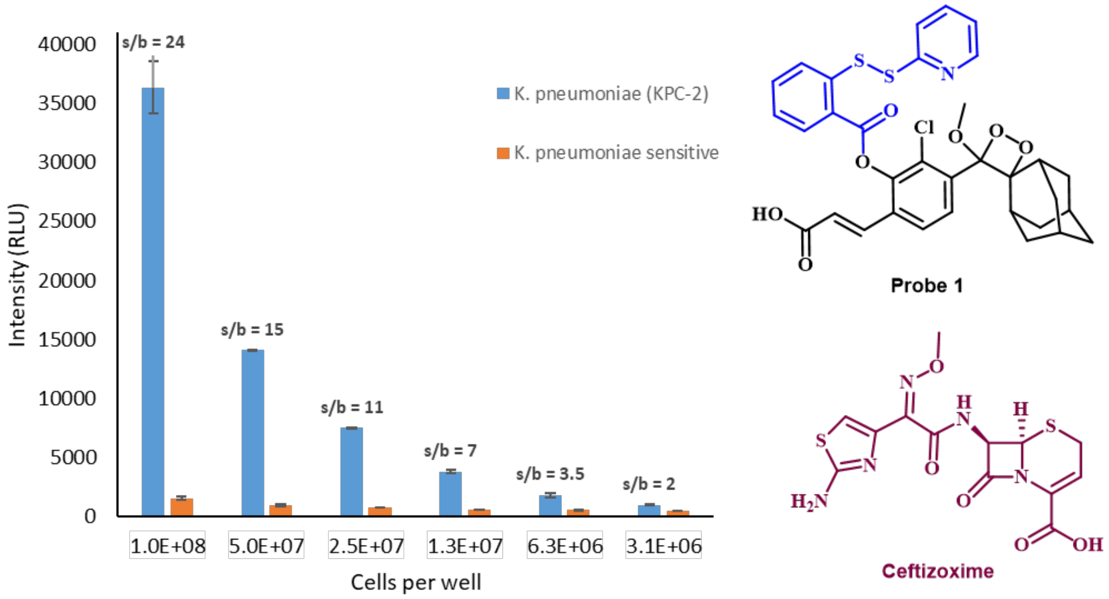

Figure S6: Integrated luminescence signals and signal-to-background (s/b) for antibiotic resistant bacteria and sensitive reference strain [1.0^9 to 3.1^6 CFU/mL] after addition of Ceftizoxime [1.23 mM] and **probe 3** [25 µM]. Background: antibiotic sensitive reference strain of the same species.

3i. Determination of the relative rate constant of the reaction of hydrogen sulfide release by β-lactamase-catalyzed β -lactam biodegradation of various antibiotics.

The order of reaction is pseudo zero. Because the concentration of antibiotics [1 mM] used in the experiment is considerably high in compare with the concentration of **probe 4** [25 μM], the order of the reaction could be considered pseudo zero order. The constants below were calculated from the florescent probe activation assay after the conversion of RLU measured to concentration of H_2S emitted. In pseudo zero order reaction the slope of such graph would represent the rate constant of the reaction.

Equation

R = K [antibiotics concentration]^0

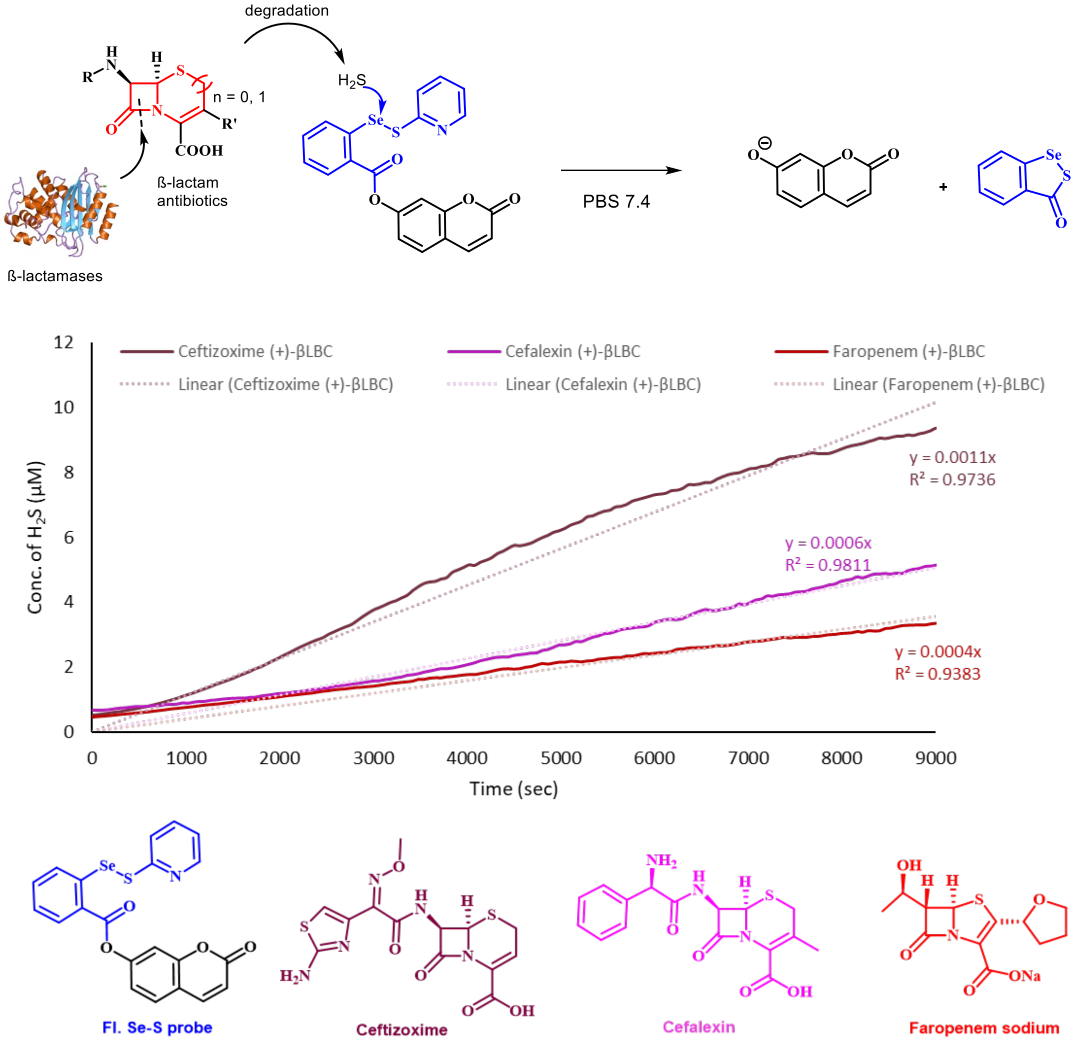

Figure S7: Correlation between H_2S release *vs* time upon incubation with **probe 4** [25 μM] in PBS, pH 7.4, and antibiotics [1 mM] in the presence and absence of enzyme (Beta-lactamase from *Bacillus cereus*) [10 U/mL]. Antibiotics and **βLBC** were incubated for 30 min prior to measurement.

Sr. No.	Antibiotics	Rate constant (K)
**1**	**Ceftizoxime**	**1.1 × 10^{-3} µM/s**
**2**	**Cefalexin**	**6.0 × 10^{-4} µM/s**
**3**	**Faropenem**	**4.0 × 10^{-4} µM/s**

Table S1: Rate constant of the reaction of hydrogen sulfide release by β-lactamase-catalyzed β-lactam biodegradation of various antibiotics.

4. NMR and MS Spectra:

^{1}H-NMR and ^{13}C-NMR spectra of compound **3:**

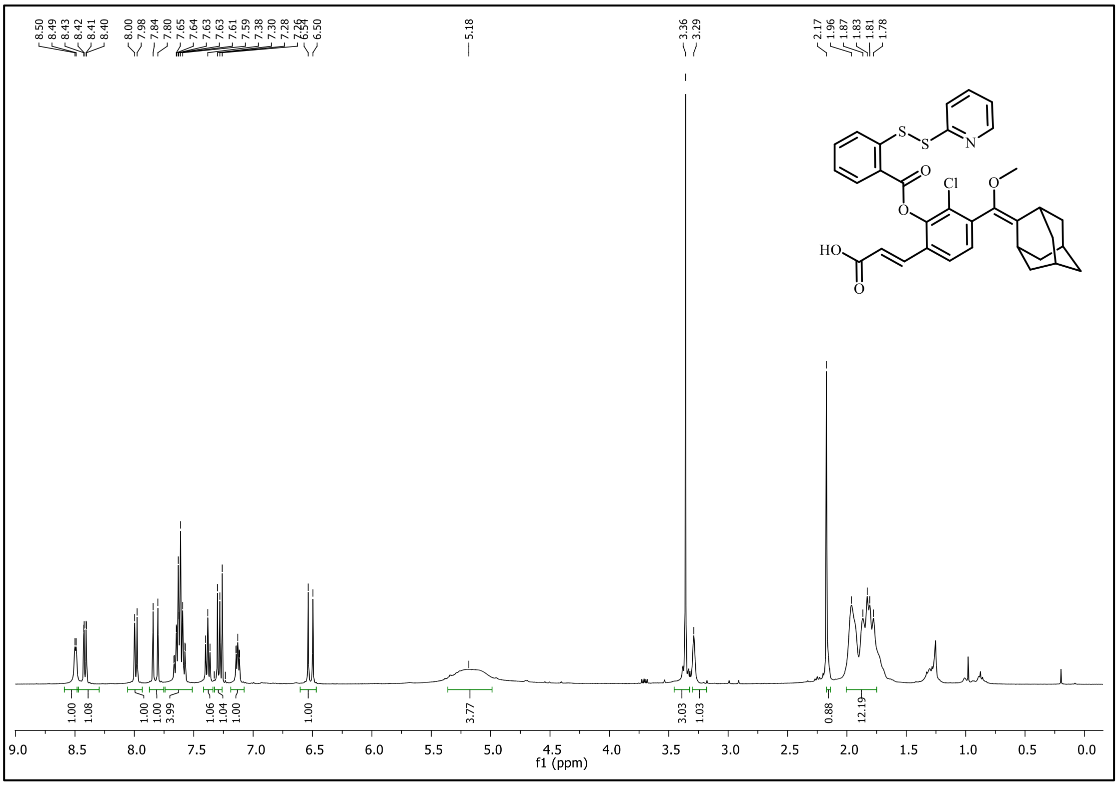

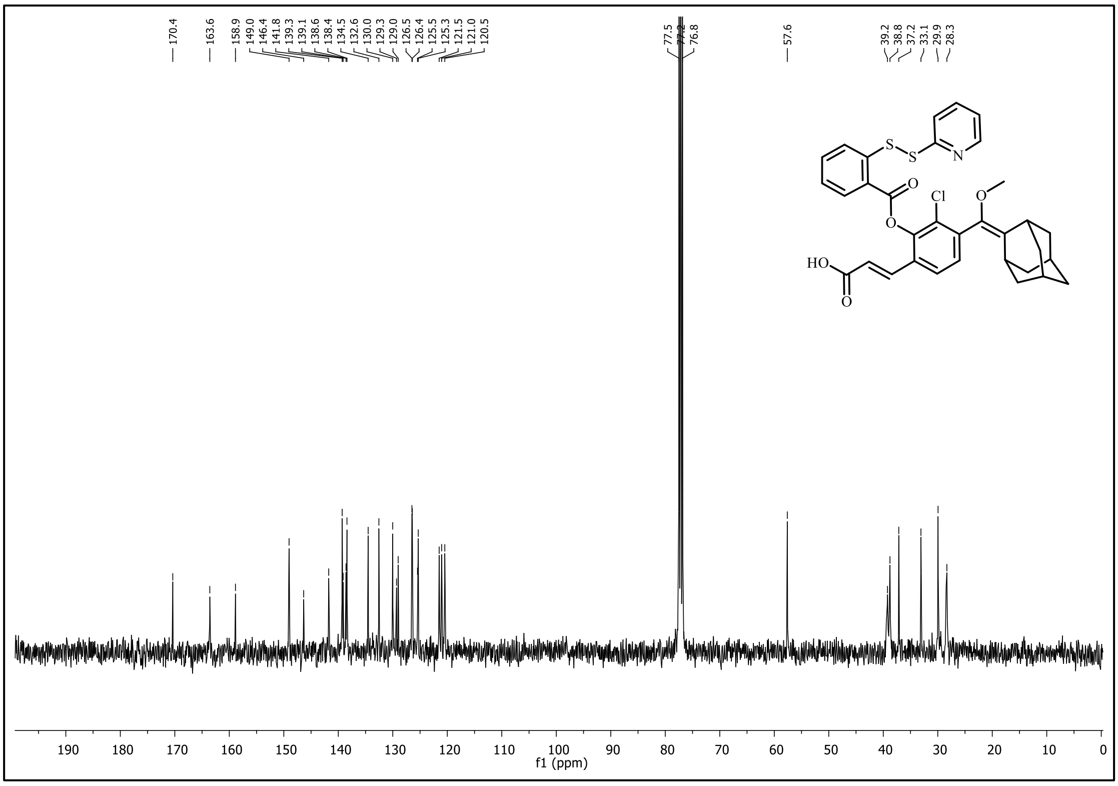

MS of compound **3**

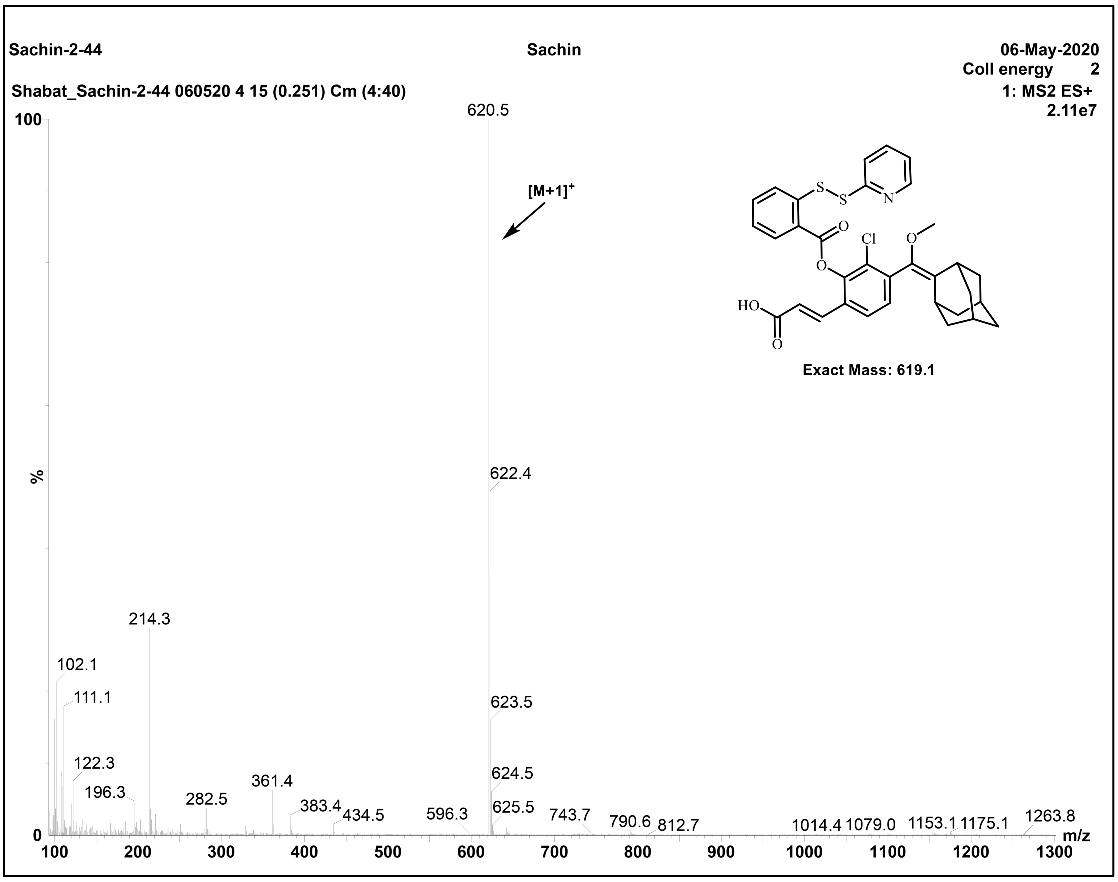

^{1}H-NMR and ^{13}C-NMR spectra of **probe 1:**

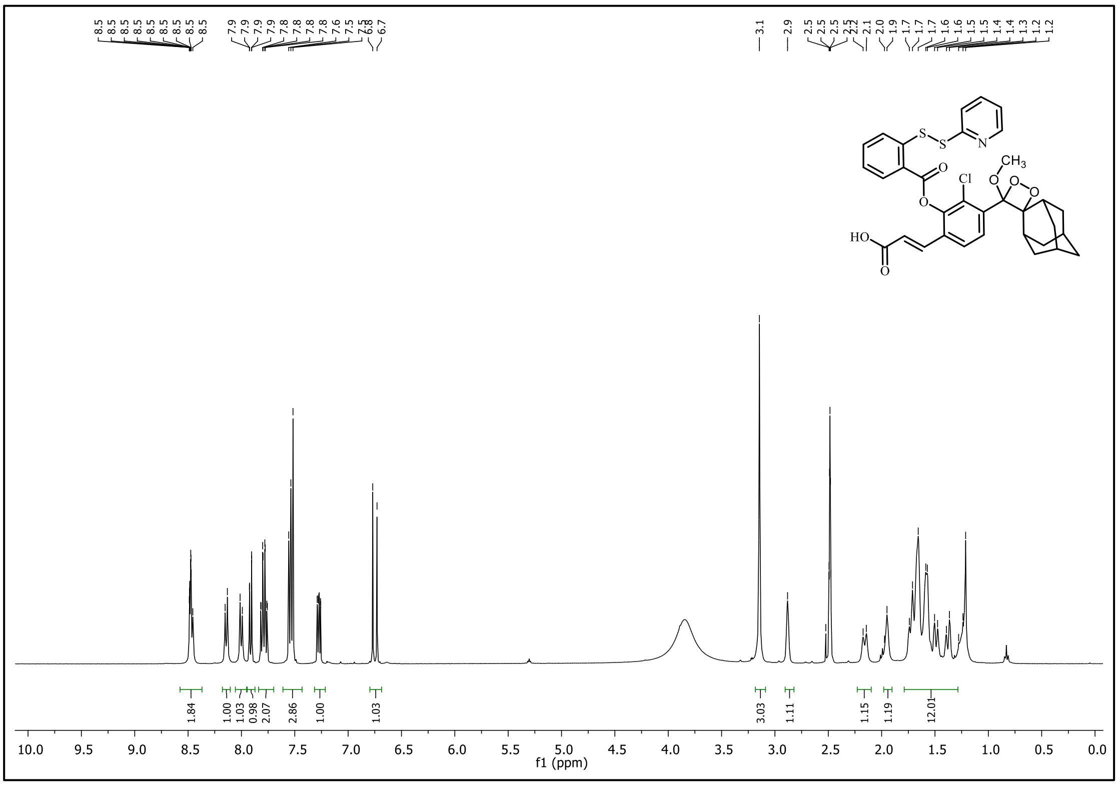

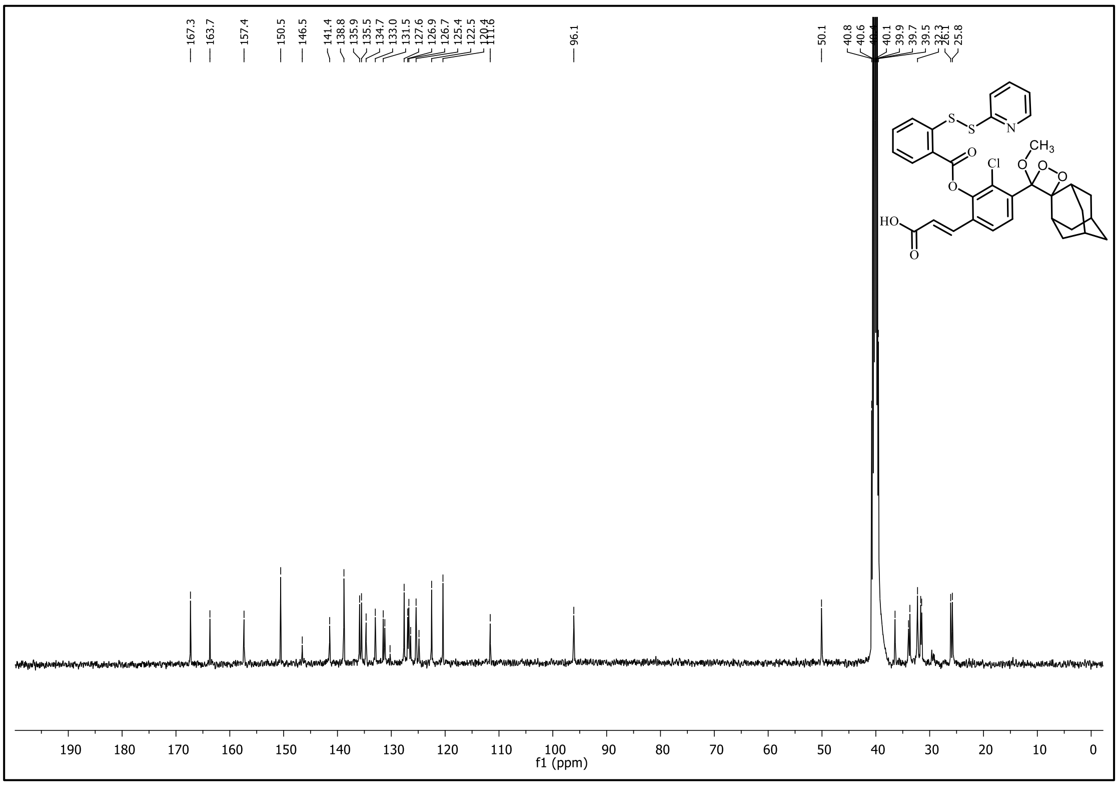

MS of **probe 1:**

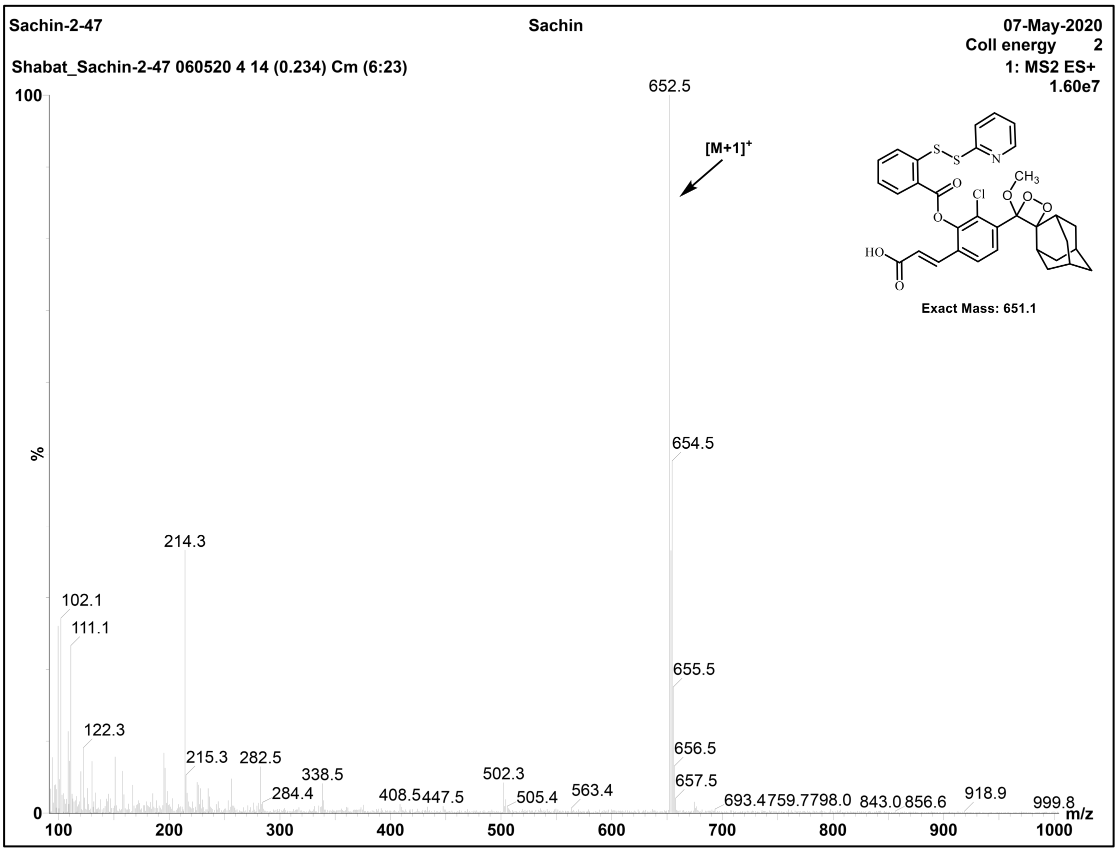

Sachin-2-47
Sachin
07-May-2020
Coll energy 2
Shabat_Sachin-2-47 060520 4 14 (0.234) Cm (6:23)
1: MS2 ES+
1.60e7
100
%
0
652.5
[M+1]+
654.5
214.3
102.1
111.1
655.5
122.3
656.5
215.3
282.5
338.5
502.3
657.5
284.4
408.5
447.5
505.4
563.4
693.4
759.7
798.0
843.0
856.6
918.9
999.8
m/z
100
200
300
400
500
600
700
800
900
1000
S
S
N
O
O
Cl
CH3
O
O
O
HO
O
Exact Mass: 651.1

^{1}H-NMR and ^{13}C-NMR spectra of compound **6:**

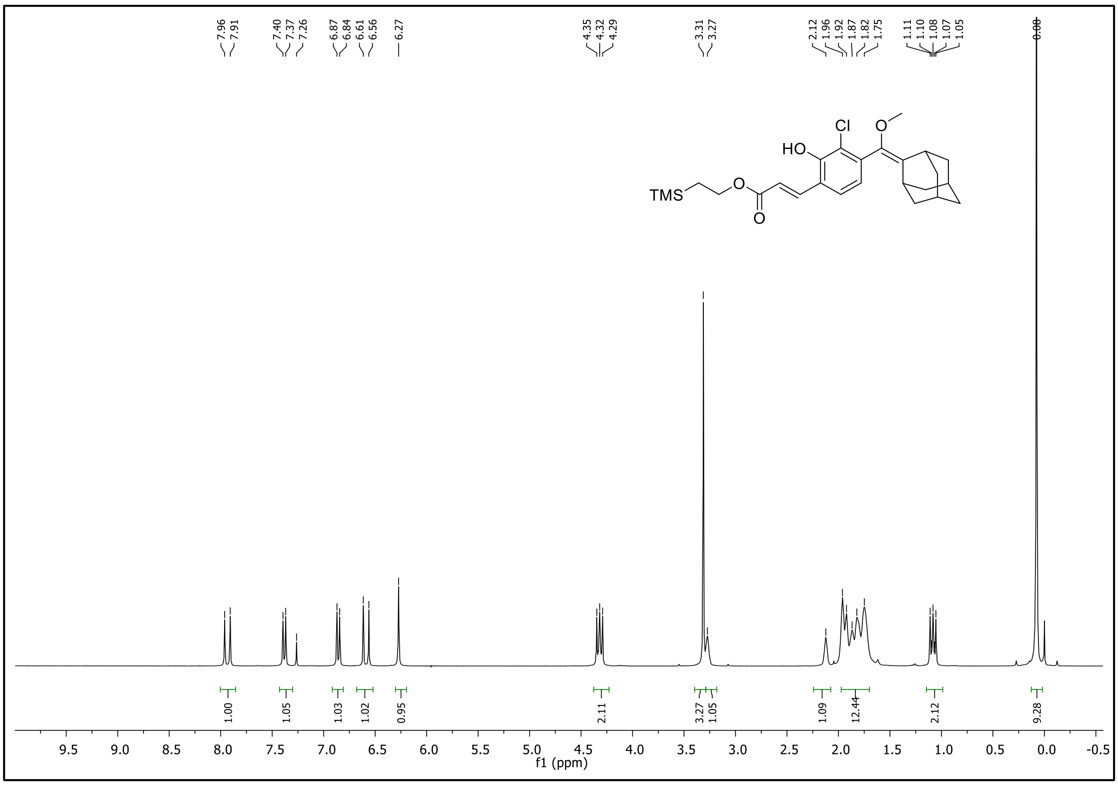

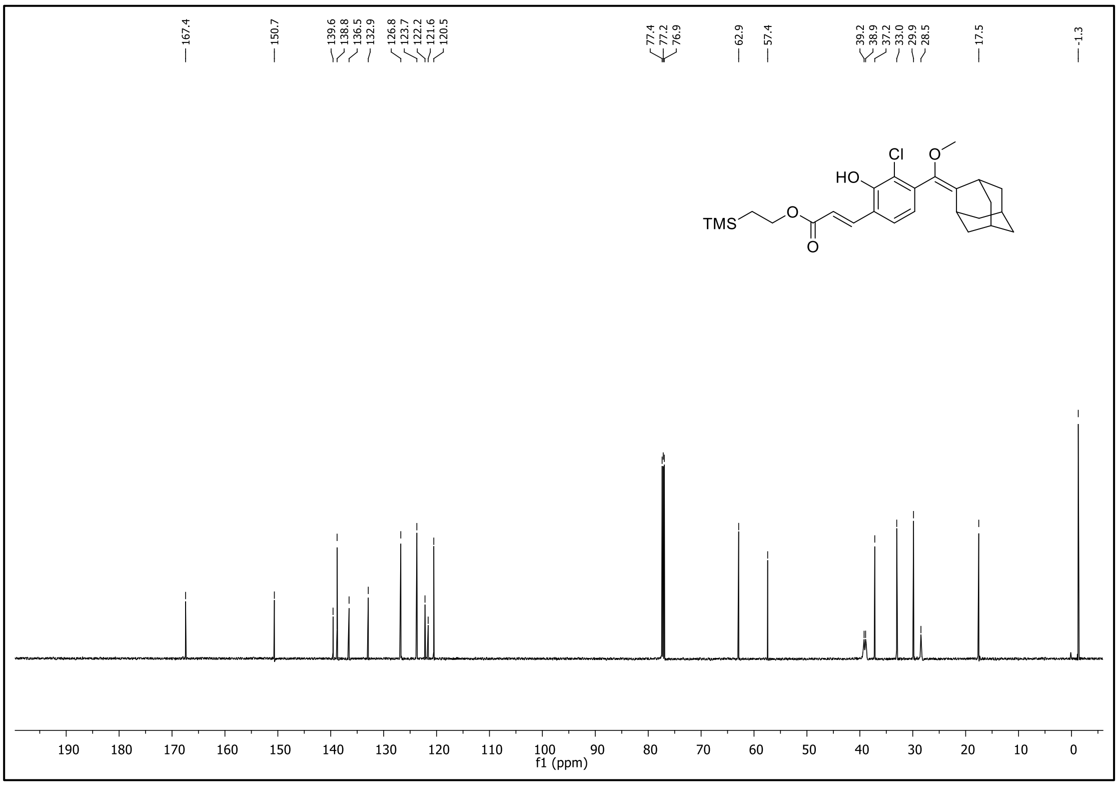

MS of compound **6**

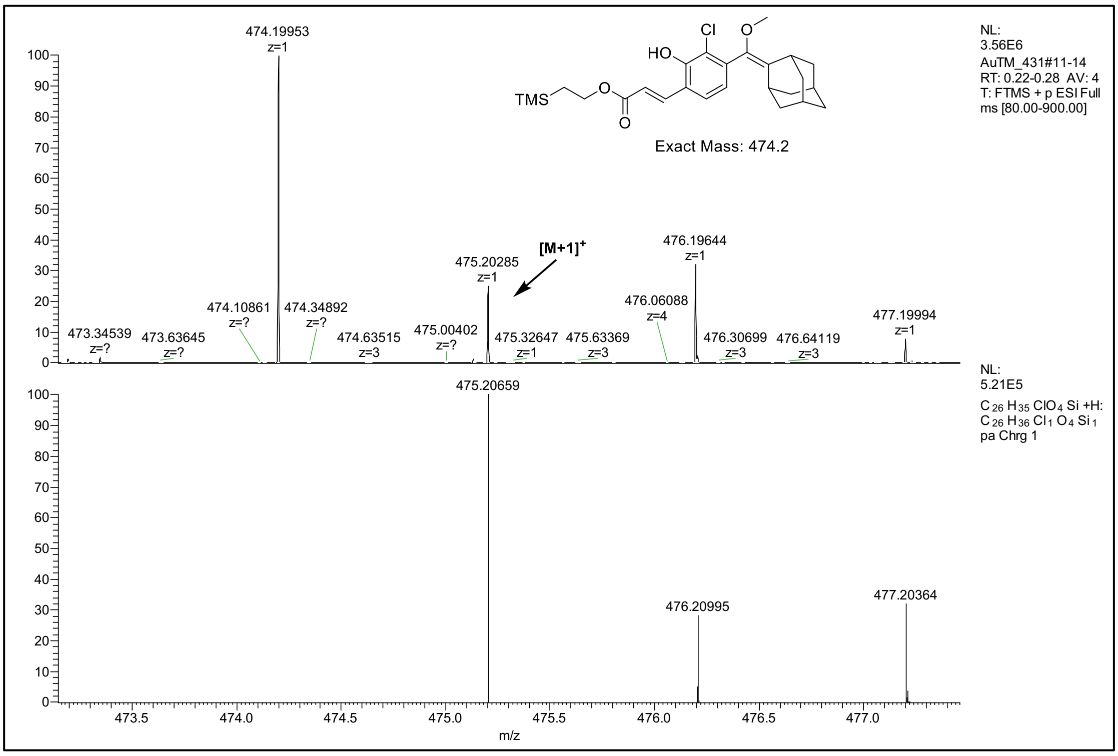

^{1}H-NMR and ^{13}C-NMR spectra of compound **8:**

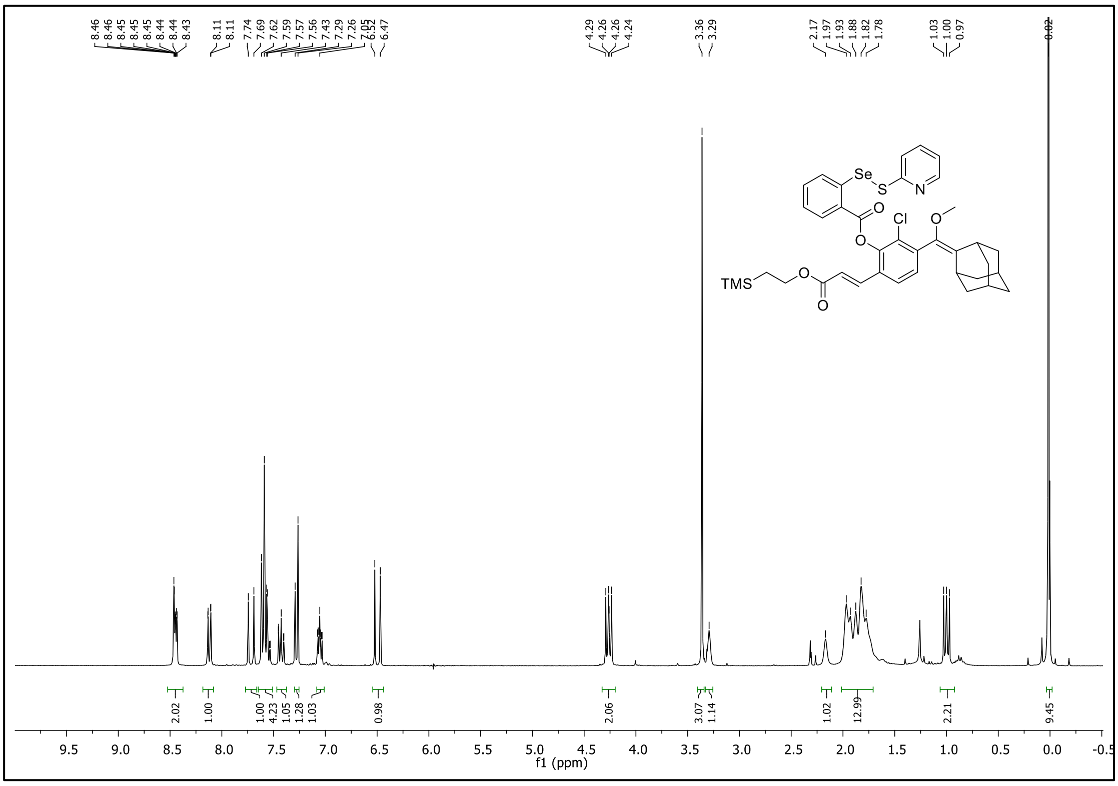

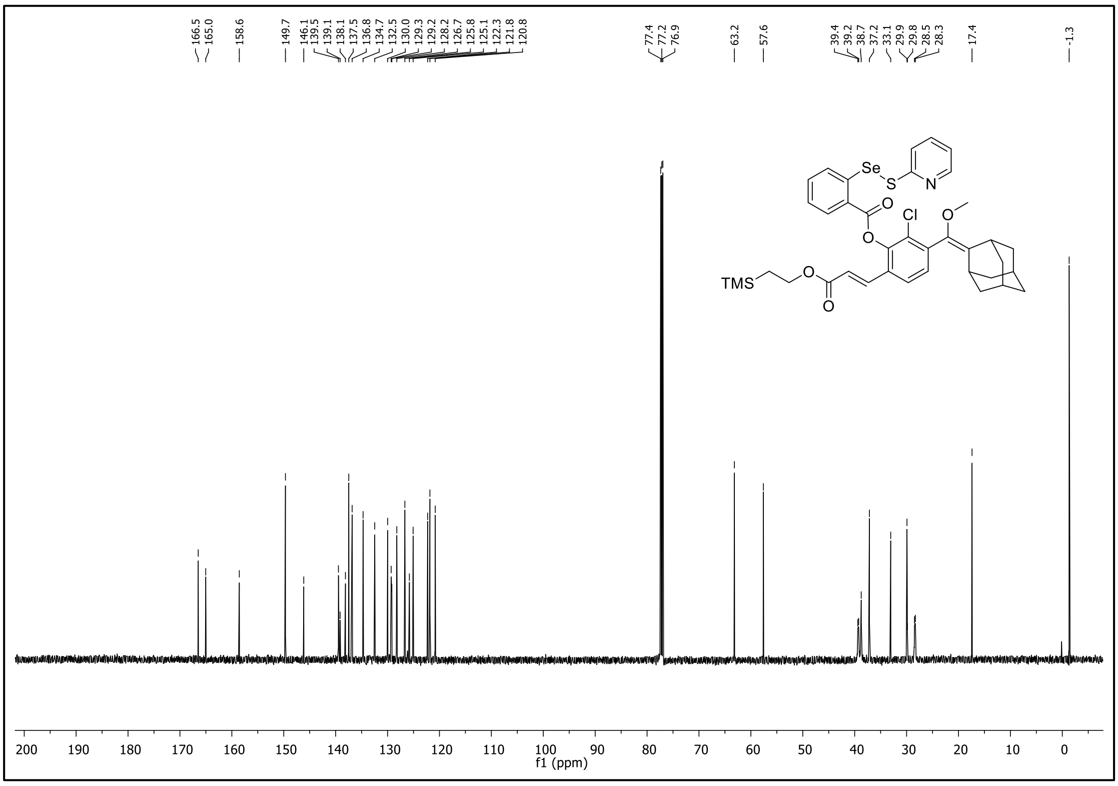

MS of compound **8:**

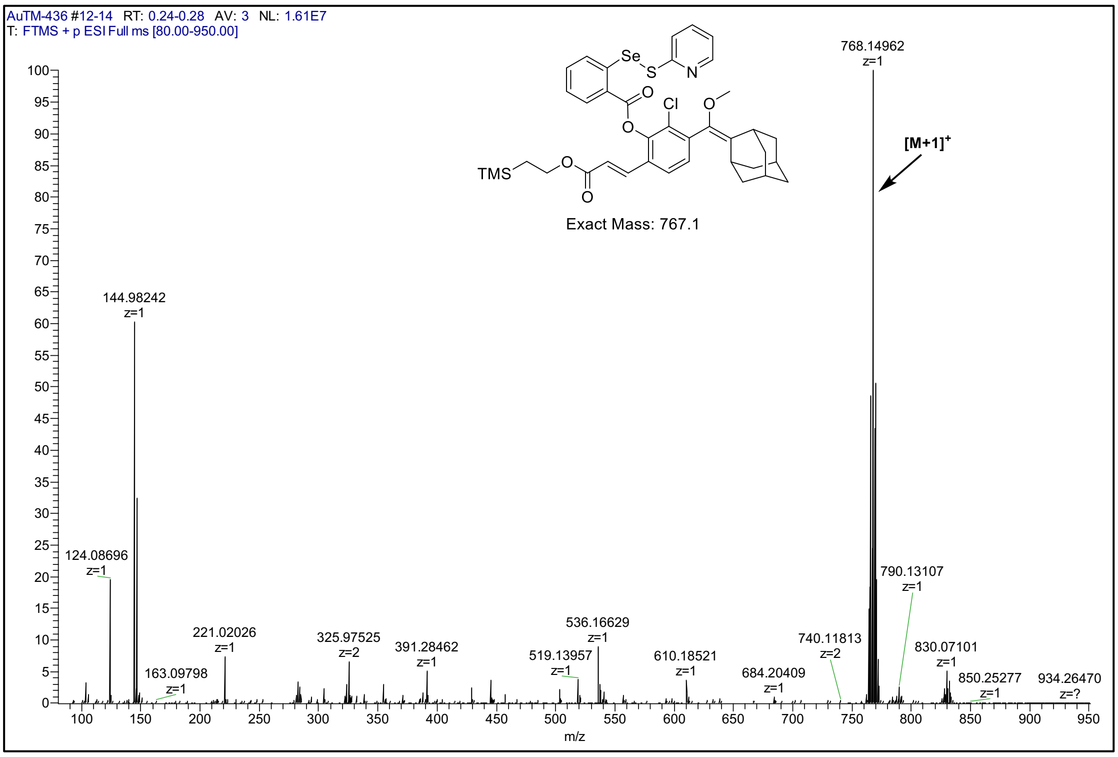

^{1}H-NMR and ^{13}C-NMR spectra of compound **9:**

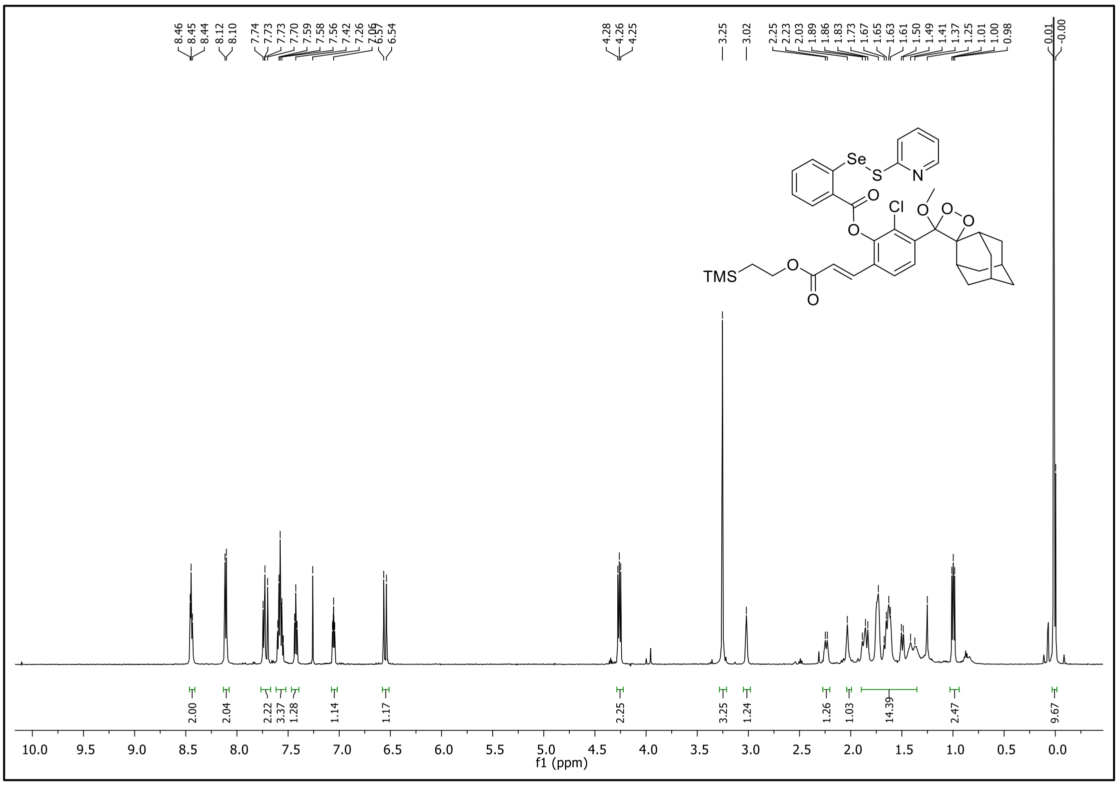

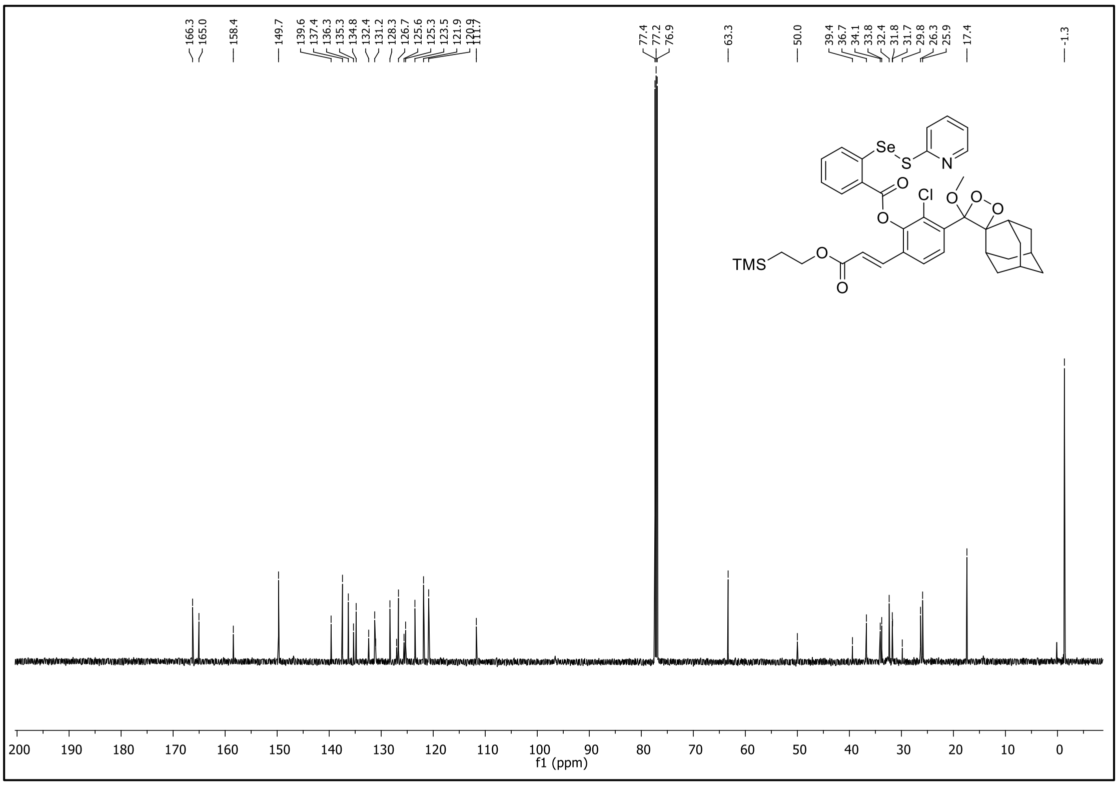

MS of compound **9:**

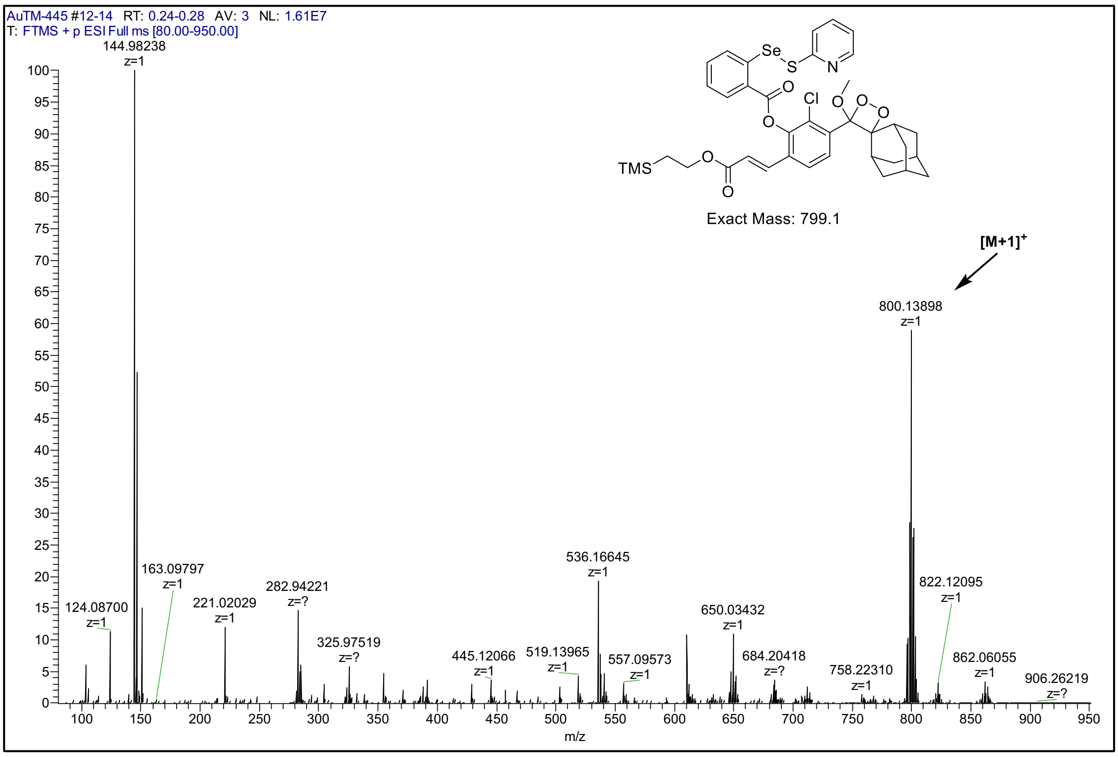

^{1}H-NMR and ^{13}C-NMR spectra of **probe 2:**

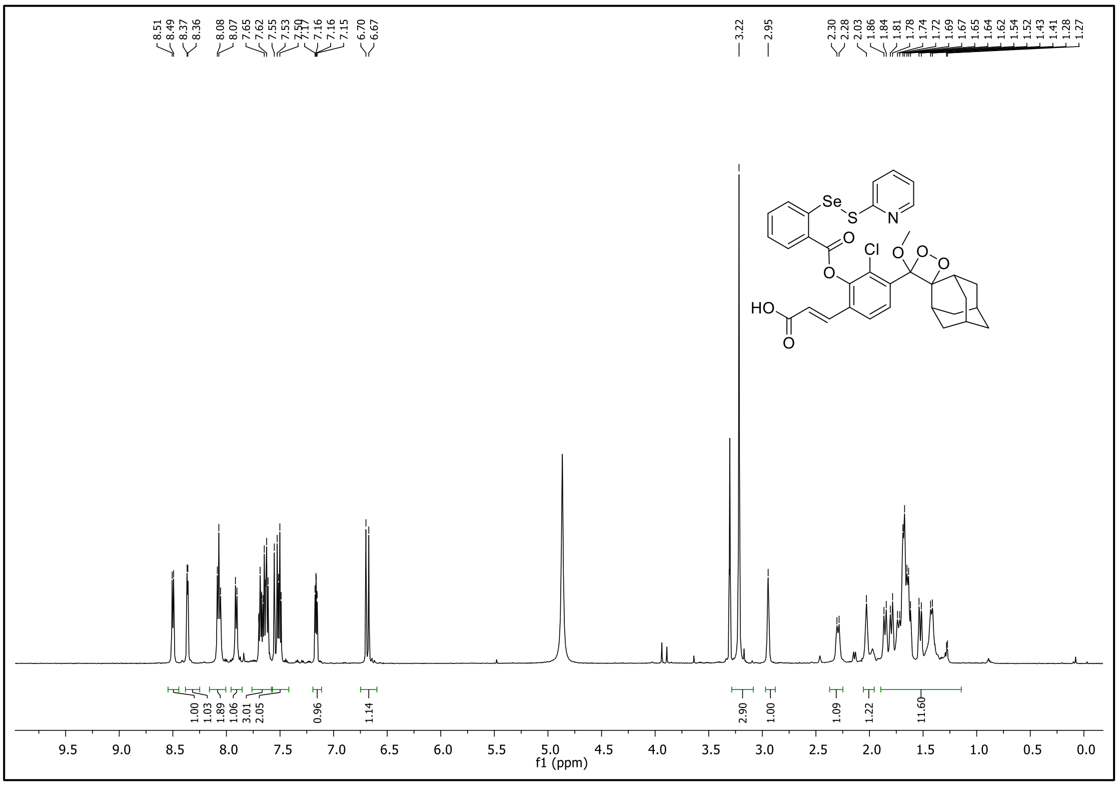

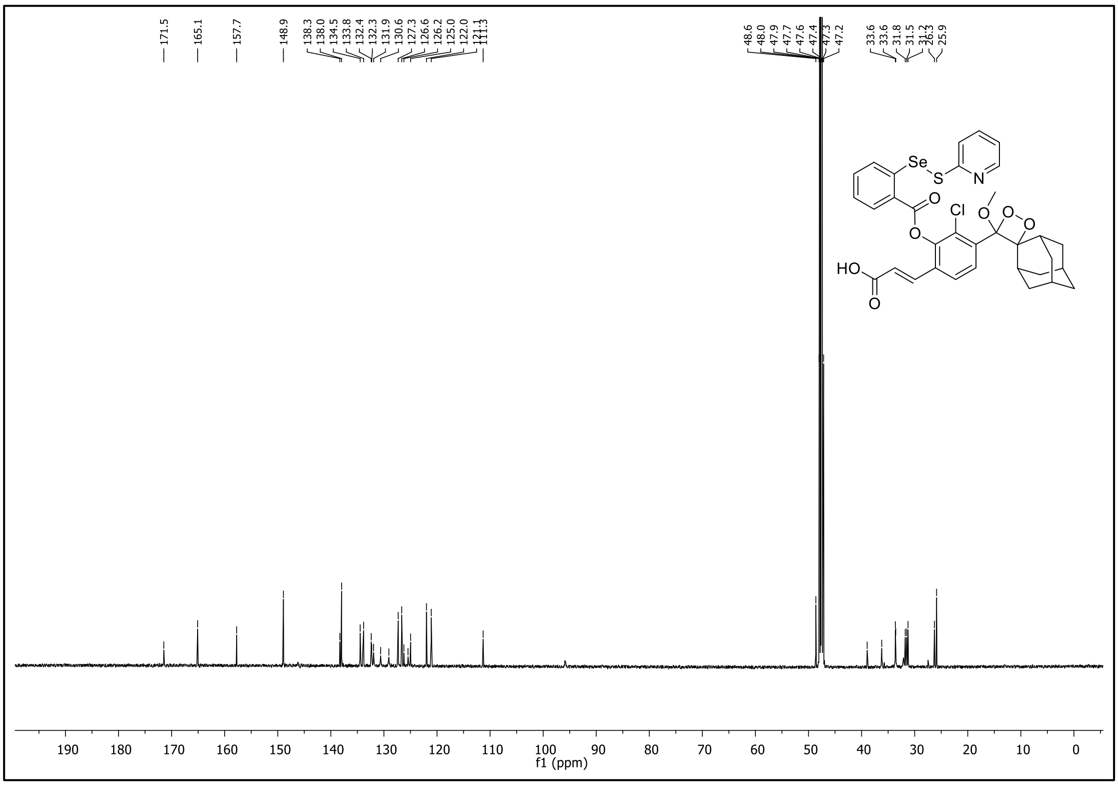

MS of **probe 2:**

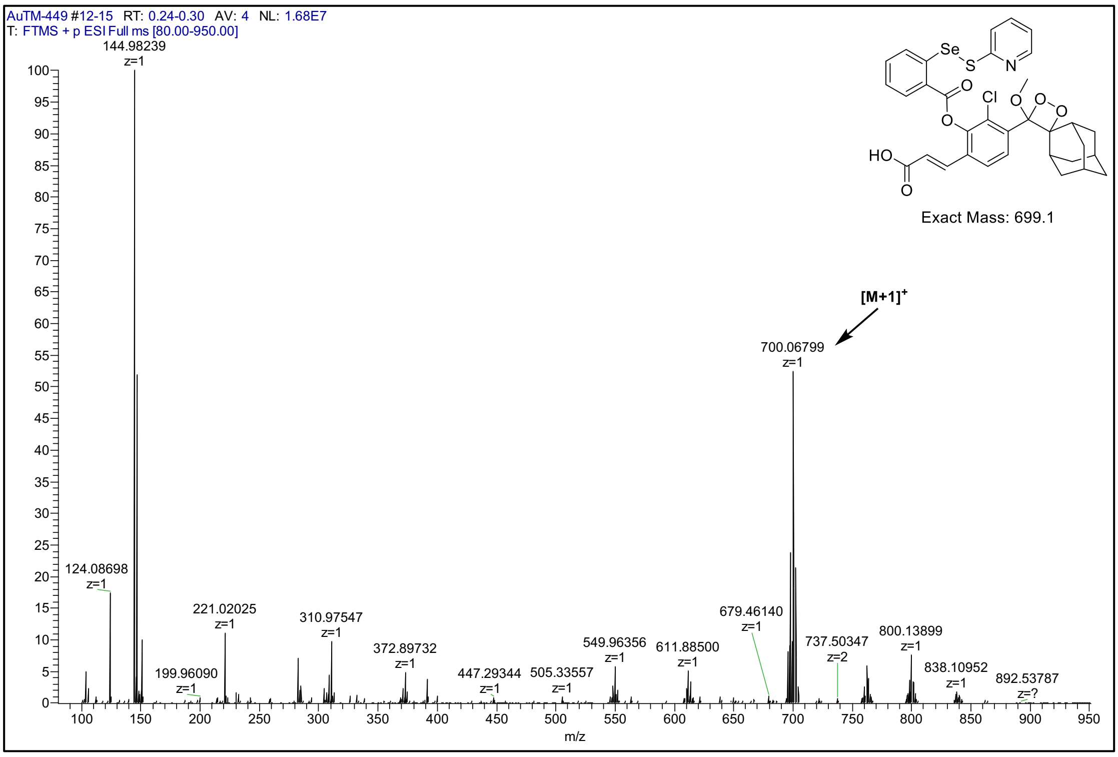

AuTM-449 #12-15 RT: 0.24-0.30 AV: 4 NL: 1.68E7
T: FTMS + p ESI Full ms [80.00-950.00]
144.98239
z=1
124.08698
z=1
199.96090
z=1
221.02025
z=1
310.97547
z=1
372.89732
z=1
447.29344
z=1
505.33557
z=1
549.96356
z=1
611.88500
z=1
679.46140
z=1
700.06799
z=1
737.50347
z=2
800.13899
z=1
838.10952
z=1
892.53787
z=?
[M+1]+
m/z
Se
S
N
O
O
Cl
O
O
O
HO
O
Exact Mass: 699.1

^{1}H-NMR and ^{13}C-NMR spectra of compound **12:**

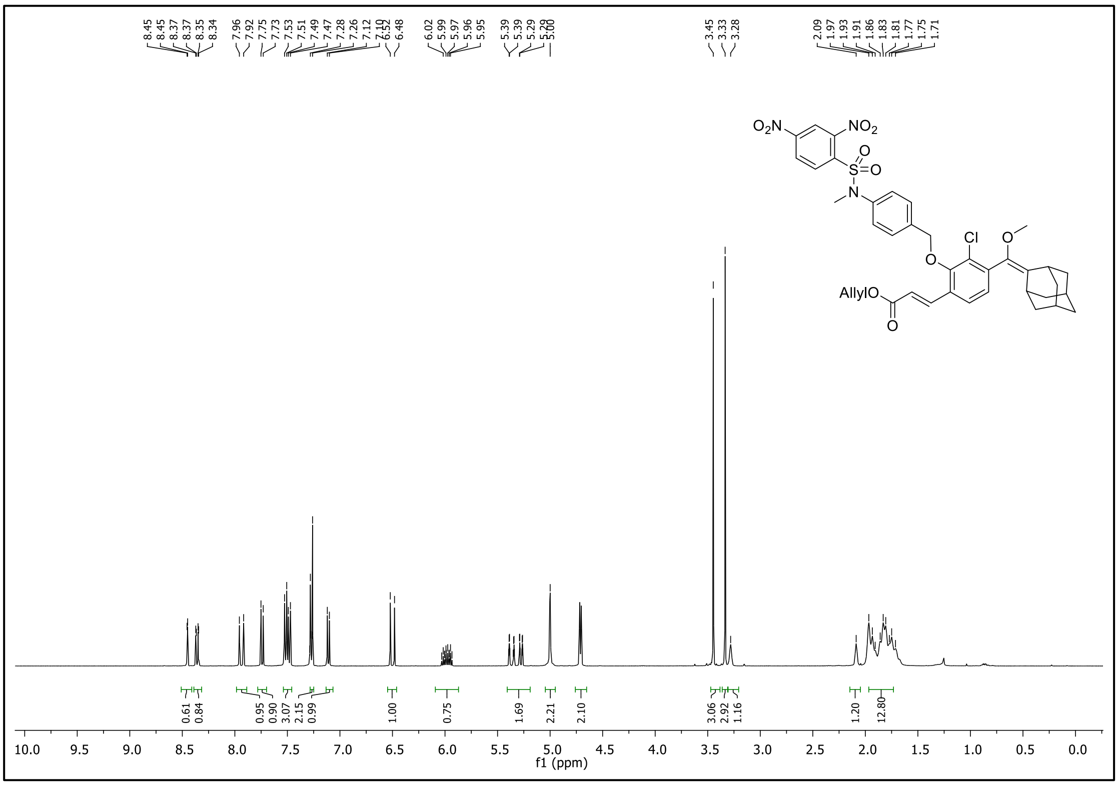

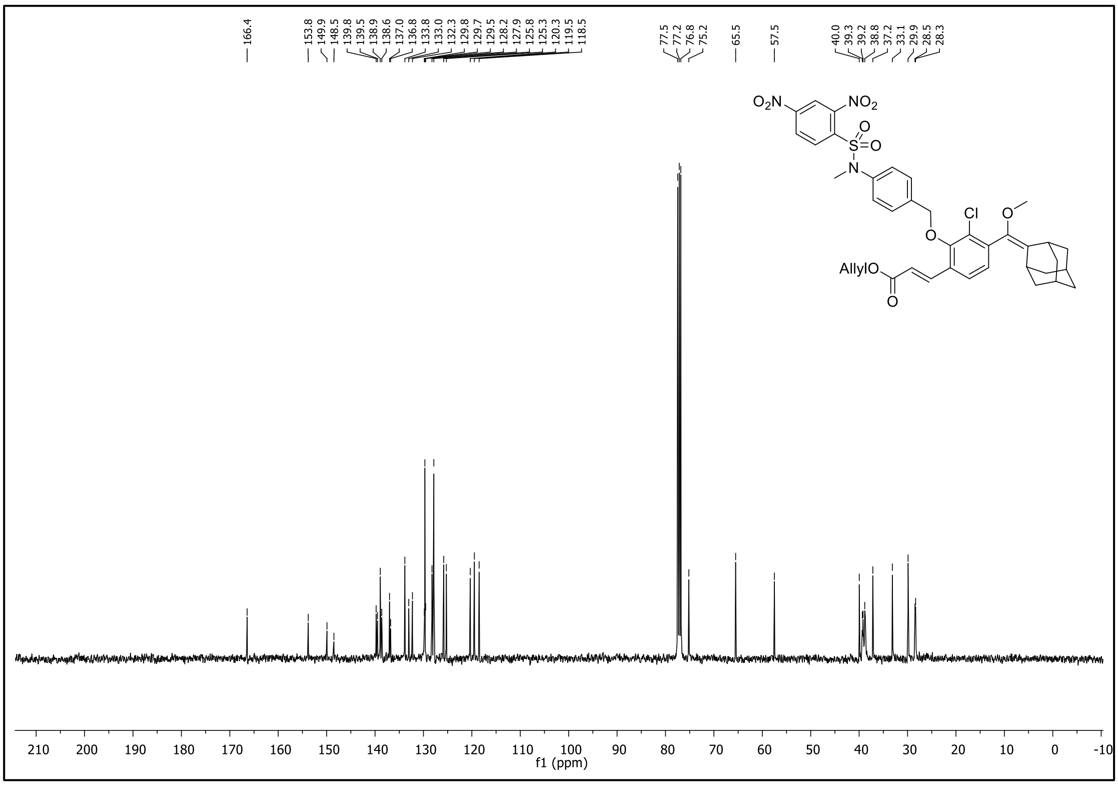

MS of compound **12:**

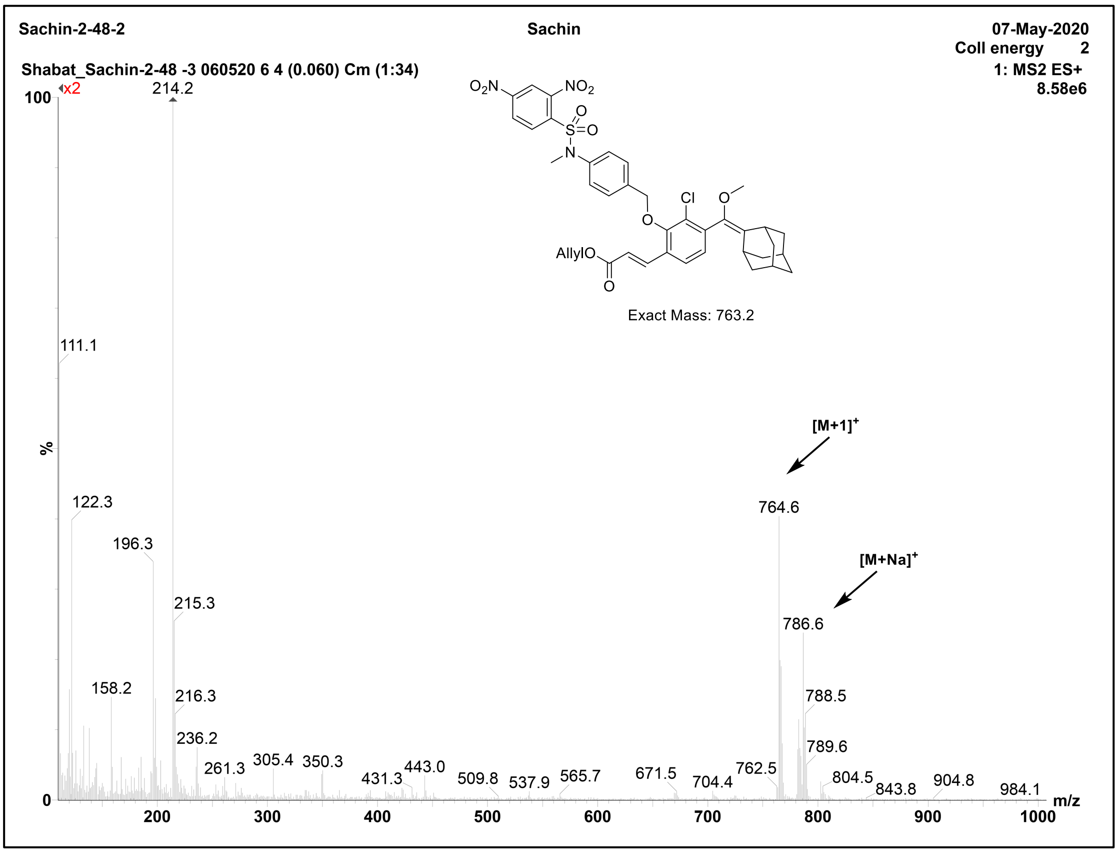

^{1}H-NMR and ^{13}C-NMR spectra of compound **13:**

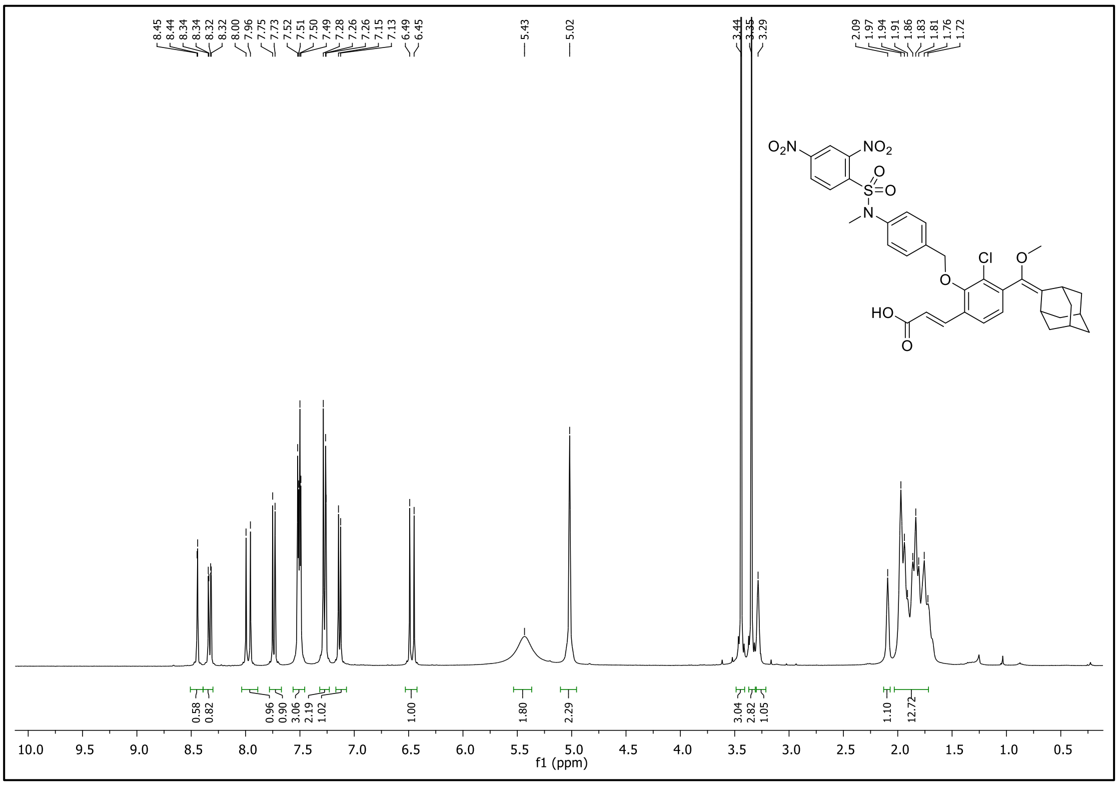

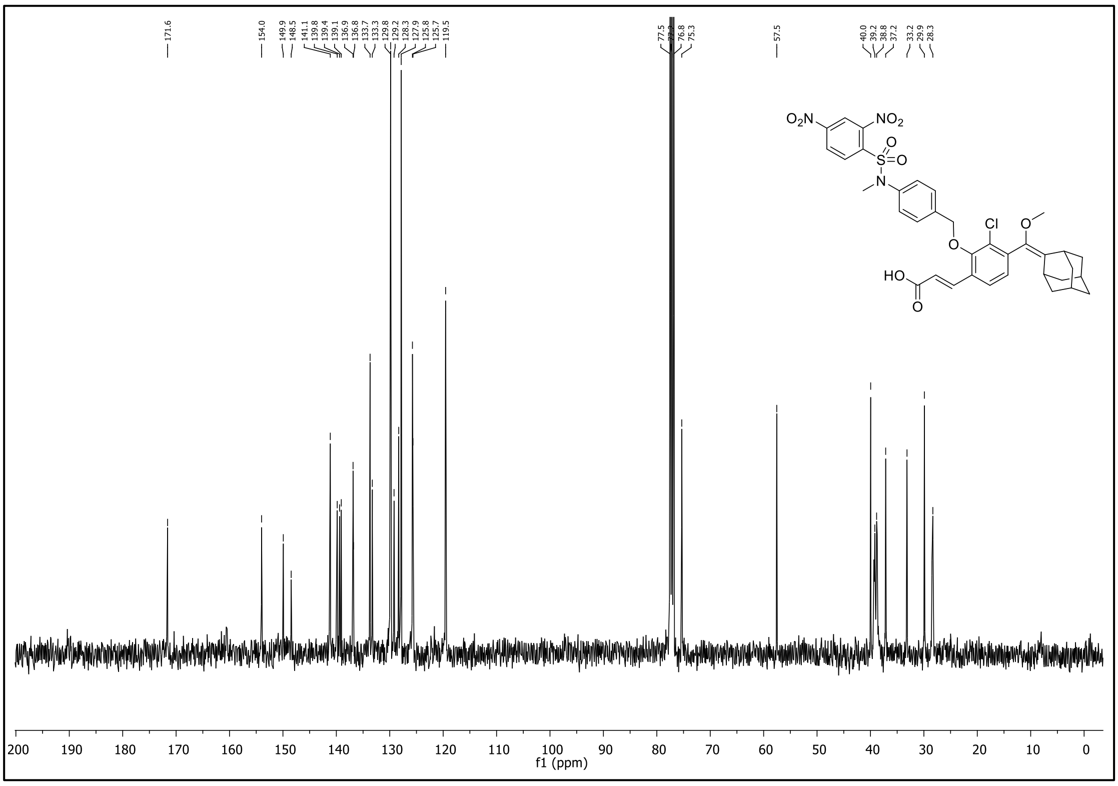

MS of compound **13:**

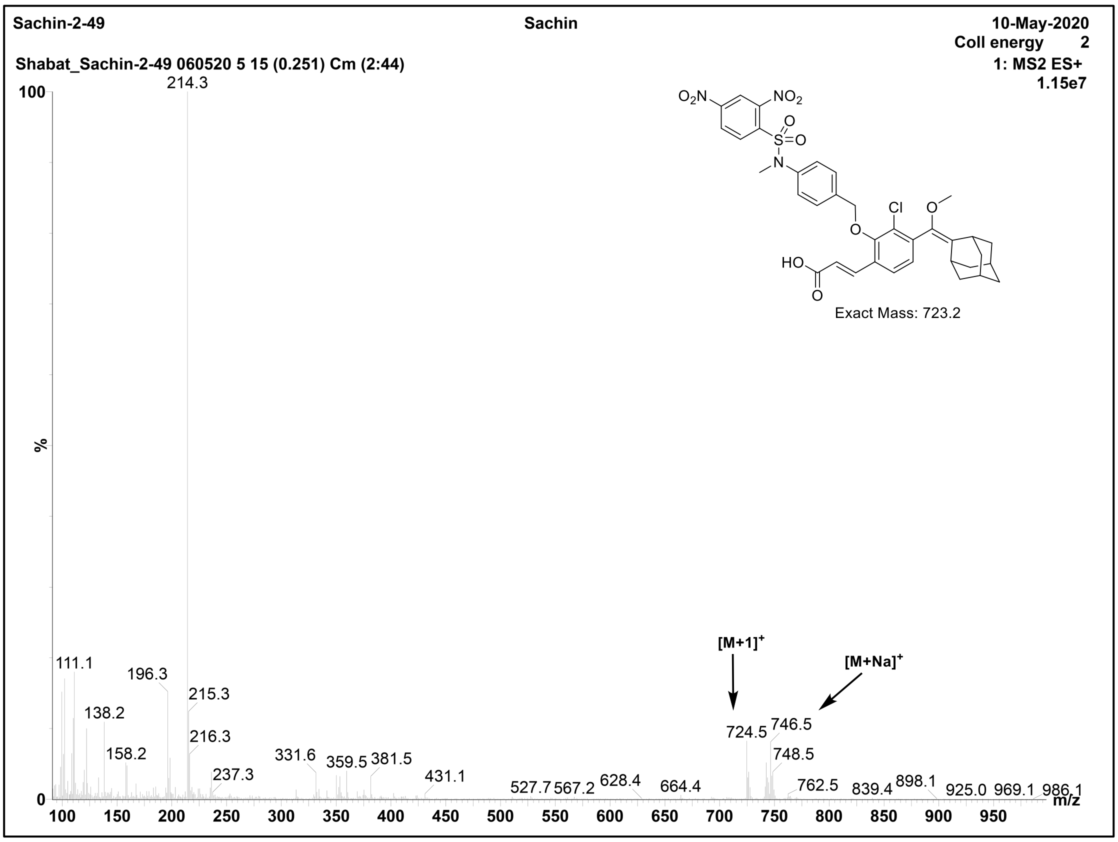

^{1}H-NMR and ^{13}C-NMR spectra of **probe 3:**

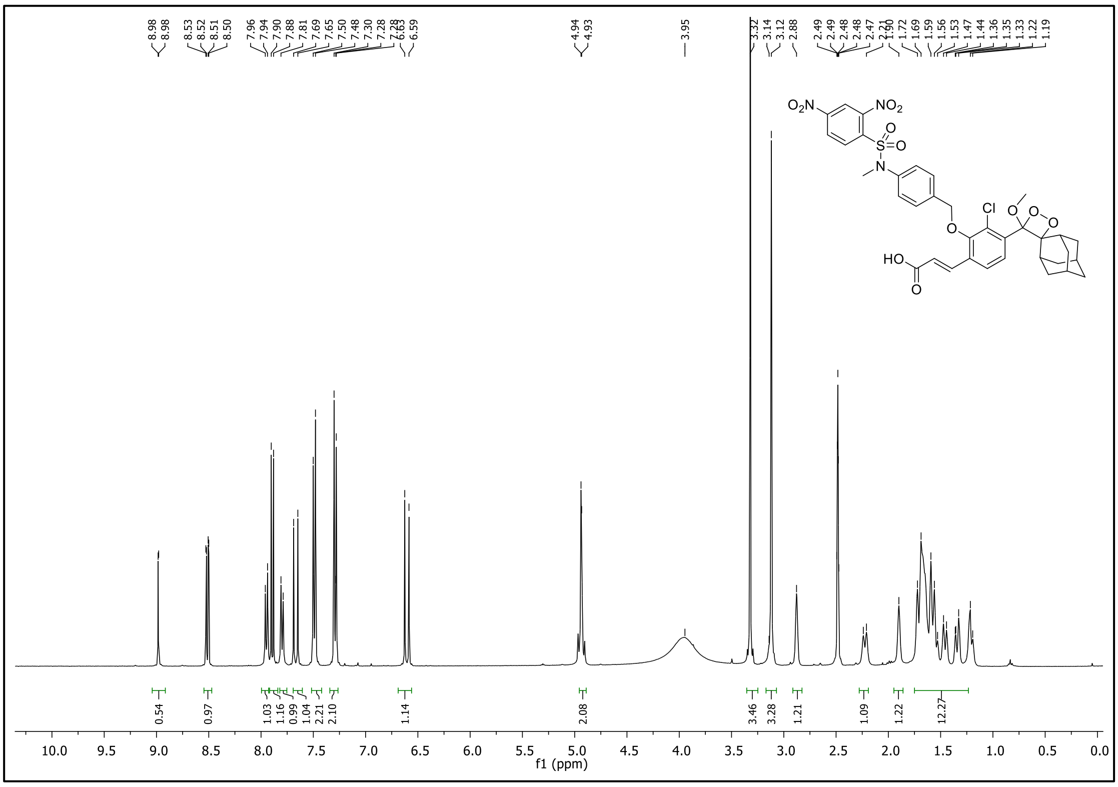

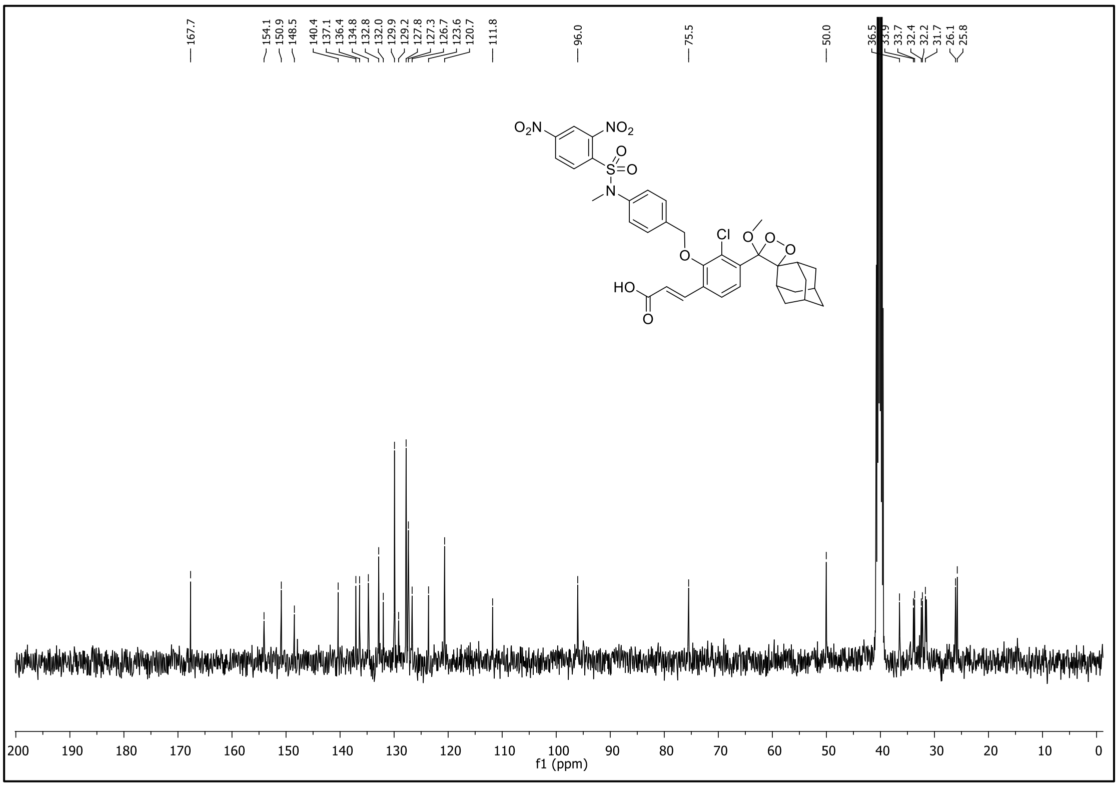

MS of **probe 3:**

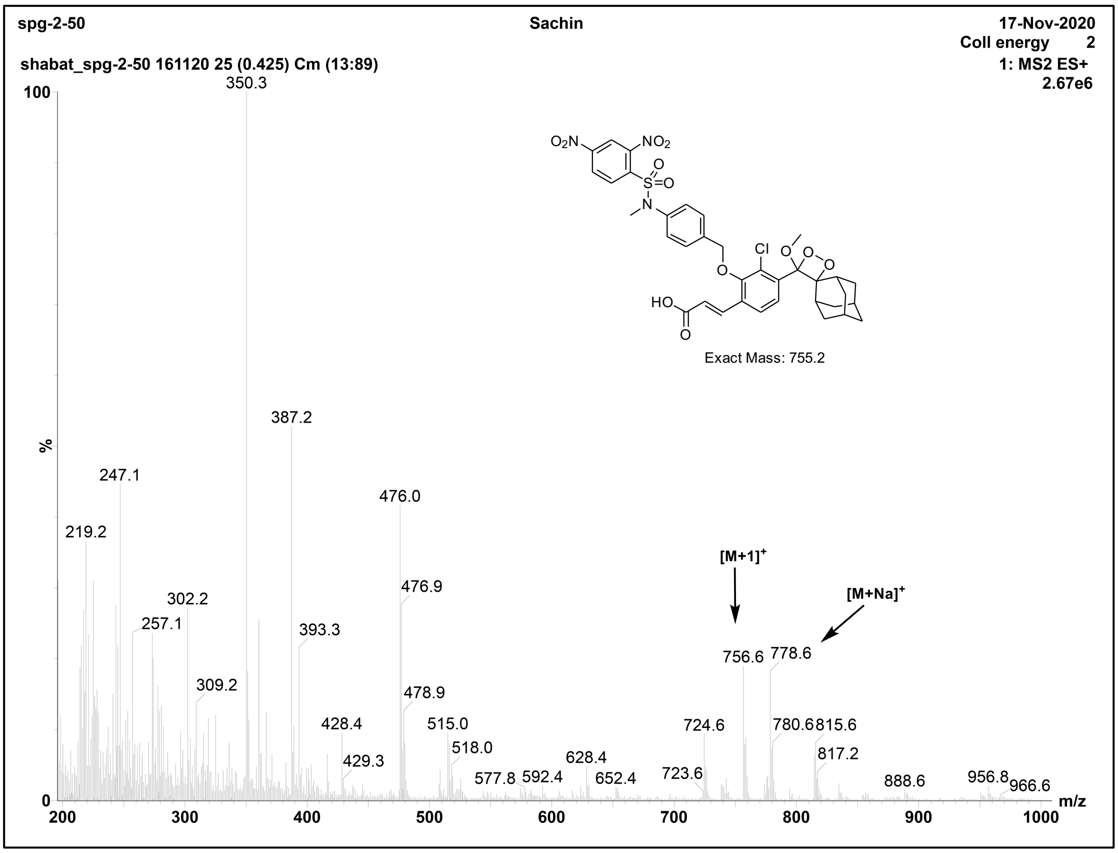
spg-2-50
Sachin
17-Nov-2020
Coll energy 2
shabat_spg-2-50 161120 25 (0.425) Cm (13:89)
1: MS2 ES+
2.67e6
100
%
0
350.3
387.2
247.1
219.2
476.0
476.9
302.2
257.1
393.3
309.2
478.9
428.4
515.0
518.0
429.3
577.8
592.4
628.4
652.4
723.6
724.6
[M+1]+
[M+Na]+
756.6
778.6
780.6
815.6
817.2
888.6
956.8
966.6
Exact Mass: 755.2
m/z
200
300
400
500
600
700
800
900
1000

^{1}H-NMR and ^{13}C-NMR spectra of **probe 4:**

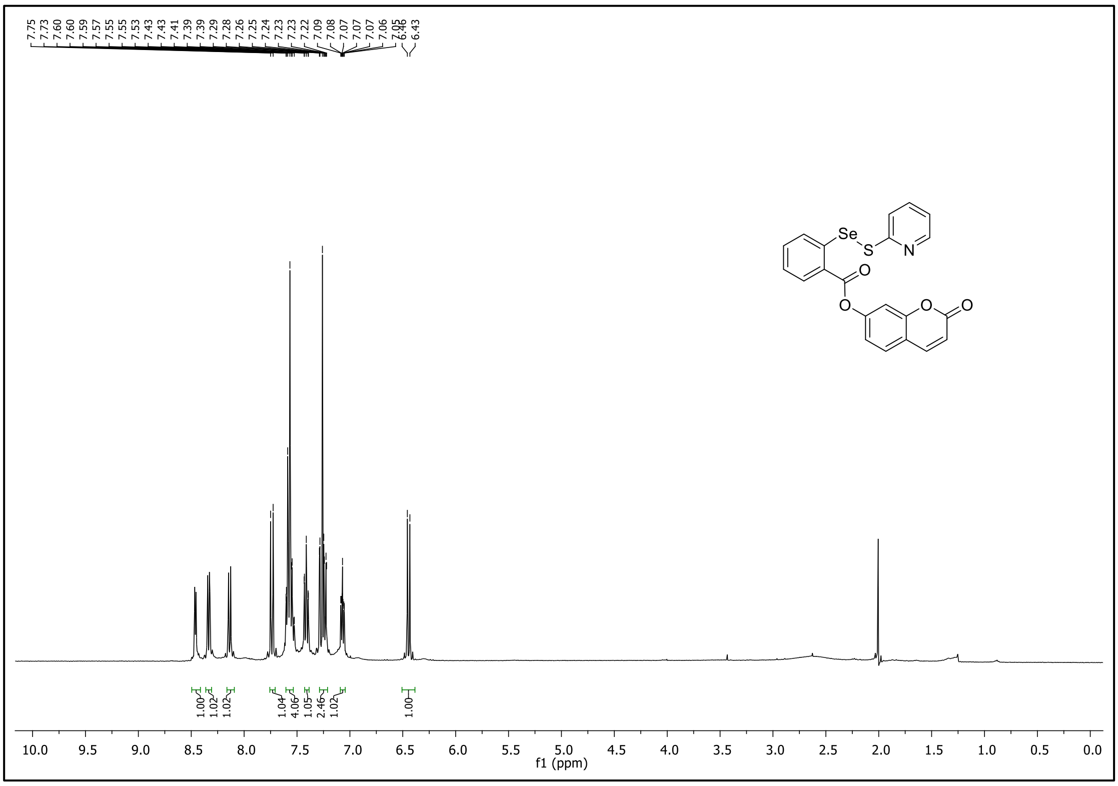

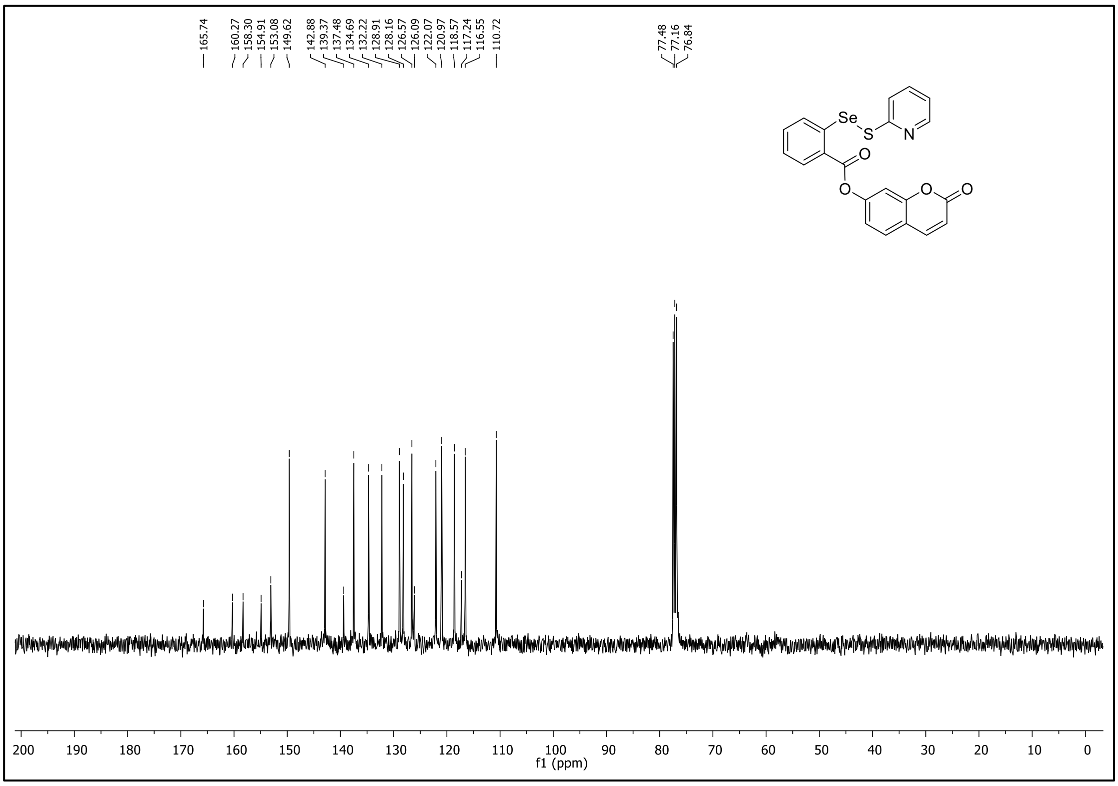

MS of **probe 4:**

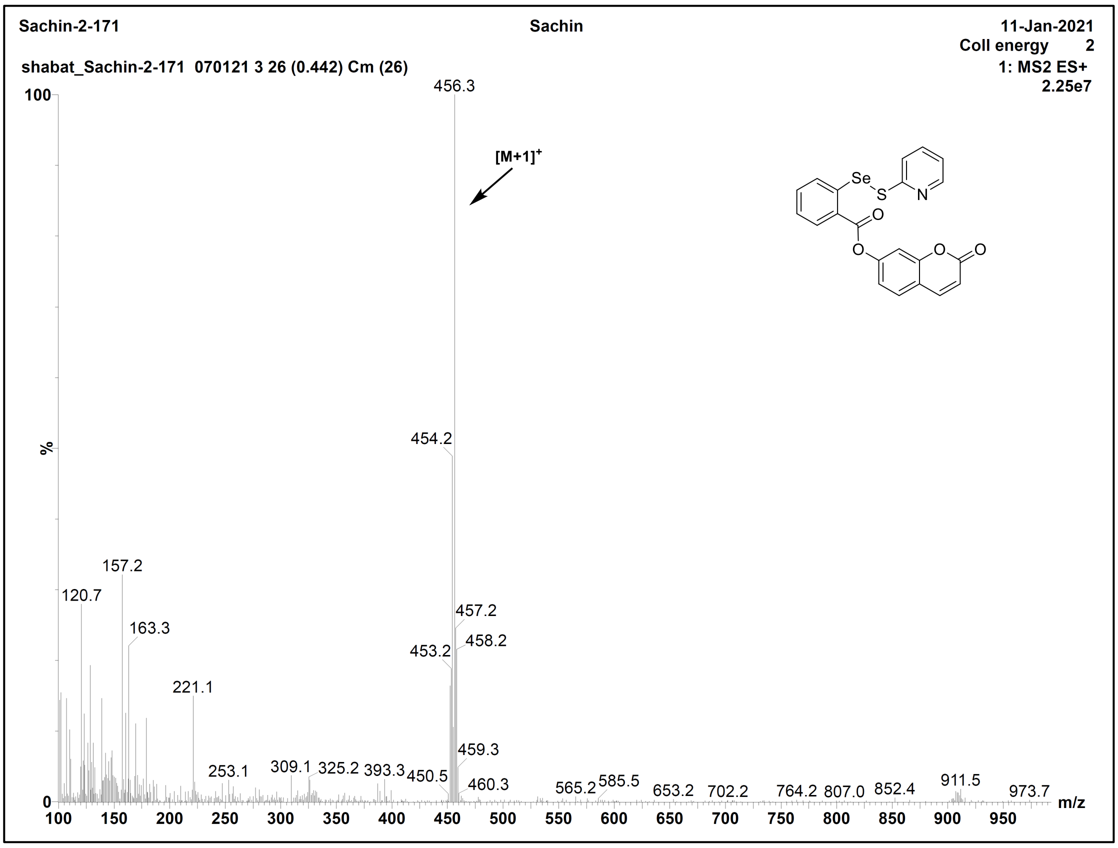
Sachin-2-171
Sachin
11-Jan-2021
Coll energy 2
shabat_Sachin-2-171 070121 3 26 (0.442) Cm (26)
1: MS2 ES+
2.25e7
100
%
0
456.3
[M+1]+
454.2
157.2
120.7
163.3
457.2
458.2
453.2
221.1
459.3
253.1
309.1
325.2
393.3
450.5
460.3
565.2
585.5
653.2
702.2
764.2
807.0
852.4
911.5
973.7
m/z
100 150 200 250 300 350 400 450 500 550 600 650 700 750 800 850 900 950
Se
S
N
O
O
O
O

5. References:

1) Hananya, N.; Green, O.; Blau, R.; Satchi-Fainaro, R.; Shabat, D. A Highly Efficient Chemiluminescence Probe for the Detection of Singlet Oxygen in Living Cells. *Angew. Chem. Int. Ed.,* **2017**, *56*, 11793-11796.
2) Men, J.; Yang, X.; Zhang, H.; Zhou, J. A Near-Infrared Fluorescent Probe Based on Nucleophilic Substitution-Cyclization for Selective Detection of Hydrogen Sulfide and Bioimaging. *Dyes Pigm.,* **2018**, *153*, 206-212.
3) Hananya, N., Reid, J. P., Green, O., Sigman, M. S., Shabat, D. Rapid Chemiexcitation of Dioxetane-Based Luminophores Yields Ultrasensitive Chemiluminescence Assays. *Chem. Sci*., **2019**, *10*, 1380-1385.
4) Duvall, J. R.; Wu, F.; Snider, B. B. Structure Reassignment and Synthesis of Jenamidines A1/A2, Synthesis of (+)-NP25302, and Formal Synthesis of SB-311009 Analogues. *J. Org. Chem.,* **2006**, *71*, 8579-8590.
5) Chen, W.; Xu, S.; Day, J. J.; Wang, D.; Xian, M. A General Strategy for Development of Near-Infrared Fluorescent Probes for Bioimaging. *Angew. Chem. Int. Ed.,* **2017**, *56*, 16611-16615.
6) Redy-Keisar, O.; Kisin-Finfer, E.; Ferber, S.; Satchi-Fainaro, R.; Shabat, D. Synthesis and Use of QCy7-Derived Modular Probes for the Detection and Imaging of Biologically Relevant Analytes. *Nat. Protoc.,* **2014**, *9*, 27-36.

6. APPENDIX

Explanation why 1st group triggers the probe

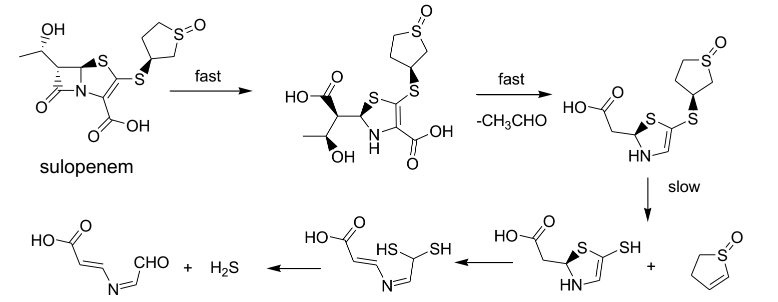

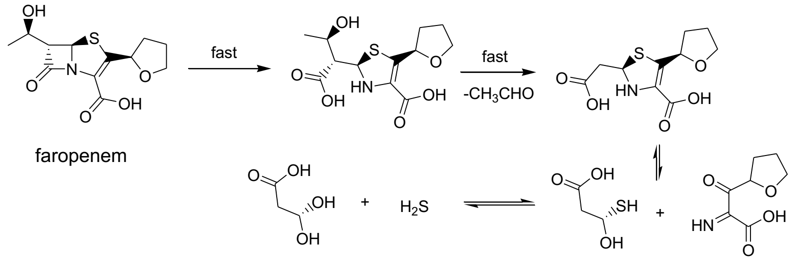

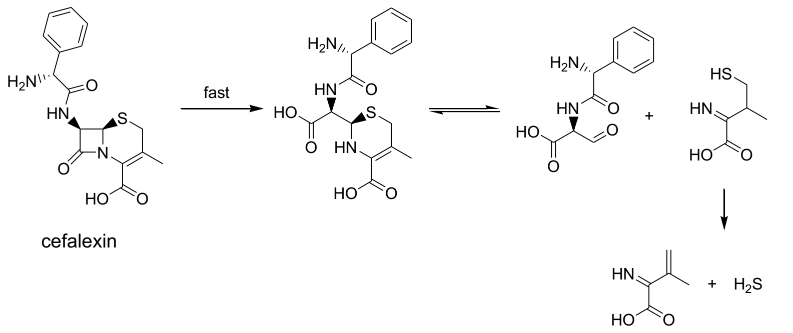

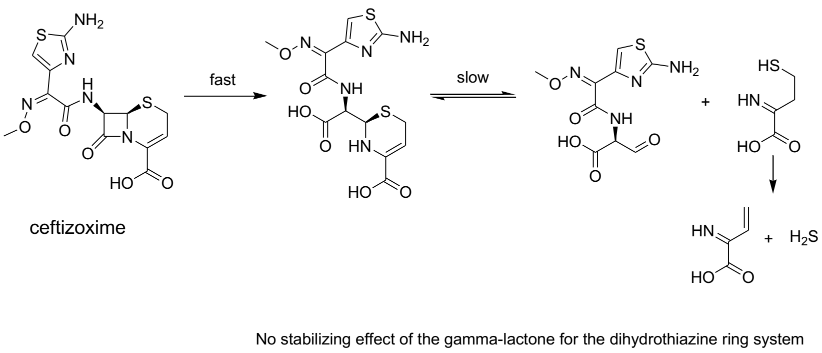

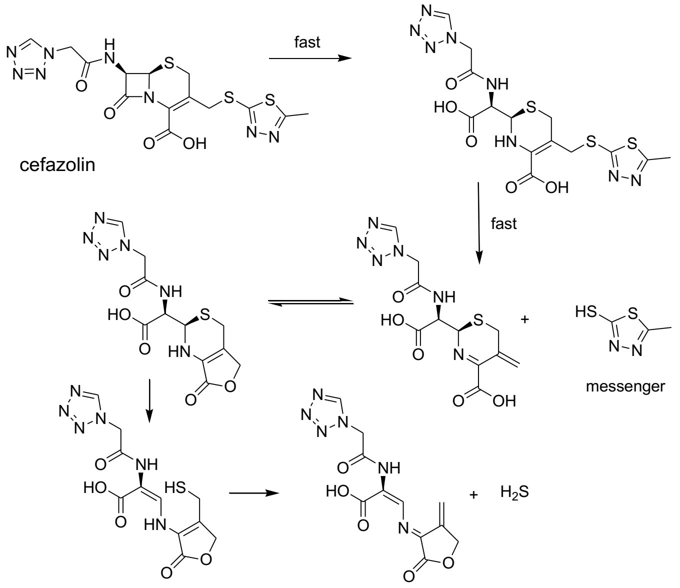

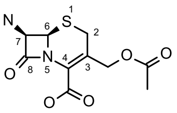

Figure 1 Established position numbering for cephems

Explanation why 2nd group does not trigger the probe

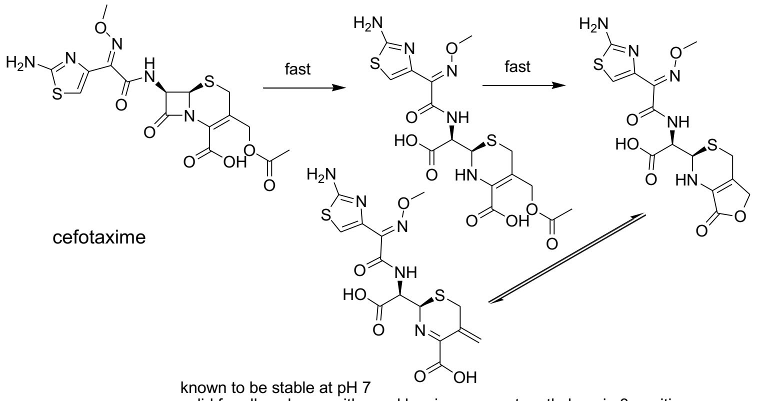

known to be stable at pH 7
valid for all cephems with good leaving group at methylene in 3-position

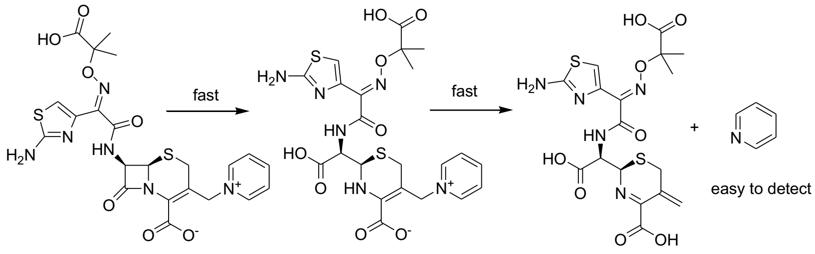

ceftazidime

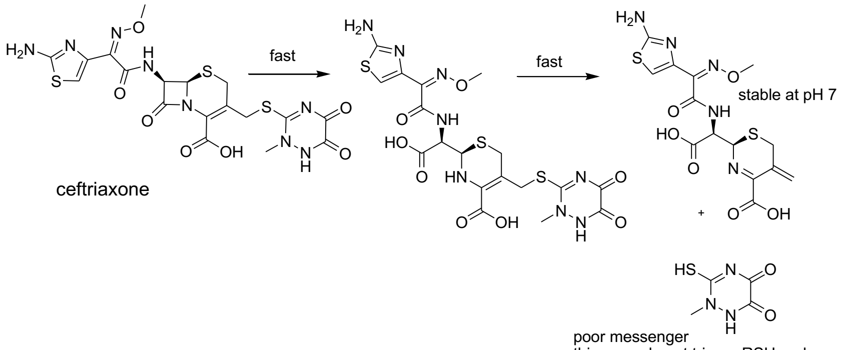

poor messenger
thioureas do not trigger RSH probes

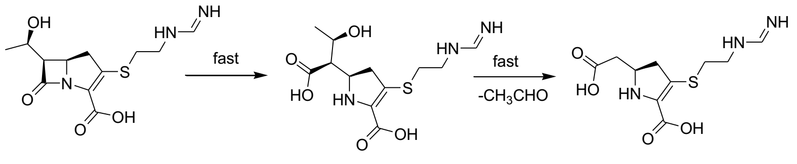

imipenem

very stable structure free of acetal-like functionality

LCMS method description

All samples were measured on Agilent 1260 system with single quadrupole MSD featured with multimode (ESI+APCI) ionization chamber. The ionization chamber parameters were as follows:

a) positive mode scan signal: ionization mode MM-ES+APCI; fragmentor ramp enabled; percent cycle time 100.00 %; mass range 40-900 m/z; fragmentor variable with ramp: mass 121.05 value 30, mass 922.01 value 150; sheath gas temperature 200°C; vaporizer temperature 180°C, gas flow 7L/min; nebulizer pressure 3105 Torr; quadrupole temperature 100°C, capillary voltage 3000V; charge voltage 1500V; corona current 3 µA.

b) negative mode scan signal: ionization mode MM-ES+APCI; fragmentor ramp enabled; percent cycle time 100.00 %; mass range 40-900 m/z; fragmentor variable with ramp: mass 121.05 value 100, mass 922.01 value 300; sheath gas temperature 200°C; vaporizer temperature 180°C, gas flow 7L/min; nebulizer pressure 3105 Torr; quadrupole temperature 100°C, capillary voltage 4000V; charge voltage 2000V; corona current 40 µA.

The HPLC part of the device comprised a quadruple gradient pump, an headspace autosampler, Phenomenex Luna C8 5µm, 4.6x150 mm column and a DAD detector at 254 nm. The acidic elution method was a combination of 7 min gradient from 5% to 100% MeOH in 0.1% formic acid and subsequent 8min elution with pure MeOH. The acidic elution was used for both polarities as the beta-lactams open spontaneously under basic conditions.

Sample preparation for the LCMS assay

All antibiotics were purchased from commercial suppliers (sulopenem, cetazidime, imipenem, ceftizoxime from Biosynth-Carbosynth EN, the rest from Merck-Aldrich). A 10 mM stock solutions of inspected antibiotics in PBS (prepared by dissolving Merck-Aldrich P3813-1PAK in RODI water and set to 1L total volume) were made. Beta lactamase enzyme (*Bacillus cereus* 569/H9 from Aldrich, 426205-500U) stock solution was prepared by dissolving 1mg of dry enzyme in PBS and set by PBS to total volume of 1mL. Every LCMS sample was made by mixing 0.25 mL of antibiotic stock solution with 0.25mL of lactamase stock solution in a 2mL head space vial and incubation at RT (45min on linear shaker, 60 shakes per minute). 50 µL of the resulting solution was directly injected to LCMS after a period specified later. Stock solutions of ATB were directly injected in 10 µL loads to acquire data of starting material standards.

Faropenem decay promoted by B. Cereus metallo-lactamase, assayed by LCMS

The analysis with negative ionization was captured directly after the incubation period (injected after 45 min of incubation), positive ionization experiment started after 90 min of incubation and negative ionization experiment was repeated with identical result after 110 min of incubation.

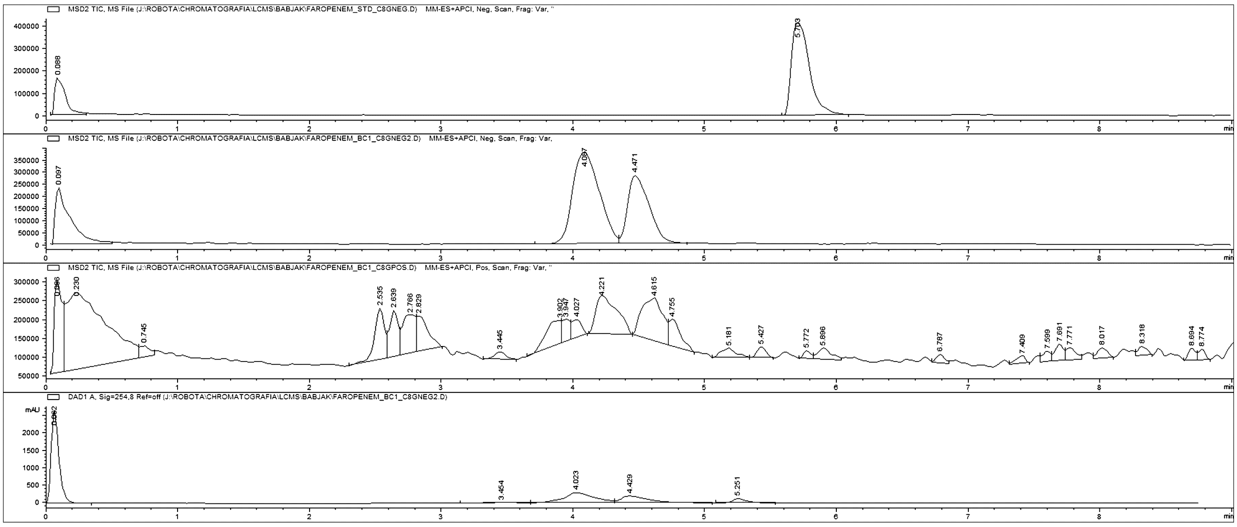

Faropenem had hydrolysed completely during 45 min, furnishing 2 major peaks eluting at 4.08 and 4.47 min, 2 minor UV active peaks at 3.45 and 5.25 min (having only positive MS signal) and one large positive peak at 2.54 min with no UV absorption, which belongs to 2-amino-2-(hydroxymethyl)-1,3-propanediol (m/z 122.1 in positive mode), the key part of TRIS buffer in which was the enzyme lyophilised by manufacturer (this peak is notable in all presented LCMS data and won´t be commented again). The two major peaks had almost identical MS spectra in both polarities. Here we list the spectra for described peaks:

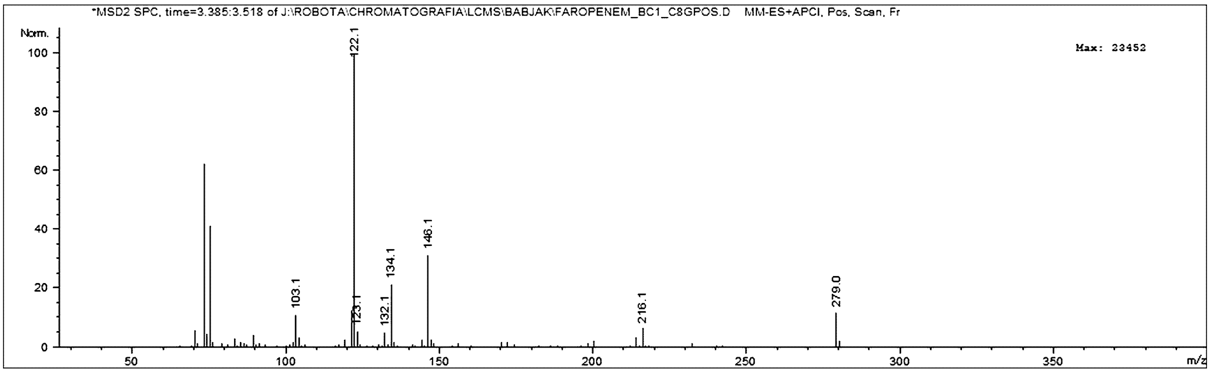

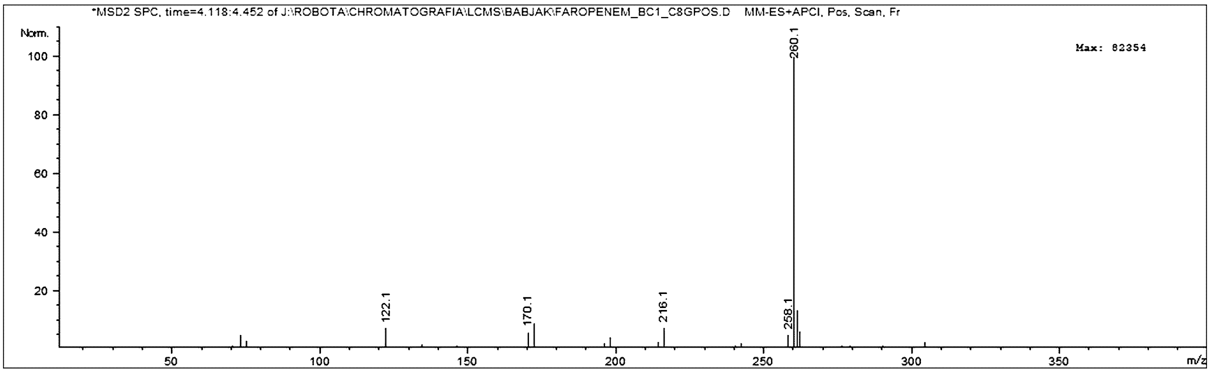

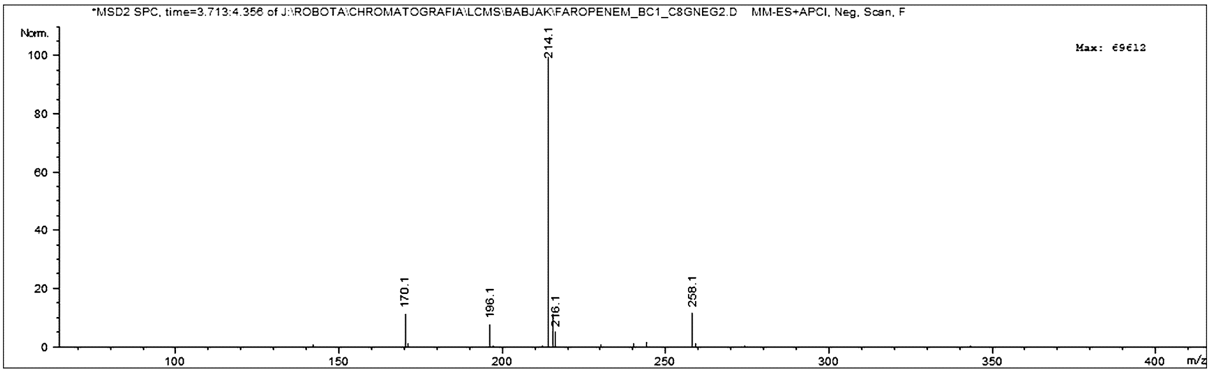

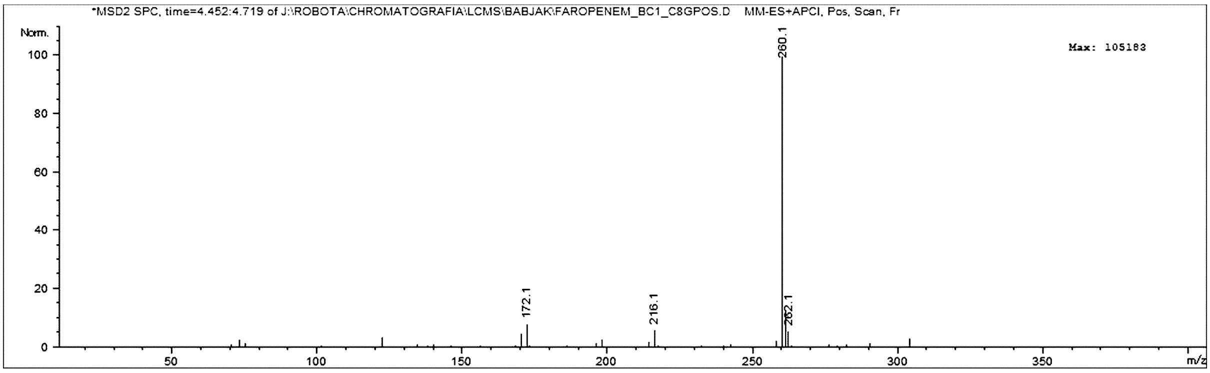

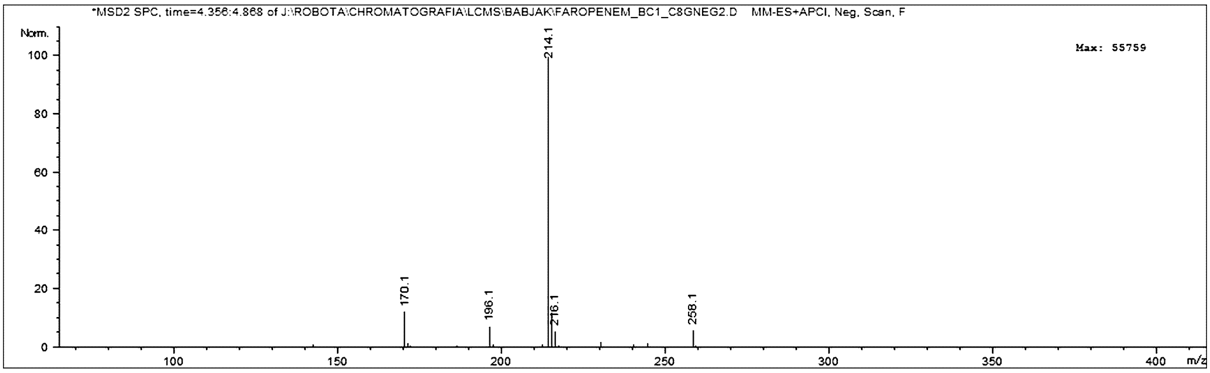

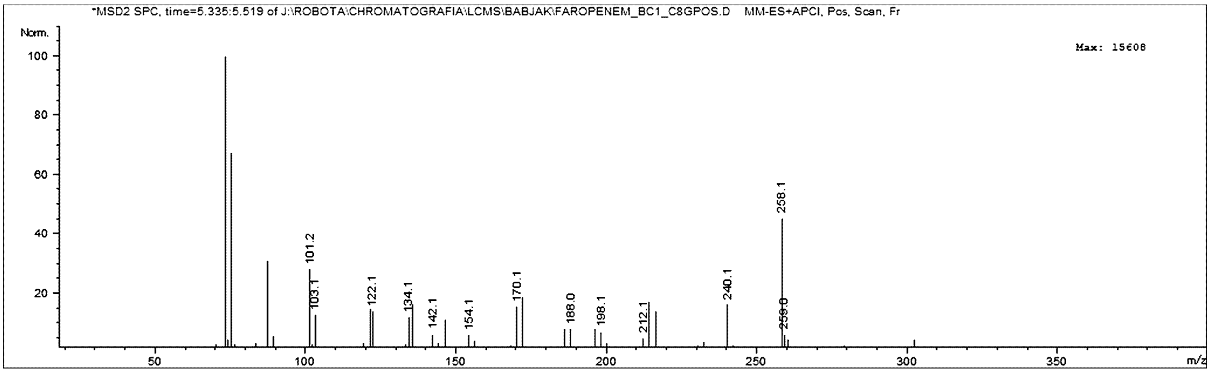

After inspecting all major ions detected, we have constructed major decomposition pathway for the faropenem. Major changes after opening the beta-lactam ring are a) decarboxylation, which is likely happening in the MS detector b) retro-aldol reaction.[1] Those major products are almost identical in its fragmentation and share almost the same molar weight. The proposed mechanism contains several

[1] Drawz, S. M.; Babic, M.; Bethel, C. R.; Taracila, M.; Distler, A. M.; Ori, C., … Bonomo, R. A. *Biochemistry* **2010,** 49(2), 329–340, doi:10.1021/bi9015988

intermediates with SH-group, capable to trigger a RSH sensing probe, and allows several ways to generate H_2S from outlined thioacetal and thiohemiaminal structures.

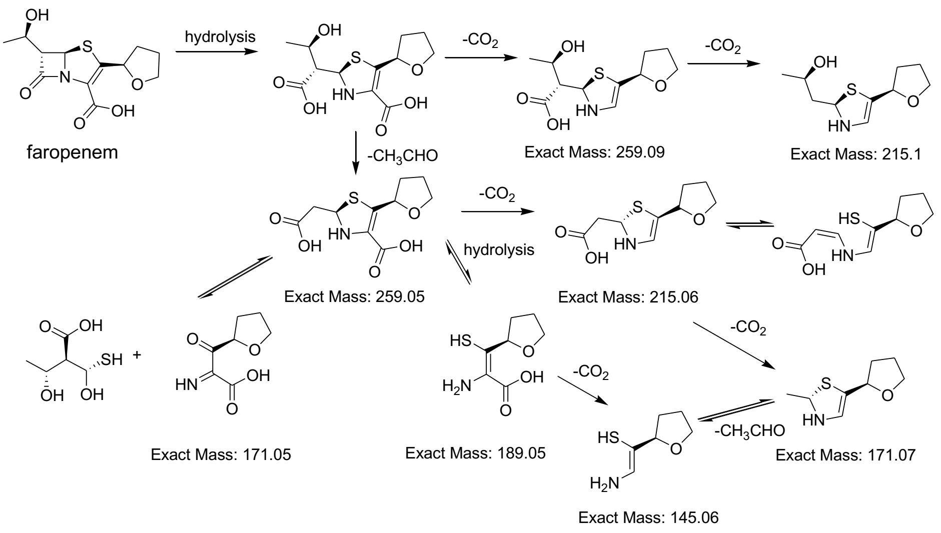

Scheme 1: Metalo-lactamase mediated decomposition pattern for faropenem.

Sulopenem decay promoted by B. Cereus metallo-lactamase, assayed by LCMS

The analysis with negative ionization was captured directly after the incubation period (injected after 45 min of incubation), positive ionization experiment started after 90 min of incubation and negative ionization experiment was repeated with identical result after 110 min of incubation. Sulopenem decomposed only partially during this period and had to incubate overnight (10h overall) to reach complete conversion. Following chromatogram shows the completed reaction mixture after 10 h.

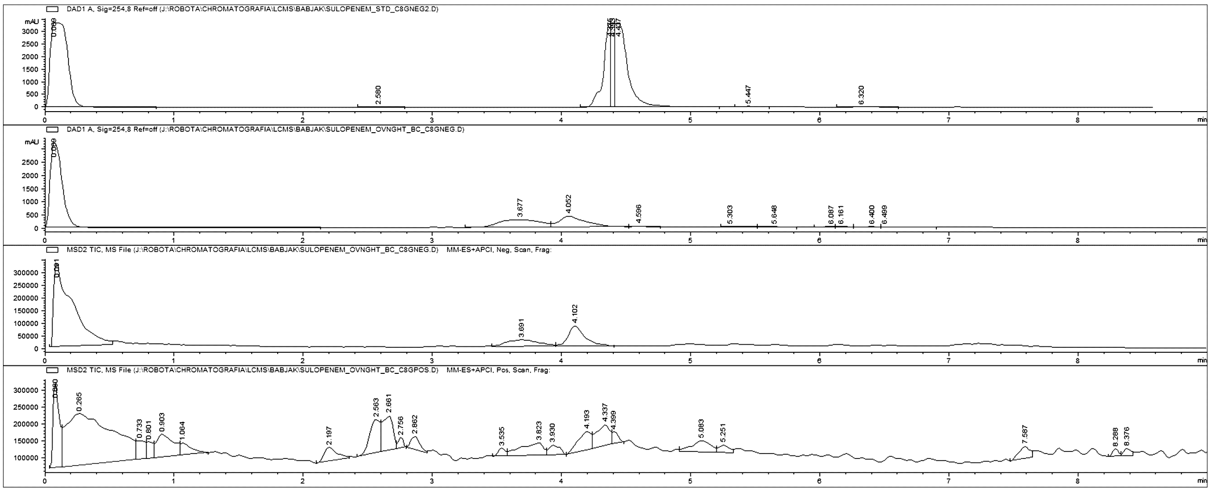

Two major UV-absorbing peaks were detected in the hydrolysed mixture at 3.68 an 4.05 min. Negative ionization signal provided only these two peaks. Positive signal was also collected at 2.54 min, originating from the TRIS amine (m/z 122.1).

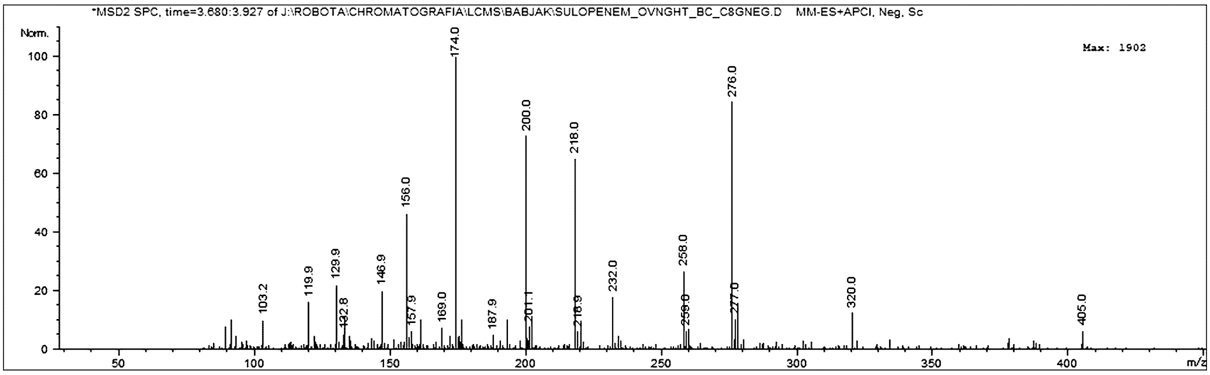

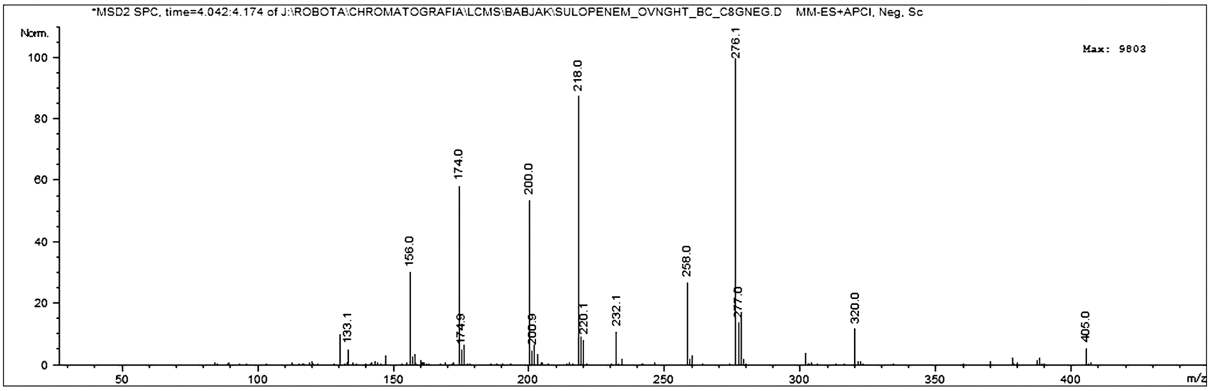

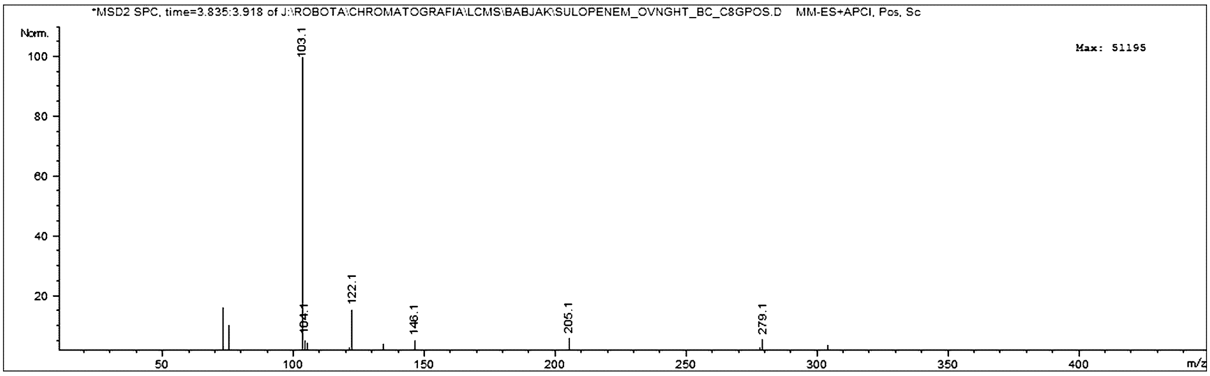

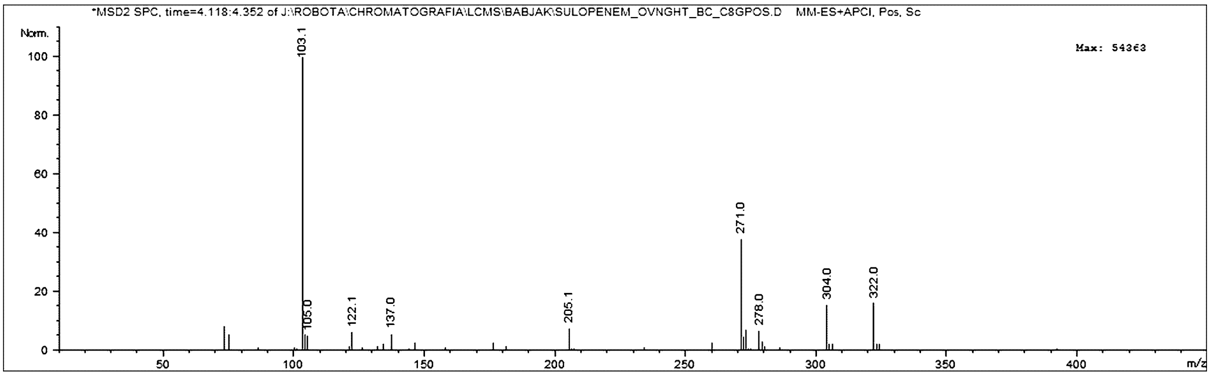

Proposed decomposition pathway is roughly like the one for faropenem. The greatest difference opens the explanation of high detection rate of this antibiotic by RSH sensing probes: all the key intermediates can eliminate 2,3-dihydrothiophene 1-oxide *via* retro-Michael pathway and products are thiols, capable of reaction with the RSH sensing probe. 2,3-Dihydrothiophene-1-oxide signal was the strongest ion in the positive ionization MS spectrum for both major peaks, which strongly supports our explanation.

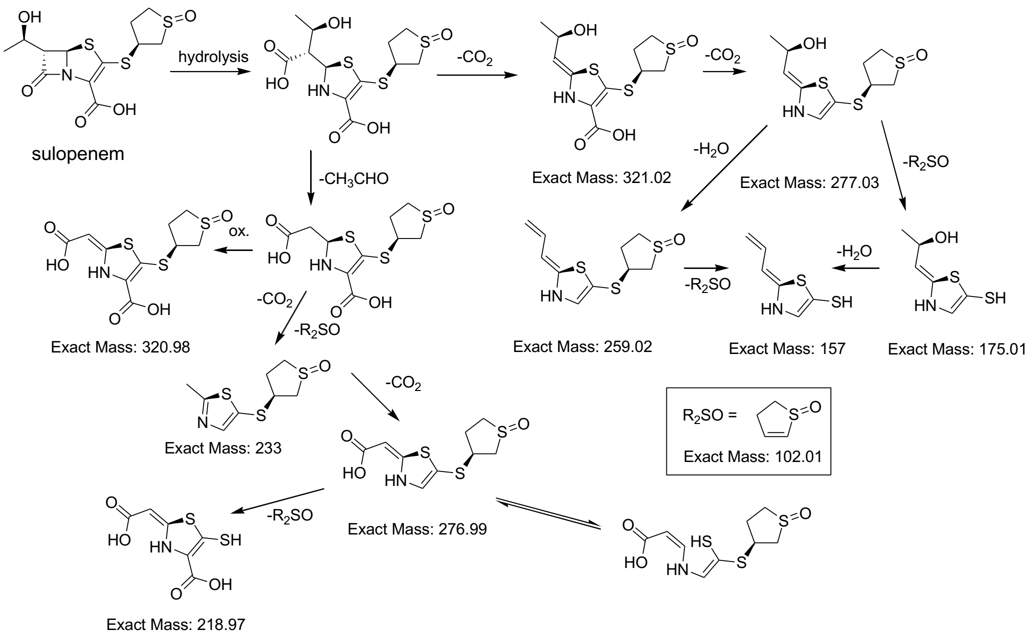

Penicillin V decay promoted by B. Cereus metallo-lactamase, assayed by LCMS

The analysis with negative ionization was captured directly after the incubation period (injected after 45 min of incubation at RT), positive ionization experiment started after 90 min of incubation. Penicillin V decomposed completely during this period. Only one major peak with higher polarity (6.51 min) was formed and remained untouched during the incubation. One minor peak was detected at 6.72 min. Fragment analysis is rather simple here: the hydrolysed beta lactam produces very stable compound, which then resists any further chemical changes. This is explaining very low luminescence of all tested beta-lactamase penicillin degradation mixtures with RSH sensing probe. The possible transformation through penillic acid, known from human metabolism is not likely in used reaction system and the key intermediate – the appropriate m/z for penillic acid - was not practically detected (the ion was present in very low concentration only). On the other hand, the ion with m/z 246 was developed in decent concentration, which speaks for good chance to produce H_2S through decarboxylation/elimination pathway at elevated temperatures. This process can occur in limited scale at RT and furnish small, but detectable H_2S levels, which then generated observed weak luminescent signal with H_2S sensitive probe.

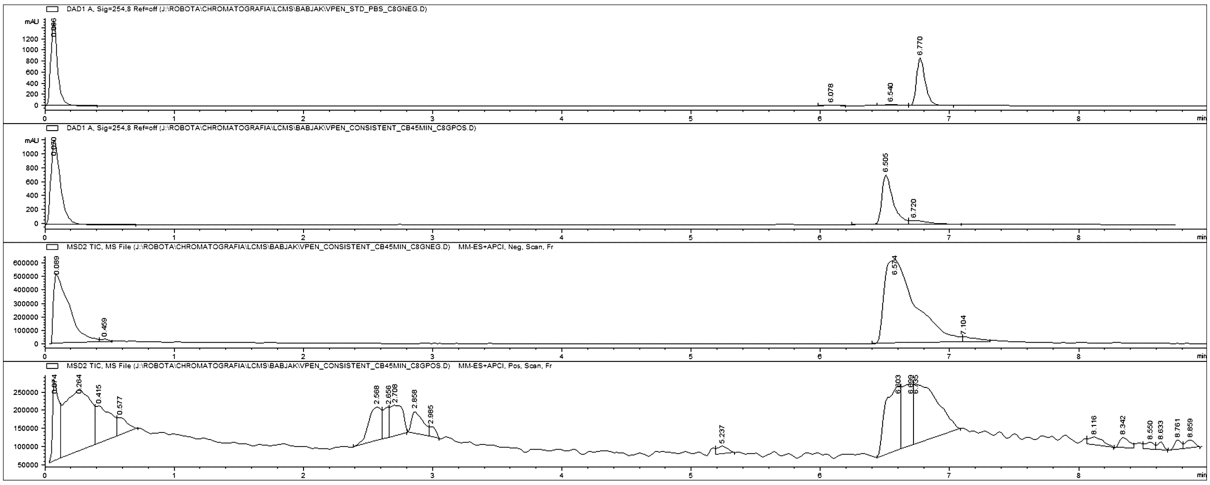
DAD1 A, Sig=254,8 Ref=off (J:\ROBOTA\CHROMATOGRAFIA\LCMS\BABJAK\VPEN_STD_PBS_C8GNEG.D)
6.770
DAD1 A, Sig=254,8 Ref=off (J:\ROBOTA\CHROMATOGRAFIA\LCMS\BABJAK\VPEN_CONSISTENT_CB45MIN_C8GPOS.D)
6.505
6.720
MSD2 TIC, MS File (J:\ROBOTA\CHROMATOGRAFIA\LCMS\BABJAK\VPEN_CONSISTENT_CB45MIN_C8GNEG.D) MM-ES+APCI, Neg, Scan, Fr
0.089
0.459
7.104
MSD2 TIC, MS File (J:\ROBOTA\CHROMATOGRAFIA\LCMS\BABJAK\VPEN_CONSISTENT_CB45MIN_C8GPOS.D) MM-ES+APCI, Pos, Scan, Fr
min

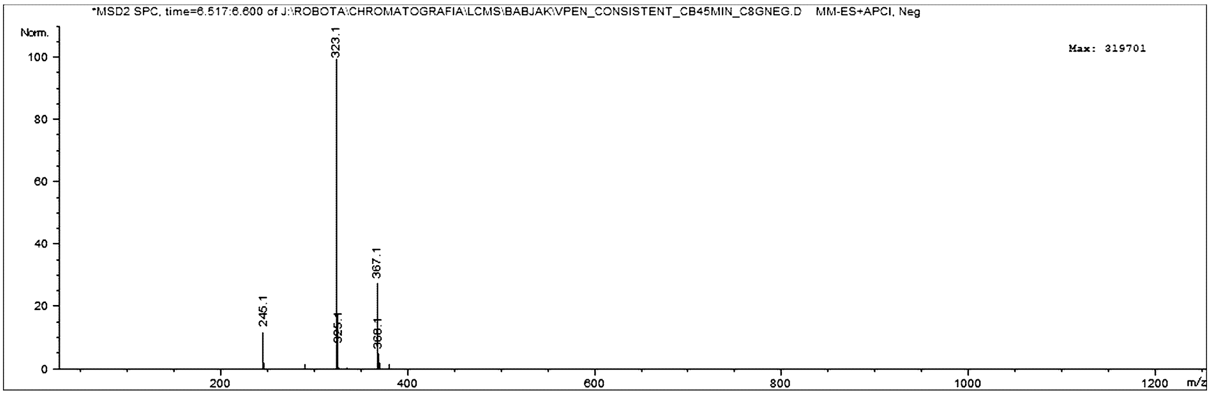
*MSD2 SPC, time=6.517:6.600 of J:\ROBOTA\CHROMATOGRAFIA\LCMS\BABJAK\VPEN_CONSISTENT_CB45MIN_C8GNEG.D MM-ES+APCI, Neg
Norm.
Max: 319701
323.1
367.1
245.1
325.1
368.1
m/z

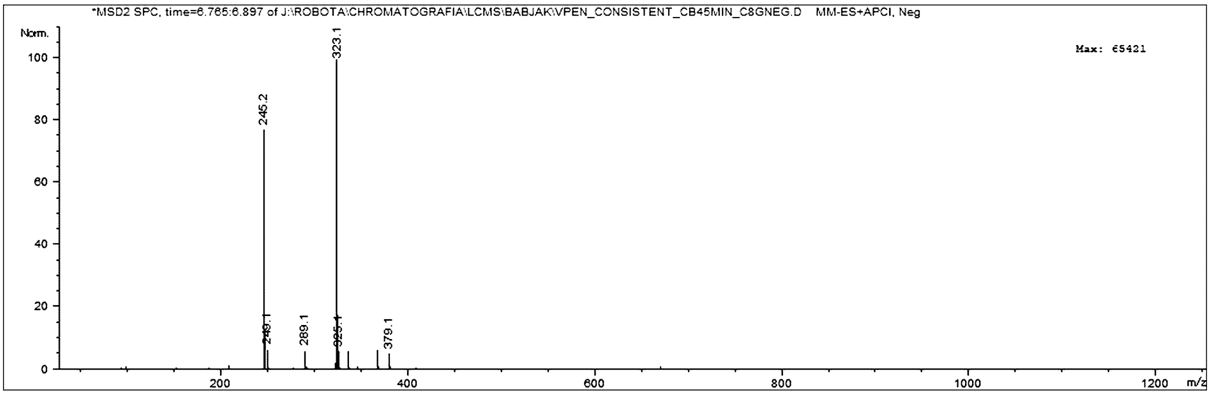
*MSD2 SPC, time=6.765:6.897 of J:\ROBOTA\CHROMATOGRAFIA\LCMS\BABJAK\VPEN_CONSISTENT_CB45MIN_C8GNEG.D MM-ES+APCI, Neg
Norm.
Max: 65421
323.1
245.2
249.1
289.1
325.1
379.1
m/z

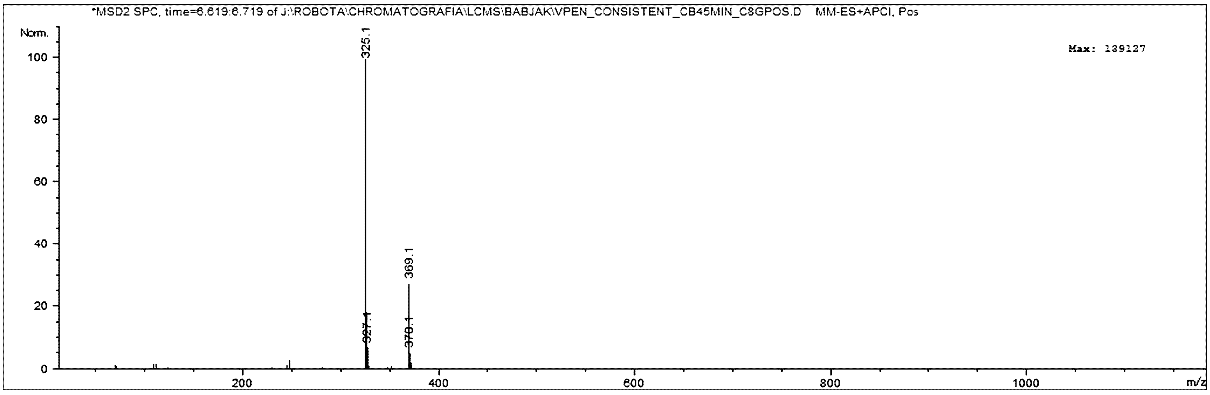
*MSD2 SPC, time=6.619:6.719 of J:\ROBOTA\CHROMATOGRAFIA\LCMS\BABJAK\VPEN_CONSISTENT_CB45MIN_C8GPOS.D MM-ES+APCI, Pos
Norm.
Max: 139127
325.1
369.1
327.1
370.1
m/z

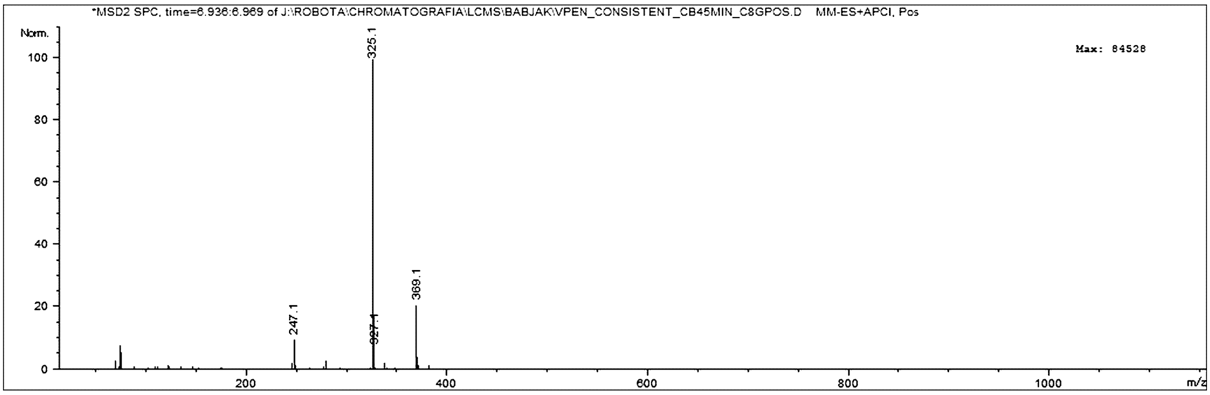

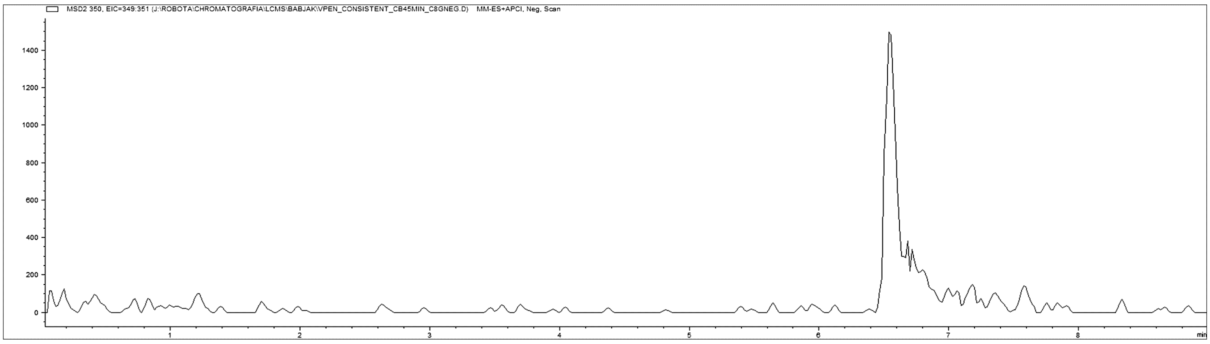

Figure 2 EIC signal for m/z 349-351 in the chromatogram with negative ionization, corresponding to the penillic intermediate.

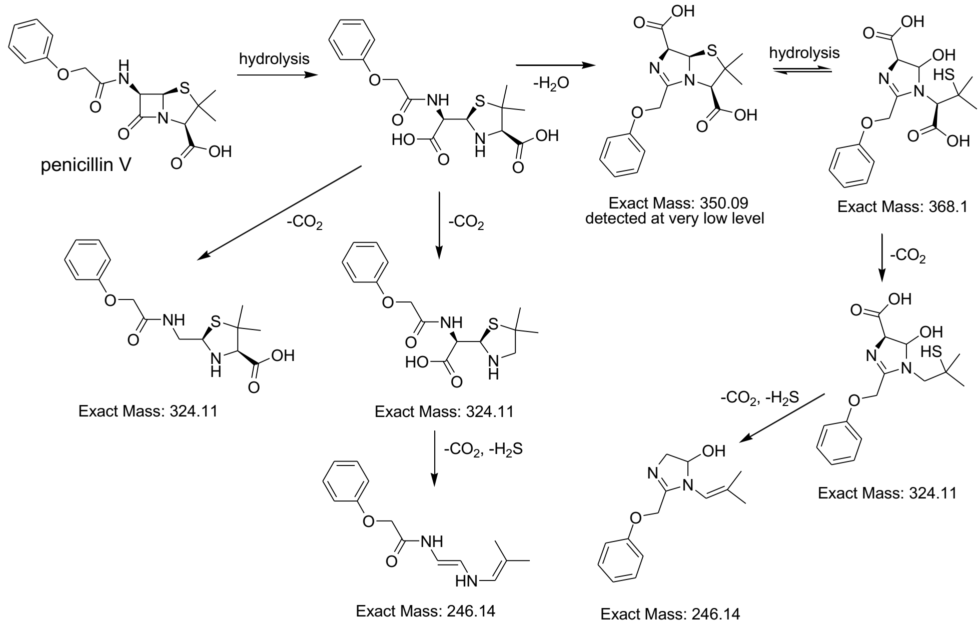

Meropenem decay promoted by B. Cereus metallo-lactamase, assayed by LCMS

The analysis with positive ionization was captured directly after the incubation period (injected after 45 min of incubation at RT), negative ionization experiment started 60 min after the incubation was started. Meropenem decomposed completely during this period. Only one major peak with slightly higher polarity was formed. Observed ions were corresponding to the expected hydrolytic pathway for all carbapenems: beta-lactam ring opening followed by retro-aldol elimination of acetaldehyde. The product then can undergo series of decarboxylations during the ionization process. Weak signal with RSH sensing probe can be explained by formation of the thiol intermediate with m/z 175.1 in the positive mode.

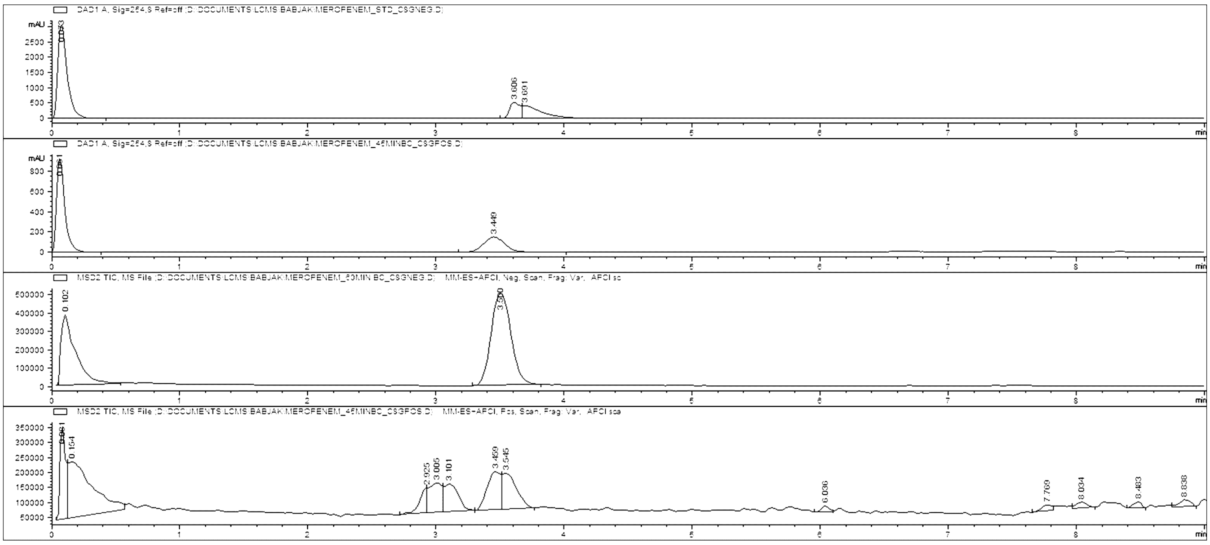

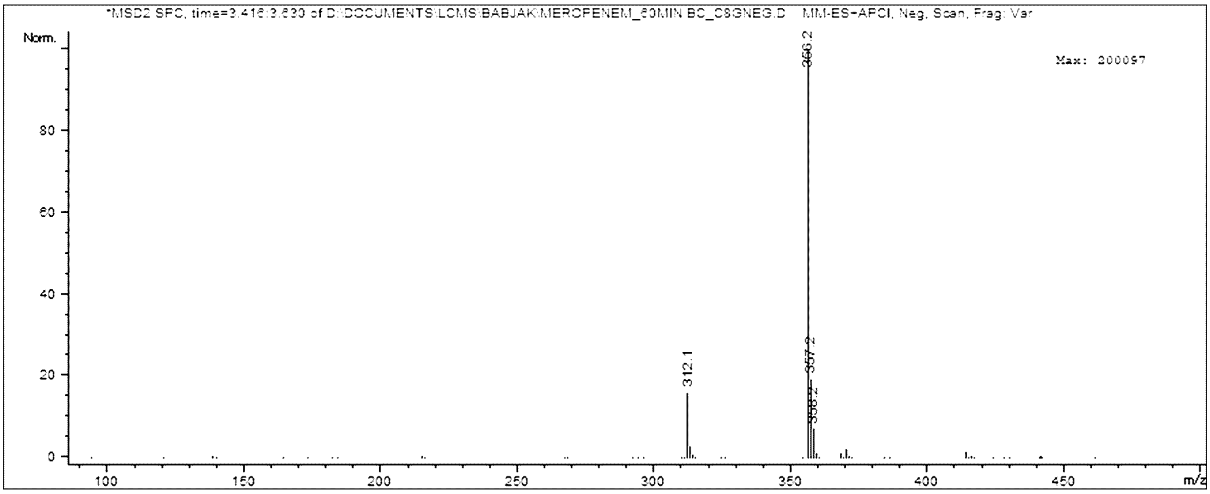

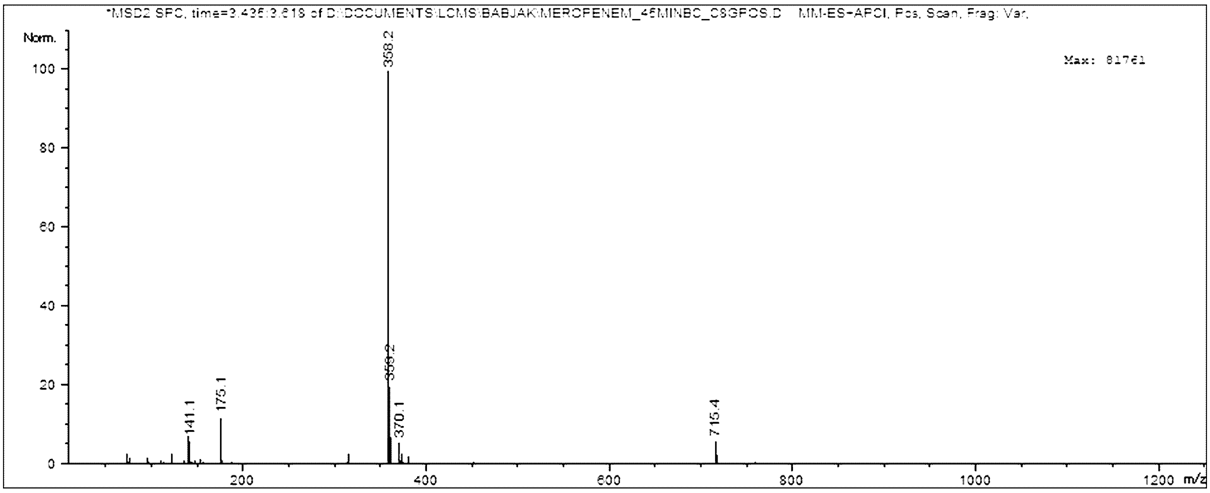

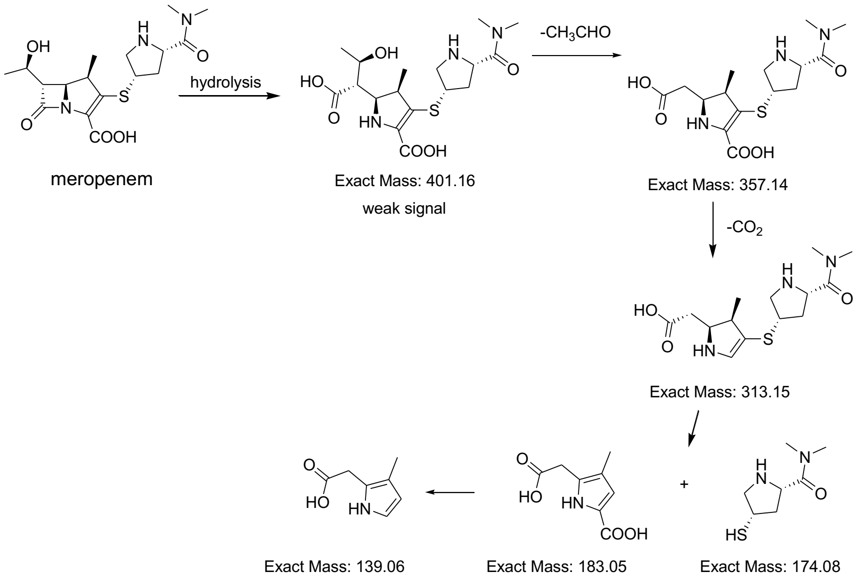

Cefotaxime decay promoted by B. Cereus metallo-lactamase, assayed by LCMS

The analysis with positive ionization was captured directly after the incubation period (injected after 50 min of incubation at RT), negative ionization experiment started 70 min after the incubation was started. Meropenem decomposed completely during this period. Only one broad major peak with slightly lower polarity was formed (5.22 min). MS signals of both polarities were very weak, which is given by zwitterion character of most molecules. Observed ions are corresponding with expected hydrolytic pathway: the acetoxy group eliminates along with the hydrolysis of the beta lactam ring. The gamma-lactone intermediate is easy detectable by its decarboxylated fragment ion (m/z 368 in acquired negative MS spectra). Under the positive ionization, m/z 201 and 101 are most remarkable, corresponding to the acylamide abstracted from the 7-position and its 2-aminothiazolyl fragment. Clearly, no formation of RSH or even H_2S is allowed during this hydrolytic decomposition, which agrees with poor signal measured by thiol sensing luminescent probes.

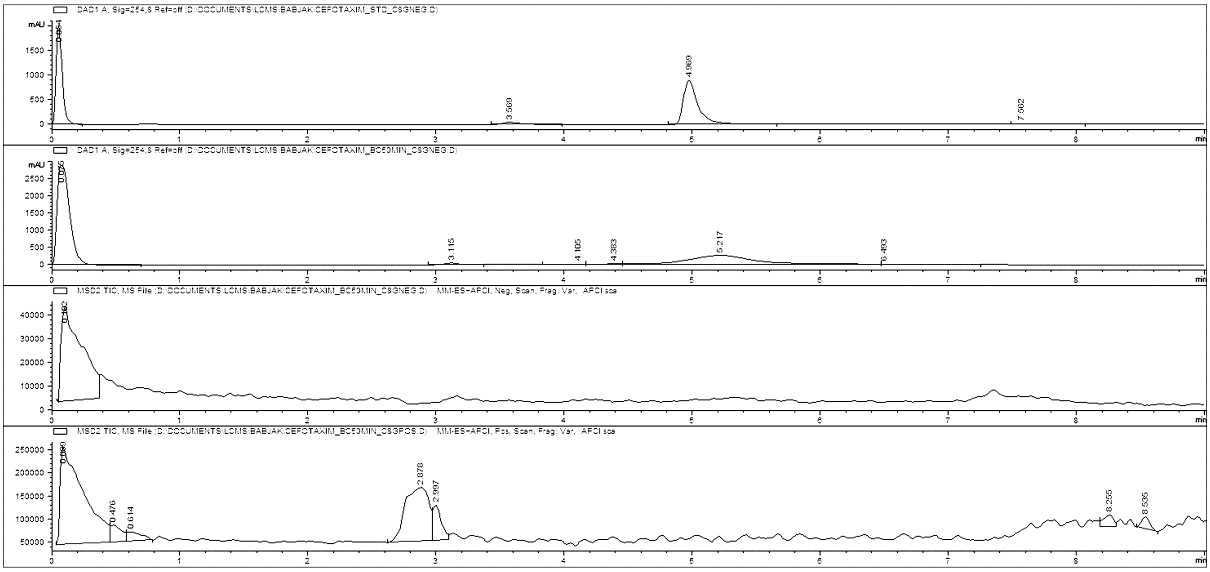

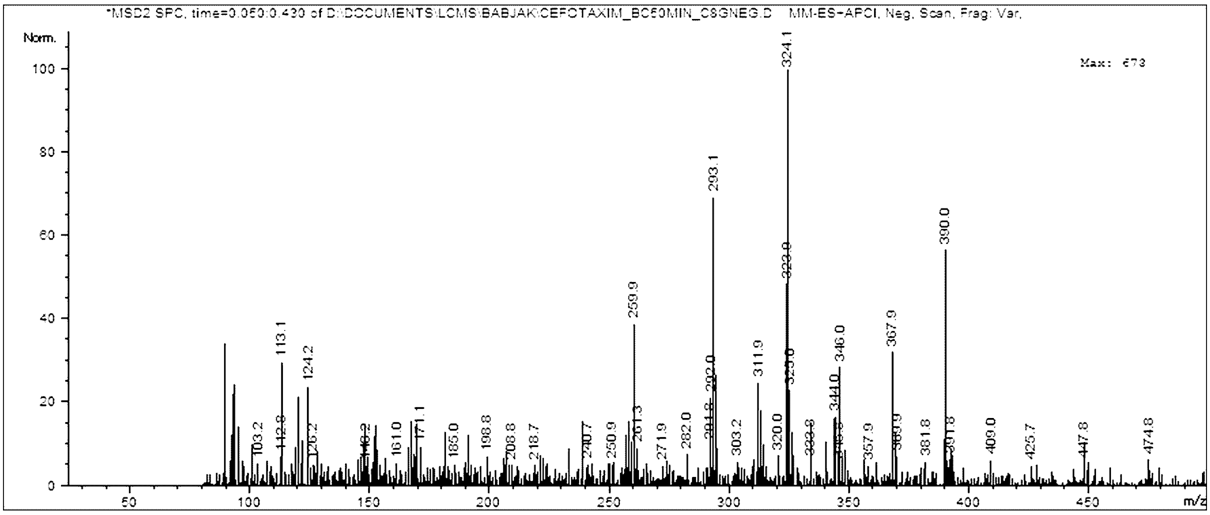
*MSD2 SPC, time=0.050:0.430 of D:\DOCUMENTS\LCMS\BABJAK\CEFOTAXIM_BC50MIN_CSGNEG.D   MM-ES+APCI, Neg, Scan, Frag: Var,
Norm.
Max: 673
m/z

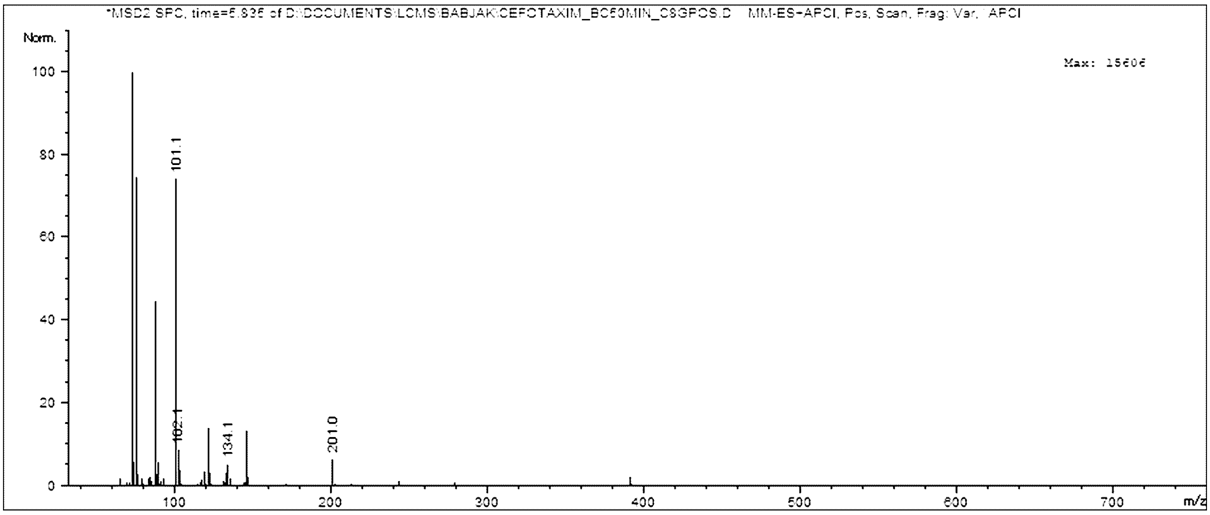
*MSD2 SPC, time=5.835 of D:\DOCUMENTS\LCMS\BABJAK\CEFOTAXIM_BC50MIN_CSGPOS.D   MM-ES+APCI, Pos, Scan, Frag: Var, APCI
Norm.
Max: 15606
m/z

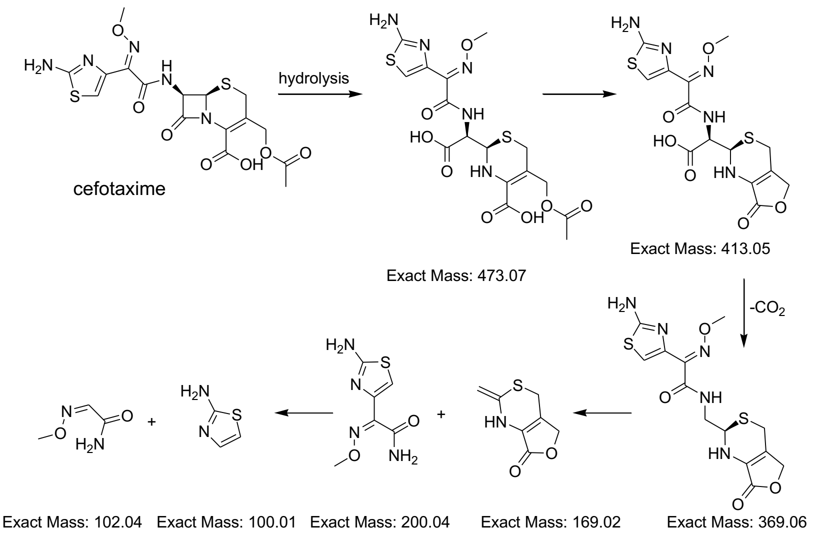

Imipenem decay promoted by B. Cereus metallo-lactamase, assayed by LCMS

The analysis with positive ionization was captured directly after the incubation period (injected after 70 min of incubation at RT), negative ionization experiment started after 90 min after the incubation was started. Imipenem decomposed completely during this period. Only one major peak with slightly higher polarity was formed (2.94 min). According to MS signals, imipenem undergoes lactamase mediated transformation quite in accord with other penems: first the lactamase opens the lactam ring and then catalyses the retro-aldol reaction. Formed product is then fragmented in MS ionization chamber through NH3 eliminating steps forming isocyanide fragments, which in parallel decarboxylate. No product nor fragment possessing SH group was detected, which explains low signal after treatment with the RSH sensing probes.

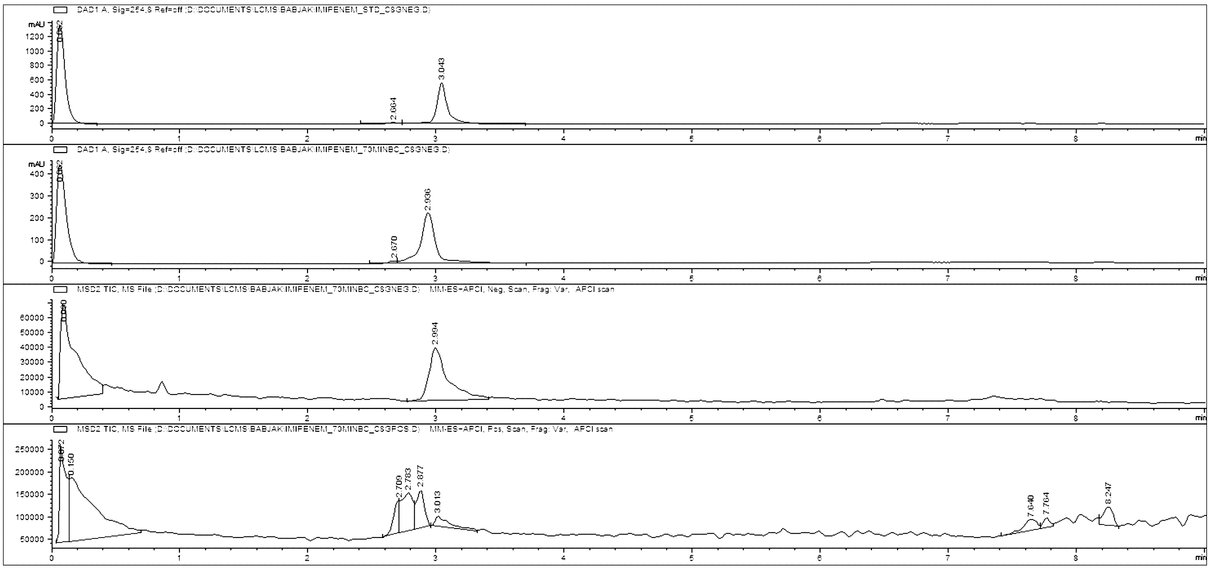

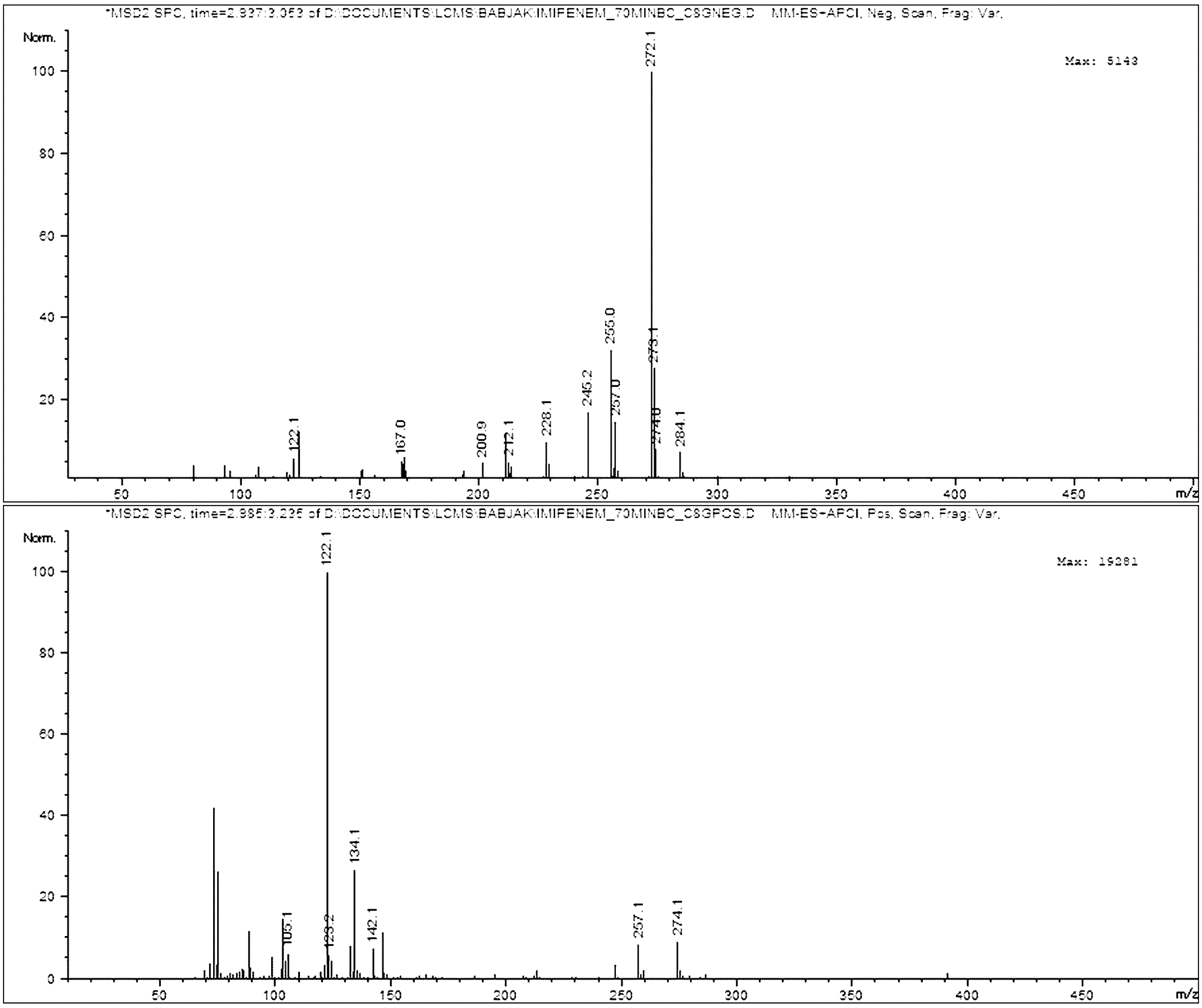
*MSD2 SPC, time=2.837:3.053 of D:\DOCUMENTS\LCMS\BABJAK\IMIPENEM_70MINBC_CSGNEG.D MM-ES+APCI, Neg, Scan, Frag: Var,
Norm.
Max: 5143
272.1
255.0
273.1
245.2
257.0
228.1
274.0
284.1
122.1
167.0
200.9
212.1
m/z
*MSD2 SPC, time=2.895:3.235 of D:\DOCUMENTS\LCMS\BABJAK\IMIPENEM_70MINBC_CSGPOS.D MM-ES+APCI, Pos, Scan, Frag: Var,
Norm.
Max: 19281
122.1
134.1
105.1
123.2
142.1
257.1
274.1
m/z

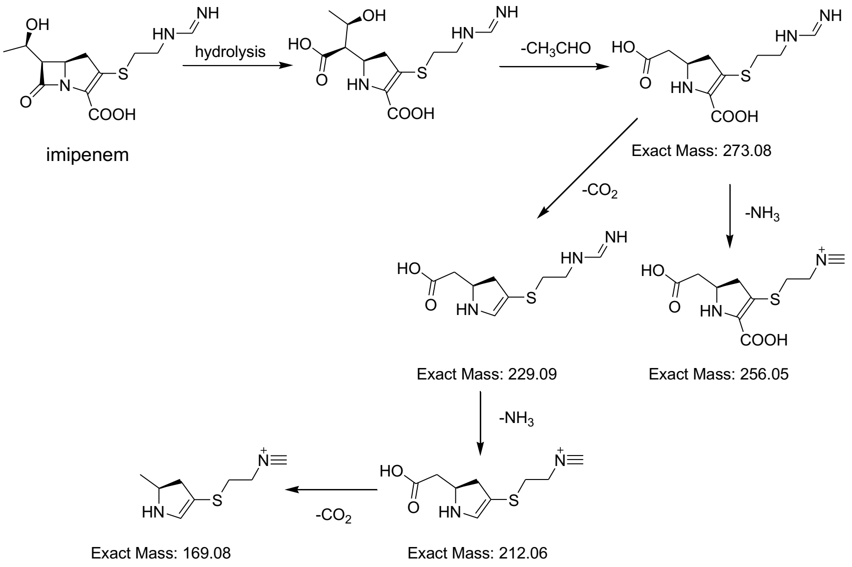
imipenem
hydrolysis
-CH3CHO
Exact Mass: 273.08
-CO2
-NH3
Exact Mass: 229.09
Exact Mass: 256.05
-NH3
-CO2
Exact Mass: 169.08
Exact Mass: 212.06

Ceftizoxime decay promoted by B. Cereus metallo-lactamase, assayed by LCMS

The analysis with positive ionization was captured directly after the incubation period (injected after 80 min of incubation at RT), negative ionization experiment started 100 min after the incubation was started. Ceftizoxime decomposed completely during this period. One major peak with lower polarity was formed (5.25 min) and one minor peak with slightly higher polarity (4.93 min). As the tested sample was the sodium salt of ceftizoxime, M+Na adducts were observed in higher intensity. Major decomposition pathway follows the cefotaxime scheme with one important exception: detected ions with m/z 243 and m/z 134 in the positive mode signal can be explained by the opening of the dihydrothiazine ring, when an aldehyde structure and corresponding enamine-thiol is formed. We believe this process can occur at lower temperatures as well and explains high signal strength with RSH sensing probes.

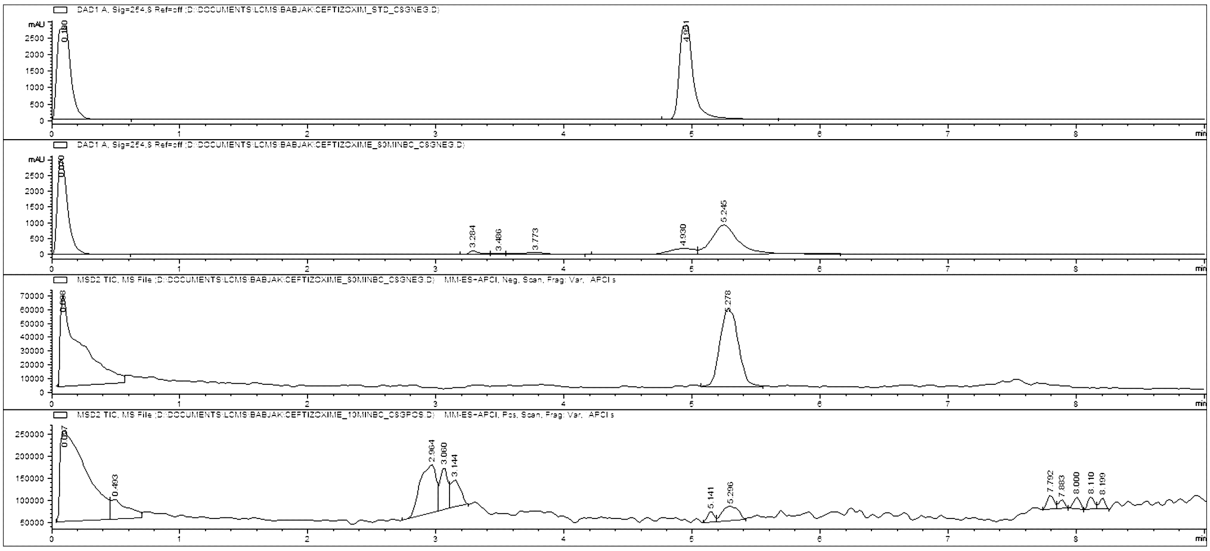

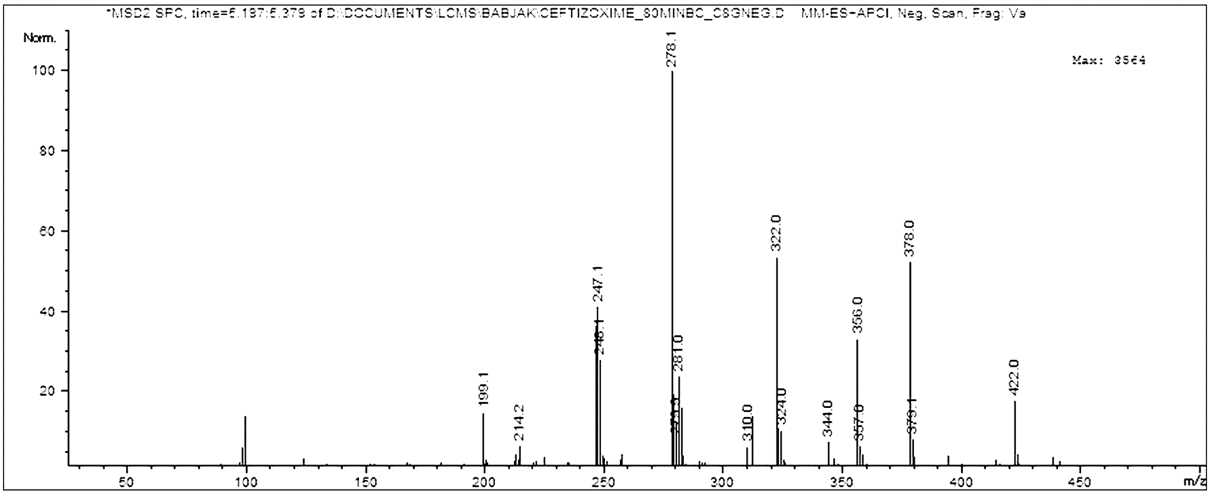

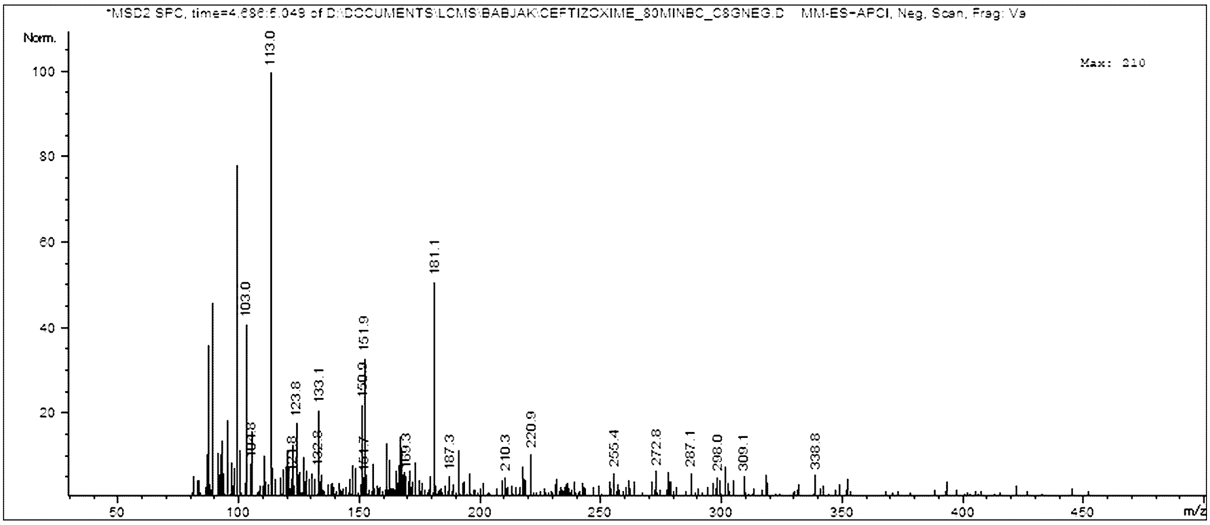
*MSD2 SPC, time=4.686:5.049 of D:\DOCUMENTS\LCMS\BABJAK\CEFTIZOXIME_80MINBC_C8GNEG.D MM-ES+APCI, Neg, Scan, Frag: Va
Norm.
Max: 210
113.0
103.0
181.1
151.9
150.9
123.8
133.1
220.9
210.3
187.3
255.4
272.8
287.1
298.0
309.1
338.8
m/z

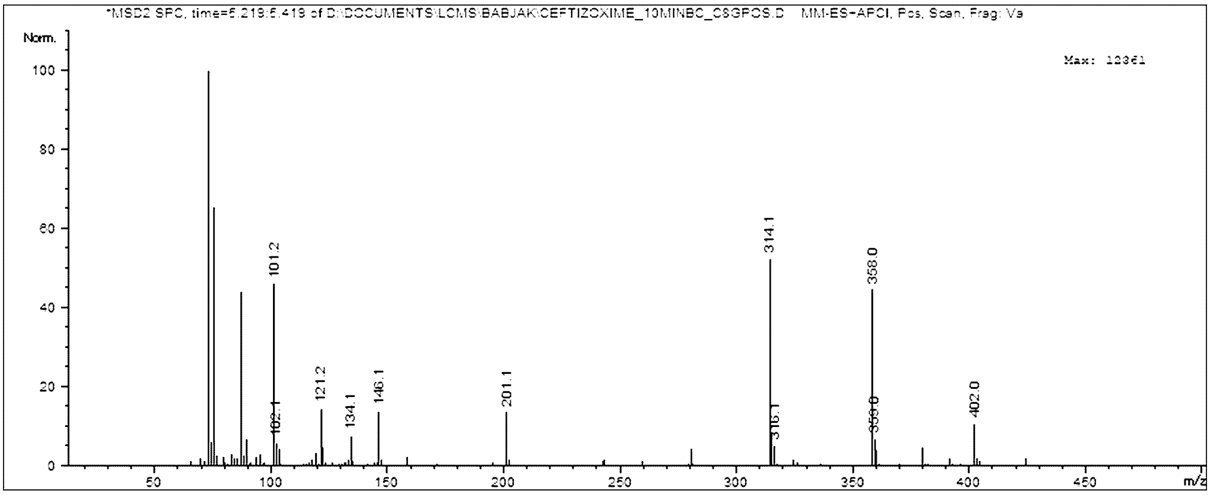
*MSD2 SPC, time=5.219:5.419 of D:\DOCUMENTS\LCMS\BABJAK\CEFTIZOXIME_10MINBC_C8GPOS.D MM-ES+APCI, Pos, Scan, Frag: Va
Norm.
Max: 12361
101.2
314.1
358.0
121.2
134.1
146.1
201.1
402.0
m/z

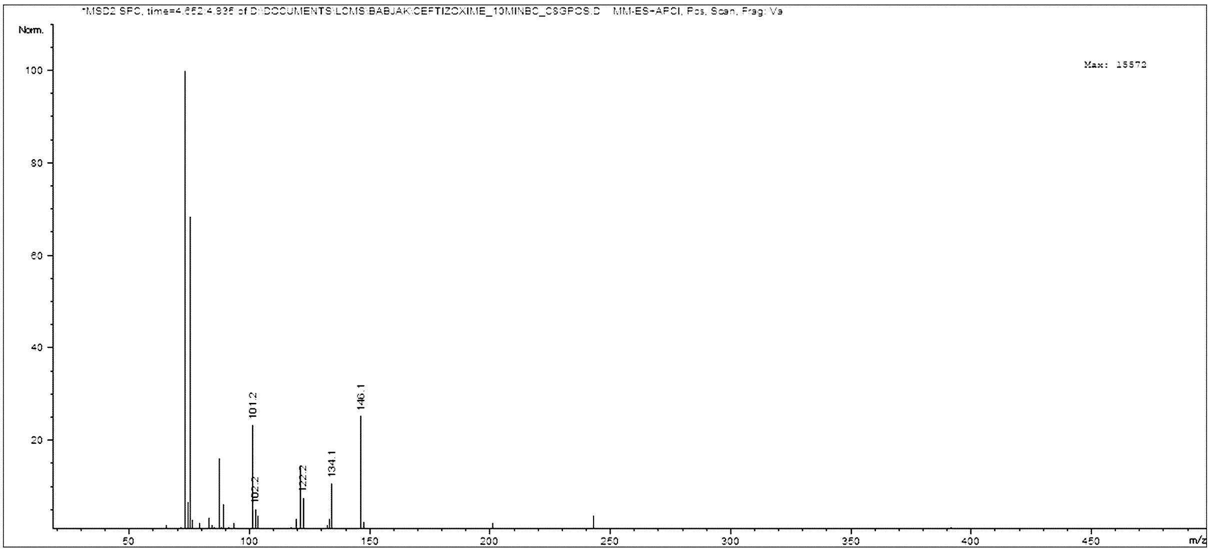
*MSD2 SPC, time=4.652:4.935 of D:\DOCUMENTS\LCMS\BABJAK\CEFTIZOXIME_10MINBC_C8GPOS.D MM-ES+APCI, Pos, Scan, Frag: Va
Norm.
Max: 15572
101.2
146.1
134.1
122.2
102.2
m/z

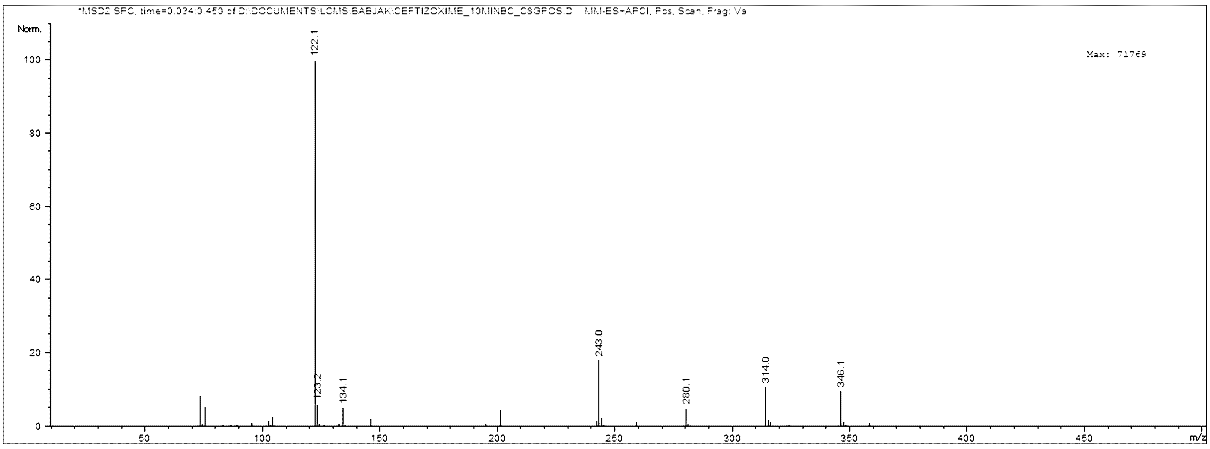

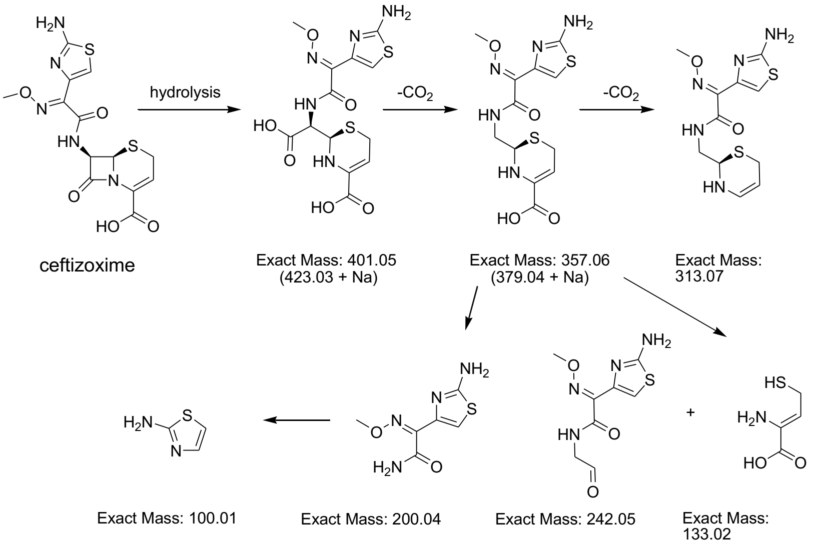

Ceftazidime decay promoted by B. Cereus metallo-lactamase, assayed by LCMS

The analysis with positive ionization was captured directly after the incubation period (injected after 80 min of incubation at RT), negative ionization experiment started 100 min after the incubation was started. Ceftazidime decomposed completely during this period. One major peak with higher polarity was formed (3.92 min) and one minor peak with even higher polarity (3.18 min). MS signal of both polarities was weak, which was caused by zwitterion character of relevant structures, and heavily fragmented, which is resulting from fragile structure. Pyridinium ring eliminates readily after the lactam ring was opened and the oxime side chain is also steady for fast decarboxylation in the ionization chamber. One tentative thiol structure with m/z 146 was identified, which could eventually explain weak luminescent signal observed in this case.

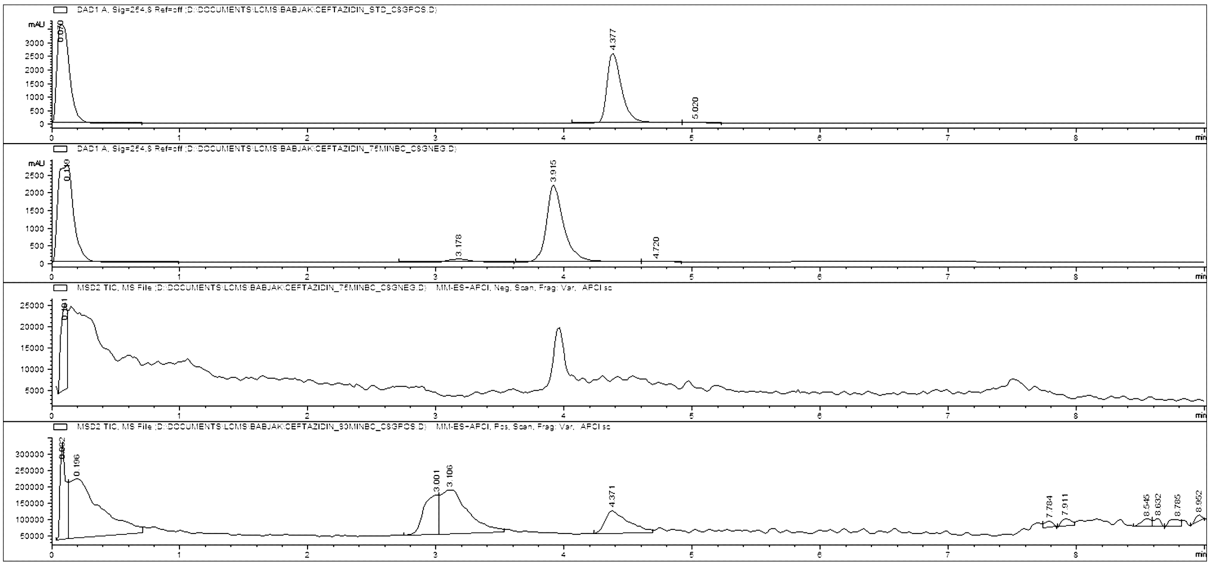
DAD1 A, Sig=254,8 Ref=off (D:\DOCUMENTS\LCMS\BABJAK\CEFTAZIDIN_STD_CSGPOS.D)
4.377
5.020
DAD1 A, Sig=254,8 Ref=off (D:\DOCUMENTS\LCMS\BABJAK\CEFTAZIDIN_75MINBC_CSGNEG.D)
3.915
3.178
4.720
MSD2 TIC, MS File (D:\DOCUMENTS\LCMS\BABJAK\CEFTAZIDIN_75MINBC_CSGNEG.D) MM-ES+APCI, Neg, Scan, Frag: Var, APCI sc
MSD2 TIC, MS File (D:\DOCUMENTS\LCMS\BABJAK\CEFTAZIDIN_90MINBC_CSGPOS.D) MM-ES+APCI, Pos, Scan, Frag: Var, APCI sc
0.196
3.001
3.106
4.371
7.784
7.911
8.545
8.632
8.785
8.952
min

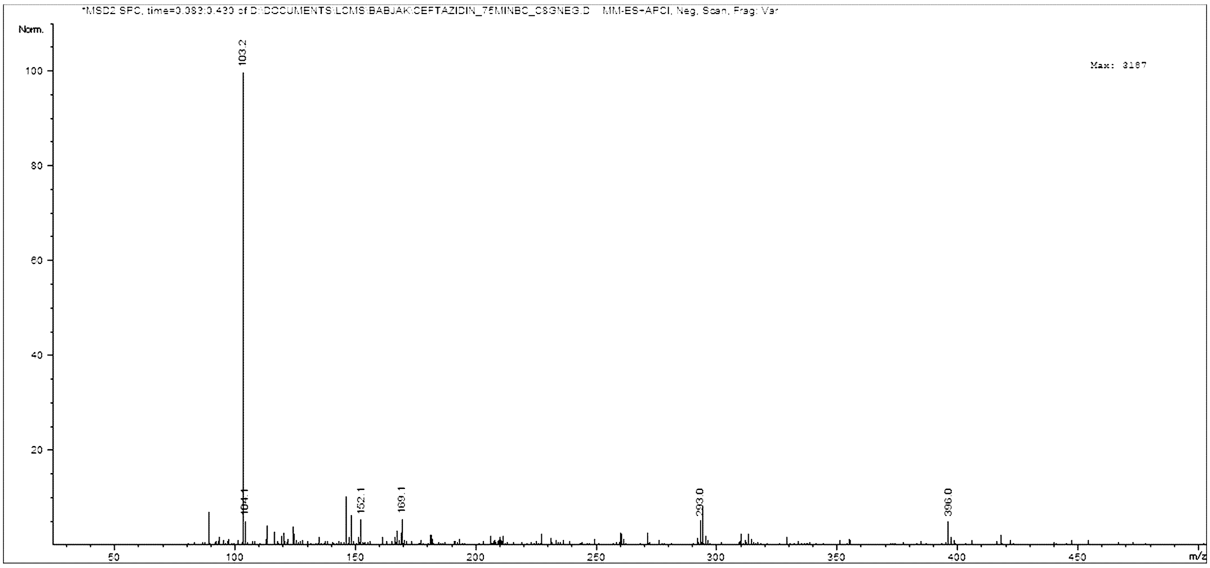
*MSD2 SPC, time=0.083:0.430 of D:\DOCUMENTS\LCMS\BABJAK\CEFTAZIDIN_75MINBC_CSGNEG.D MM-ES+APCI, Neg, Scan, Frag: Var
Norm.
Max: 3187
103.2
104.1
152.1
169.1
293.0
396.0
m/z

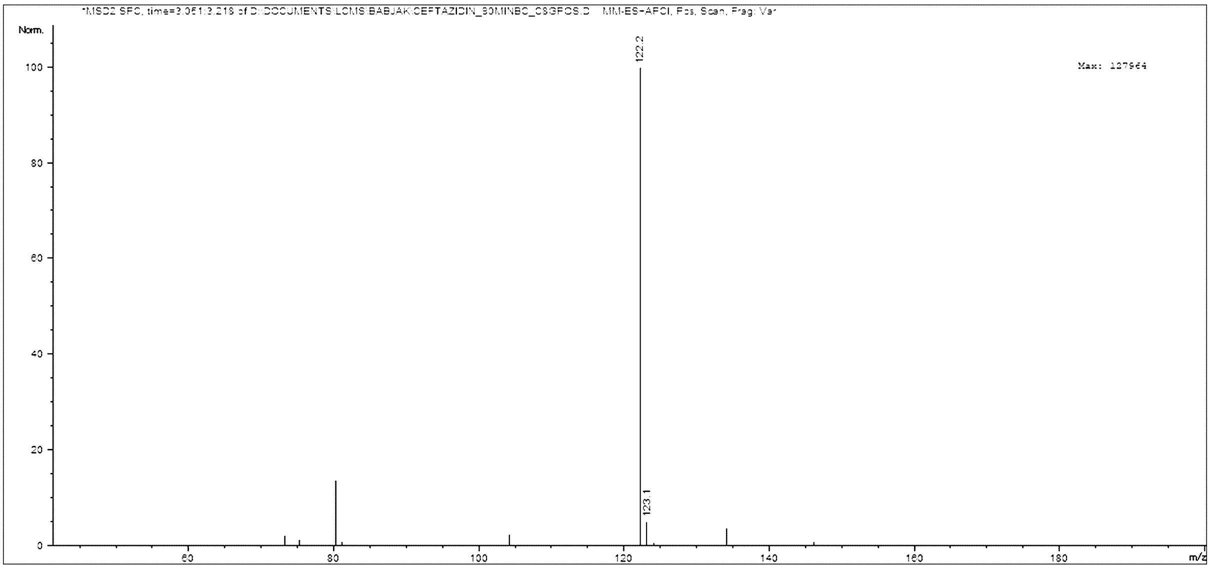
*MSD2 SPC, time=3.051:3.218 of D:\DOCUMENTS\LCMS\BABJAK\CEFTAZIDIN_90MINBC_CSGPOS.D MM-ES+APCI, Pos, Scan, Frag: Var
Norm.
Max: 127964
122.2
123.1
m/z

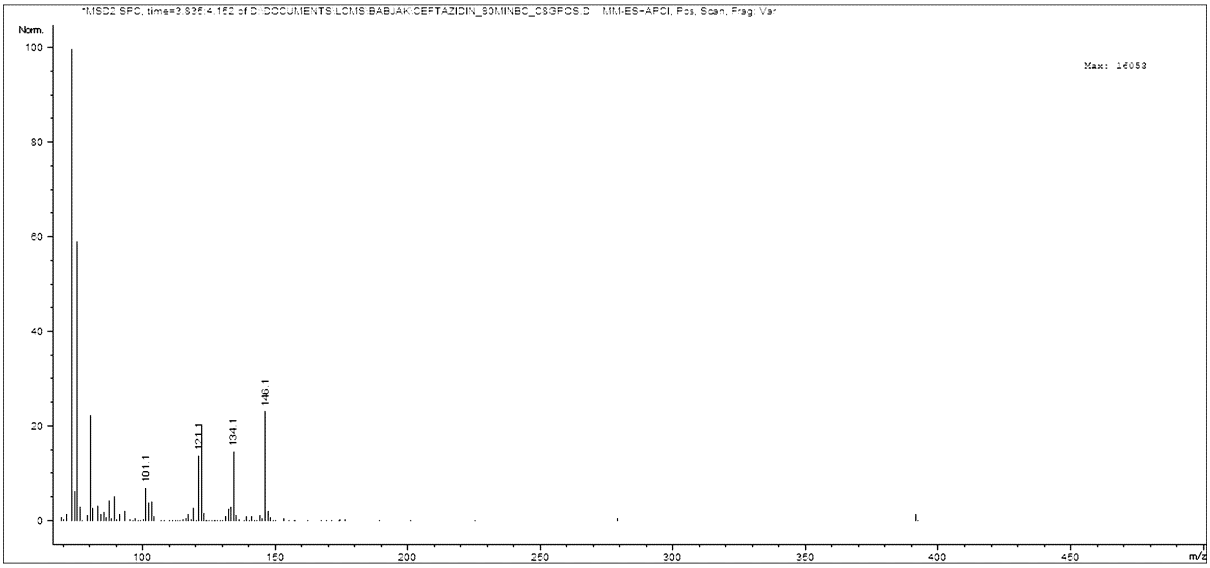

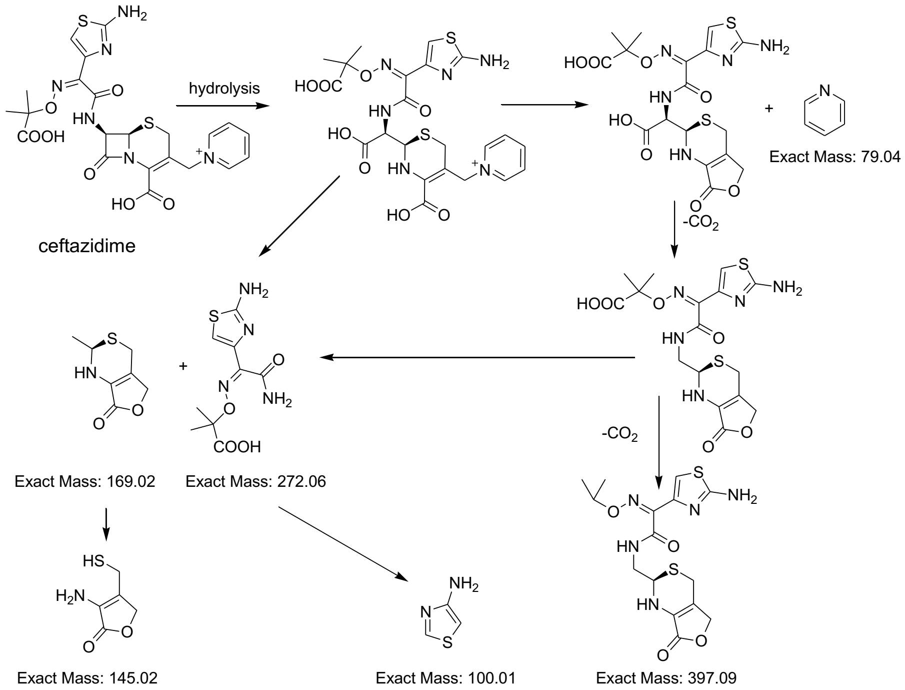

Cefazoline decay promoted by B. Cereus metallo-lactamase, assayed by LCMS

50 min of incubation at RT), negative ionization experiment started 70 min after the incubation was started. Cefazoline decomposed completely during this period. Two small peaks were formed at The analysis with positive ionization was captured directly after the incubation period (injected after 50 min of incubation at RT), negative ionization experiment started 70 min after the incubation was started. Cefazoline decomposed completely during this period. Two small peaks were formed at 5.14 and 5.51 min. In this case the captured ions clearly display the mechanism of H_2S release through hydrolytic opening of the dihydrothiazine ring, producing positive ions with m/z 263, 279 and 295. 5-Methyl-1,3,4-thiadiazole-2-thiol is another RSH component generated quickly after the lactam ring was

hydrolysed, nevertheless this electron-poor thiol has only minimal triggering effect on used hydrogen sulphide sensing probes.

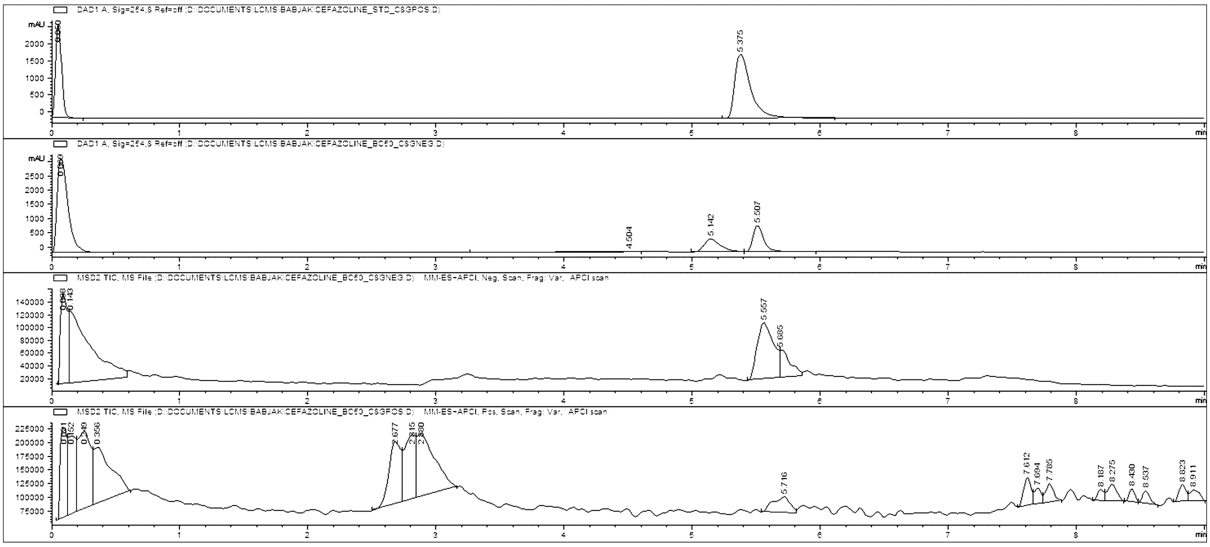

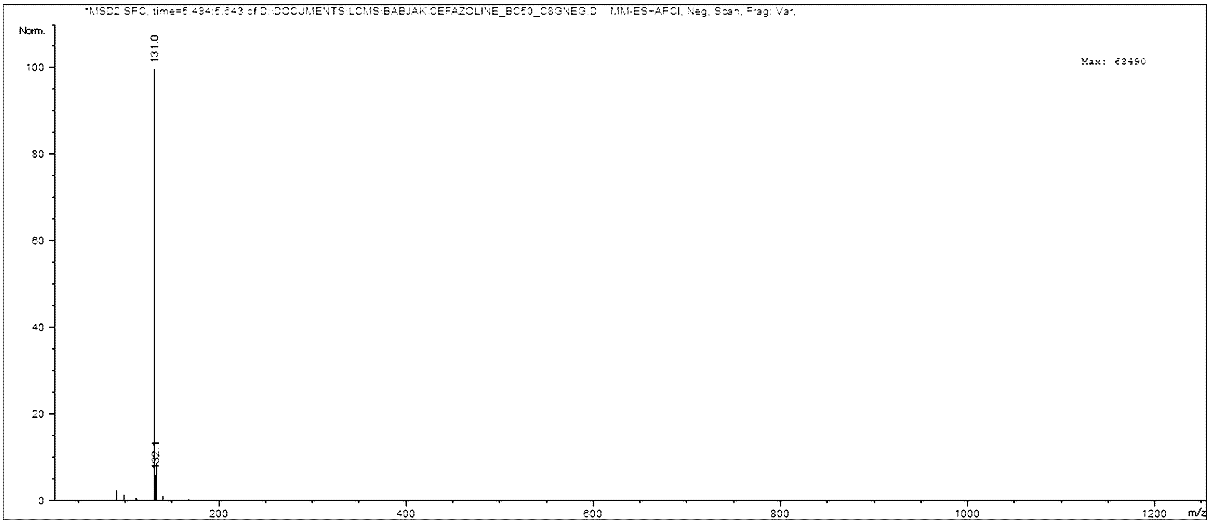

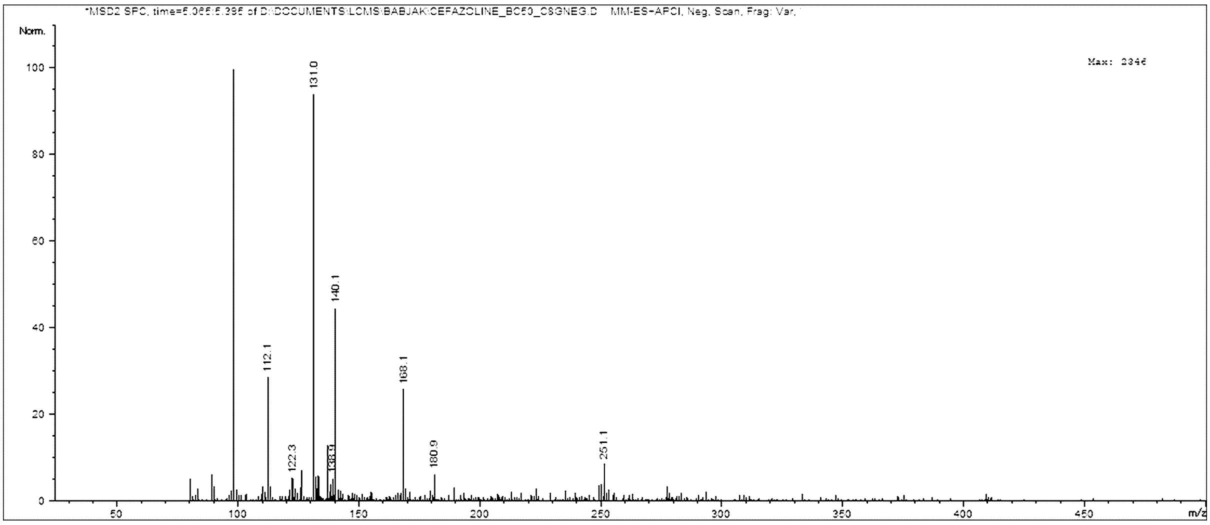

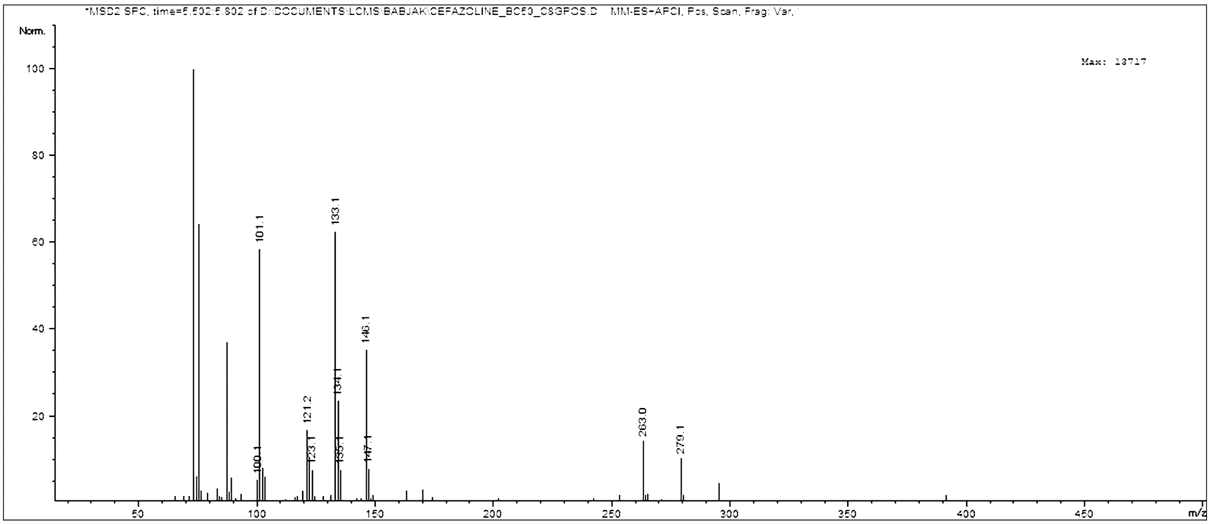
*MSD2 SPC, time=5.502:5.802 of D:\DOCUMENTS\LCMS\BABJAK\CEFAZOLINE_BC50_C8GPOS.D MM-ES+APCI, Pos, Scan, Frag: Var,
Norm.
Max: 13717
101.1
133.1
146.1
121.2
134.1
123.1
135.1
147.1
100.1
263.0
279.1
m/z

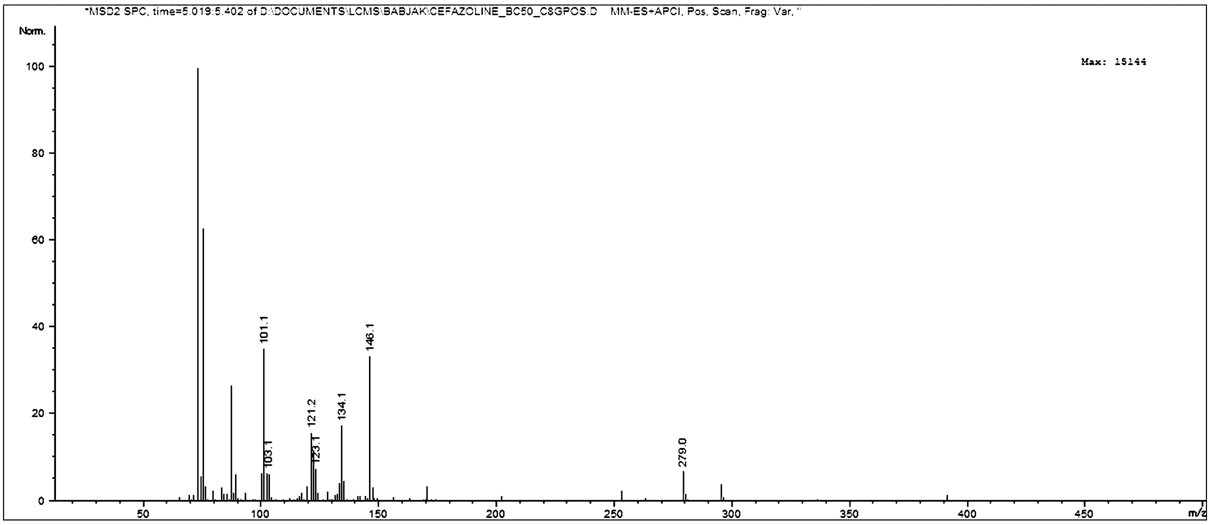
*MSD2 SPC, time=5.019:5.402 of D:\DOCUMENTS\LCMS\BABJAK\CEFAZOLINE_BC50_C8GPOS.D MM-ES+APCI, Pos, Scan, Frag: Var,
Norm.
Max: 15144
101.1
146.1
121.2
134.1
123.1
103.1
279.0
m/z

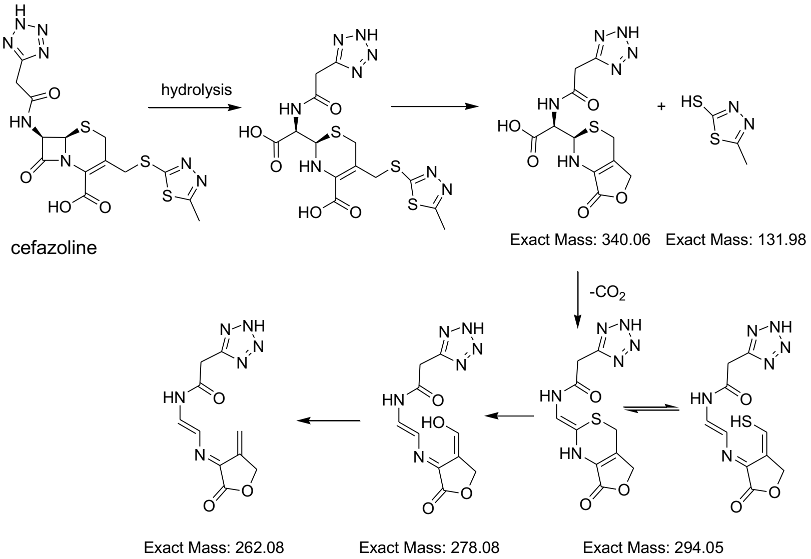
hydrolysis
cefazoline
Exact Mass: 340.06
Exact Mass: 131.98
-CO2
Exact Mass: 262.08
Exact Mass: 278.08
Exact Mass: 294.05
